# Identification of *Streptococcus suis* putative zoonotic virulence factors: A systematic review and genomic meta-analysis

**DOI:** 10.1080/21505594.2021.1985760

**Published:** 2021-11-25

**Authors:** Thomas J. Roodsant, Boas C.L. Van Der Putten, Sara M. Tamminga, Constance Schultsz, Kees C.H. Van Der Ark

**Affiliations:** a Amsterdam UMC, University of Amsterdam, Department of Global Health-Amsterdam, Institute for Global Health and Development, Amsterdam, Netherlands; b Department of Medical Microbiology, Amsterdam UMC, University of Amsterdam, Amsterdam, Netherlands

**Keywords:** Streptococcus suis, zoonoses, virulence factor, systematic review, meta-analysis

## Abstract

*Streptococcus suis* is an emerging zoonotic pathogen. Over 100 putative virulence factors have been described, but it is unclear to what extent these virulence factors could contribute to zoonotic potential of *S. suis*. We identified all *S. suis* virulence factors studied in experimental models of human origin in a systematic review and assessed their contribution to zoonotic potential in a subsequent genomic meta-analysis. PubMed and Scopus were searched for English-language articles that studied *S. suis* virulence published until 31 March 2021. Articles that analyzed a virulence factor by knockout mutation, purified protein, and/or recombinant protein in a model of human origin, were included. Data on virulence factor, strain characteristics, used human models and experimental outcomes were extracted. All publicly available *S. suis* genomes with available metadata on host, disease status and country of origin, were included in a genomic meta-analysis. We calculated the ratio of the prevalence of each virulence factor in human and pig isolates. We included 130 articles and 1703 *S. suis* genomes in the analysis. We identified 53 putative virulence factors that were encoded by genes which are part of the *S. suis* core genome and 26 factors that were at least twice as prevalent in human isolates as in pig isolates. Hhly3 and NisK/R were particularly enriched in human isolates, after stratification by genetic lineage and country of isolation. This systematic review and genomic meta-analysis have identified virulence factors that are likely to contribute to the zoonotic potential of *S. suis*.

## Introduction

*Streptococcus suis* is an opportunistic pathogen in pigs and can cause zoonotic infections that often result in meningitis [1,2]. *S. suis* zoonotic infections occur worldwide with the highest reported incidence in Thailand, Vietnam and The Netherlands[1]. Close contact with pigs and consumption of undercooked pork have been identified as important risk factors for zoonotic *S. suis* infections[1]. The emergence of zoonotic clones has been demonstrated and led to new insights in the evolution of *S. suis*’ population structure[3], but the virulence factors involved in zoonotic potential of *S. suis* are not well understood.

*S. suis* of multiple serotypes from different phylogenetic groups (clonal complexes) are found in healthy and diseased pigs, but human infections are predominantly caused by strains from clonal complex 1 and serotypes 2 or 14 [1,4]. Distinct stages in the pathogenesis of *S. suis* infections in humans include the adhesion and translocation across mucosal surface particularly in case of foodborne infection, survival in blood, and translocation across the blood brain barrier in case of meningitis [5]. Over 100 putative *S. suis* virulence factors have been described that may contribute to the pathogenesis of infection in pigs [4,6]. Although many of these virulence factors were identified in *in vitro* models of human origin, their contribution to *S. suis* zoonotic potential has not been studied.

We performed a systematic review of *S. suis* virulence factors studied in *in vitro* models of human origin. In a subsequent genomic meta-analysis we determined if these putative virulence factors are encoded by the *S. suis* core or accessory genome and identified those virulence factors that may contribute to the pathogenesis of zoonotic infection, designated putative zoonotic virulence factors (PZVFs).

## Methods

### Definitions

Virulence factors can be defined as “molecules produced by pathogens that contribute to the pathogenicity of the organism by allowing its establishment, replication, dissemination and persistence in the host” [4]. Here, we define a PZVF as a virulence factor of a bacterial pathogen from an animal reservoir that contributes to pathogenicity in the human host specifically. We define human models as *in vitro* models of human origin, including cell lines in continuous culture of human origin, human primary cells, human blood, human blood components, human extracellular matrix proteins, and the zebrafish human streptococcal infection model [7].

Search strategy and selection criteria

The systematic review was performed according to Preferred Reporting Items for Systematic Reviews and Meta-Analyses (PRISMA) guidelines [8]. TR searched PubMed and Scopus for primary research articles published until 31 March 2021 describing *S. suis* and virulence in the title and/or abstract using Pubmed PubReMiner to generate the search query (appendix S1 p1) [9]. References were downloaded and duplicates were removed using Endnote (9.3.3), Mendeley (1.19.8) and a manual search. TR and KA independently screened all titles and abstracts and selected articles that mentioned a host (e.g. human or pig) and *S. suis* and both agreed on the final selection for full text screening, which was done by TR. Studies were included when a virulence factor was evaluated in a human model and the virulence factor was studied in an isogenic knockout (KO) mutant, as recombinant protein, and/or as purified protein. Articles were excluded when the full text was unavailable in English. Experimental outcomes included bacterial binding of host proteins, adhesion, invasion, translocation, survival and immune cell responses.

### Data extraction

TR and ST extracted data from the included articles in a pre-specified table in Microsoft Excel 2016 (appendix S1 p2, appendix S2), followed by an overall curation of extracted data by KA. In short, we extracted information on virulence factor analysis approach (KO, recombinant protein and/or purified protein), *S. suis* strain characteristics, applied *in vitro* models, experimental outcomes and NCBI protein ID. If NCBI protein ID was not stated, the NCBI protein ID was searched manually using available data such as primer sequences, gene names or protein sequences. Experimental outcomes for single virulence factors studied in at least 5 articles were summarized and compared. As part of a critical appraisal, data on growth rates of wildtype, isogenic KO and complementation mutants were extracted from the articles or articles’ references. In addition, the number of *S. suis* strains analyzed in each study was recorded.

### Bacterial genome meta-analysis

We downloaded all BioSample records from NCBI mentioning “*Streptococcus suis”* (final date 31–01-2020). Missing metadata were searched in the corresponding publications and pubMLST [10], and added. Genomes were included if at least metadata on host, host health status, and country of origin were available (see appendix 1 p3). The curated set of assembled genomes with corresponding metadata was deposited on Zenodo (10.5281/zenodo.4686597).

The presence of a virulence factor in *S. suis* isolates was determined by mapping its protein sequence with a minimal protein identity of 95% and query coverage of 60% on the translated *S. suis* genome assemblies (see appendix 1 p3 [11–14]). We defined the core genome as all genes present in ≥95% of the isolates whilst the remaining genes constitute the accessory genome.

We calculated the ratio of the prevalence of each virulence factor in *S. suis* populations isolated from human, and healthy and diseased pigs respectively. A virulence factor was considered a PZVF if the prevalence ratio > 2. A stratified analysis was performed for the main zoonotic *S. suis* lineage (clonal complex 1) and the countries contributing most human isolates (China, Vietnam).

## Results

**Figure 1.**
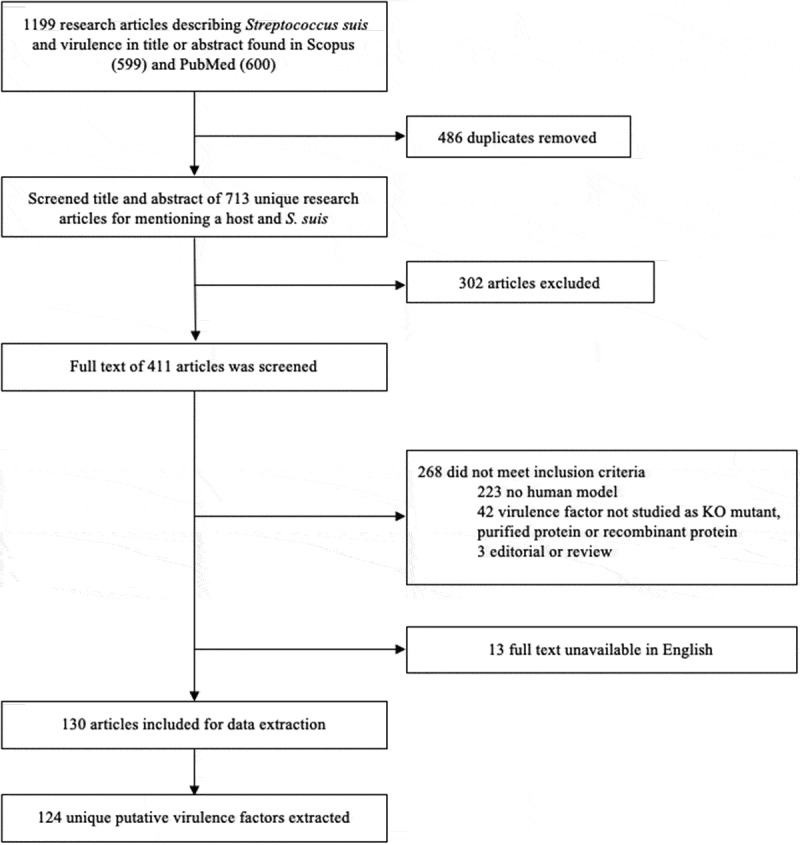
PRISMA flow diagram

Title and abstract of 713 unique records were screened and 411 articles were selected for full text screening. Of these 411, 268 articles did not meet inclusion criteria and 13 were excluded due to unavailability of full text in English. The 130 included articles described 124 different putative virulence factors (Figure 1).

Putative virulence factors were studied as purified protein (3), as recombinant protein (51), as (partial) isogenic KO (152), by blocking protein function with antibodies (3) or as a combination of these. For 56/152 (37%) of the isogenic KO mutants, changes in growth rate compared to parental wildtype were not assessed. For 72/152 (47%) growth rate of KO mutants was reported as unaffected and for 24/152 (16%) impaired growth was observed for the KO mutant. In only 43 (28%) studies the KO mutant was genetically complemented and three articles (2%) described complementation with a recombinant protein.

**Figure 2.**
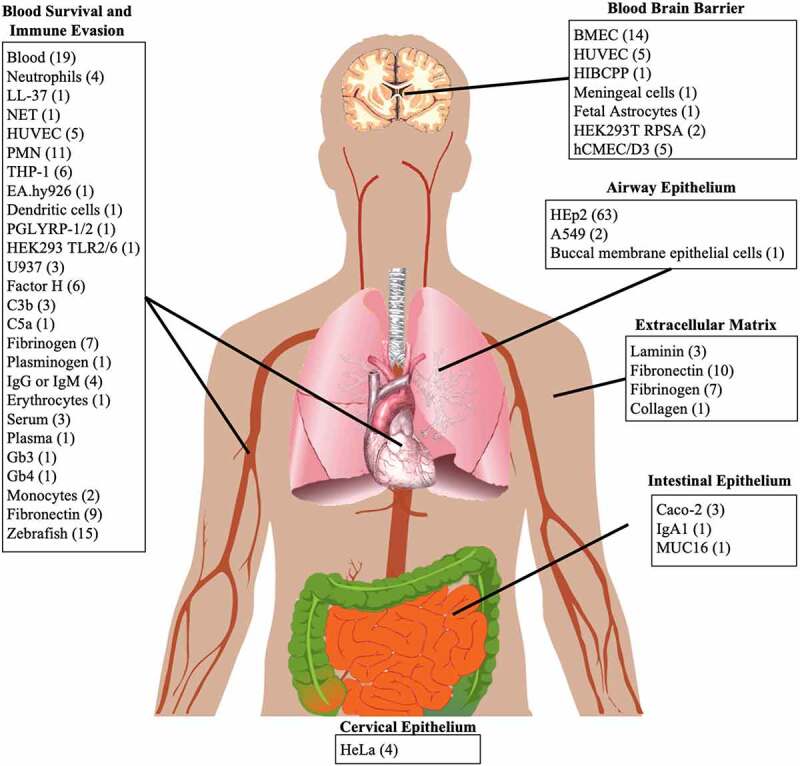
Grouping of human models to their respective human *S. suis* infection site. Number of articles per model is indicated between brackets

Models used to evaluate putative virulence factors were grouped based on the human body sites from which the model originated (Figure 2). The human epithelial HEp2 cell line was used in 63 out of 72 articles that studied adhesion, invasion or cell lysis induced by *S. suis* in a human epithelial model. Adhesion to extracellular matrix was studied in 13 different articles that used laminin (3), collagen (1), fibronectin (10) and/or fibrinogen (7). Survival in blood was studied in 70 articles using a diverse set of models, of which human whole blood (19), human (polymorphonuclear) neutrophils (15) and zebrafish (15) were most frequent. Human brain microvascular endothelial cells (BMECs) were used in 14 out of 25 articles that studied the role of a virulence factor in crossing the blood-brain barrier (BBB).

### Experimental outcomes

Five out of 124 (4%) putative virulence factors (appendix S1 p2, appendix S2) were studied in at least 5 articles and the experimental outcomes were summarized and compared for each factor to evaluate their contribution to zoonotic potential (appendix S1 p4-6).

#### Capsular polysaccharide (CPS)

The CPS forms the outer layer of bacteria and consists of repeating oligosaccharide subunits that differ in composition and linkage between *S. suis* serotypes [15,16]. The CPS decreases *S. suis* adherence to and invasion of human epithelial cells [17–21]. One study reported no effect of the CPS on adherence to cervical epithelial HeLa cells[22]. The CPS contributes to *S. suis* blood survival [21,23] and to immune evasion by decreasing phagocytosis [21,22,24–29], increasing intracellular survival in PMN [23], dampening the innate immune response and decreasing complement activation [25,26,28–30]. The CPS decreases *S. suis* adherence to and invasion of meningeal cells and fetal astrocytes [31] and the adherence to human umbilical vein endothelial cells (HUVEC) [21]. A CPS KO showed a trend of increased translocation across human choroid plexus papilloma cells (HIBCCP) [32]. The CPS decreases IL-6 and IL-8 secretion by BMECs, but MCP-1 secretion is unaffected [33]. Moreover, purified CPS induces PGE2 and MMP-9 secretion in macrophage-like U937 cells which increases BBB leakiness [25]. The CPS also binds fibrinogen [24].

#### Suilysin (Sly)

Suilysin is a cholesterol-dependent hemolysin secreted by *S. suis* that can form pores in eukaryotic cells by oligomerization in cellular membranes [34,35]. Sly can induce HEp2 cell lysis [36] and at subcytolitic concentrations contributes to HEp2 invasion [37]. Sly does not contribute to HEp2 cell adherence [37] or translocation across an intestinal epithelial Caco-2 monolayer [20]. In human blood, Sly induces TNFα release by monocytes [38], PMN degranulation [39], platelet-neutrophil complex formation [40] and its hemolytic activity causes inflammasome activation in macrophage-like THP-1 cells [28]. Sly induces the release of arachidonic acid in BMEC, which can enhance BBB permeability [41]. Sly does not affect adherence to meningeal cells, adherence to astrocytes or invasion of meningeal cells but does increase astrocytes invasion [31].

#### Muramidase release protein (MRP)

MRP is a cell-wall anchored protein similar to the fibronectin-binding protein of *Staphylococcus aureus* [42–45]. MRP can directly bind to HEp2 cells [46] and contributes to *S. suis* adhesion to HEp2 [47]. MRP was shown to bind fibrinogen [48–50] and MRP binding of fibrinogen contributes to blood survival, decreases PMN killing [48,49] and in brain microvascular endothelial hCMEC/D3 cells increases adhesion and translocation [51]. MRP with bound fibrinogen also decreases the adherens junction protein p120-catenin in hCMEC/D3 thus potentially increasing BBB permeability [51]. Besides fibrinogen, MRP was shown to bind factor H [50,52] and fibronectin [50].

#### Factor H binding protein (Fhb)

Fhb also named Streptococcal adhesin protein (SadP) is anchored in the cell wall and secreted [53]. Fhb can bind proteins from the host complement system as well as glycans [53–55]. Fhb can bind to Gb3 on human erythrocytes and a specific allele of Fhb (SadP_n_) can also bind to Gb4 [56]. Fhb contributes to *S. suis* adhesion to and translocation across a Caco-2 monolayer by binding to Gb3 [57]. Fhb can bind human factor H, which increases *S. suis* adherence to airway epithelial A549 cells [29]. A Fhb KO showed decreased binding to vascular endothelial EA.hy926 cells [56]. Fhb contributes to *S. suis* survival in whole blood [53] and intracellular survival in PMN [53,55]. Fhb can bind factor H [52,53,55] and C3 simultaneously [53] and a Fhb KO showed decreased factor H binding and increased C3b/iC3b deposition [53,55]. Secreted Fhb lowers C3b/C3b deposition on a Fhb KO mutant and restores PMN intracellular survival of *S. suis*[53]. However, a Fhb KO in a different strain still bound factor H and degraded C3b, whilst THP-1 phagocytosis of the KO mutant was unaffected [29]. Translocation across and adhesion to hCMEC/D3 cells is decreased in a Fhb KO mutant [58] and factor H binding by Fhb increases adherence to BMEC cells [29]. Fhb was shown to bind fibrinogen [48].

#### Enolase

Enolase is a multifunctional protein with glycolytic functions and plasminogen binding abilities, and is found in many organisms [59]. In *S. suis*, enolase was found within the cytoplasm and on the cell surface of *S. suis*, although lacking a LPXTG-motif [60]. Blocking enolase functioning with recombinant protein or polyclonal antibodies was shown to decrease adherence to HEp2 cells [61–63]. Enolase was shown to bind fibronectin, [61] laminin [61] and factor H [52]. 40S ribosomal protein SA (RPSA), a protein involved in BBB integrity, was shown to increase at the cell surface of hCMEC/D3 cells when treated with enolase [64]. In transfected HEK-293 T cells it was demonstrated that enolase can interact with RPSA [64,65]. Enolase can induce apoptosis in HEK-293 T cells [65] and in hCMEC/D3 cells by interacting with RSPA [64,65]. The apoptosis induced by enolase is inhibited by caveolae, a type of lipid raft [64].

### Identification of putative zoonotic virulence factors

Out of 3307 *S. suis* BioSample records in the NCBI database, 315 human, 896 pig diseased and 492 pig healthy *S. suis* genome assemblies were included (appendix S1 p7, appendix S3), including human isolates from Vietnam (45%), China (31%), the Netherlands (9%), Thailand (5%) and Togo (5%). For 1012 (31%) BioSample records, the information on host was unavailable (appendix S1 p7).

**Figure 3.**
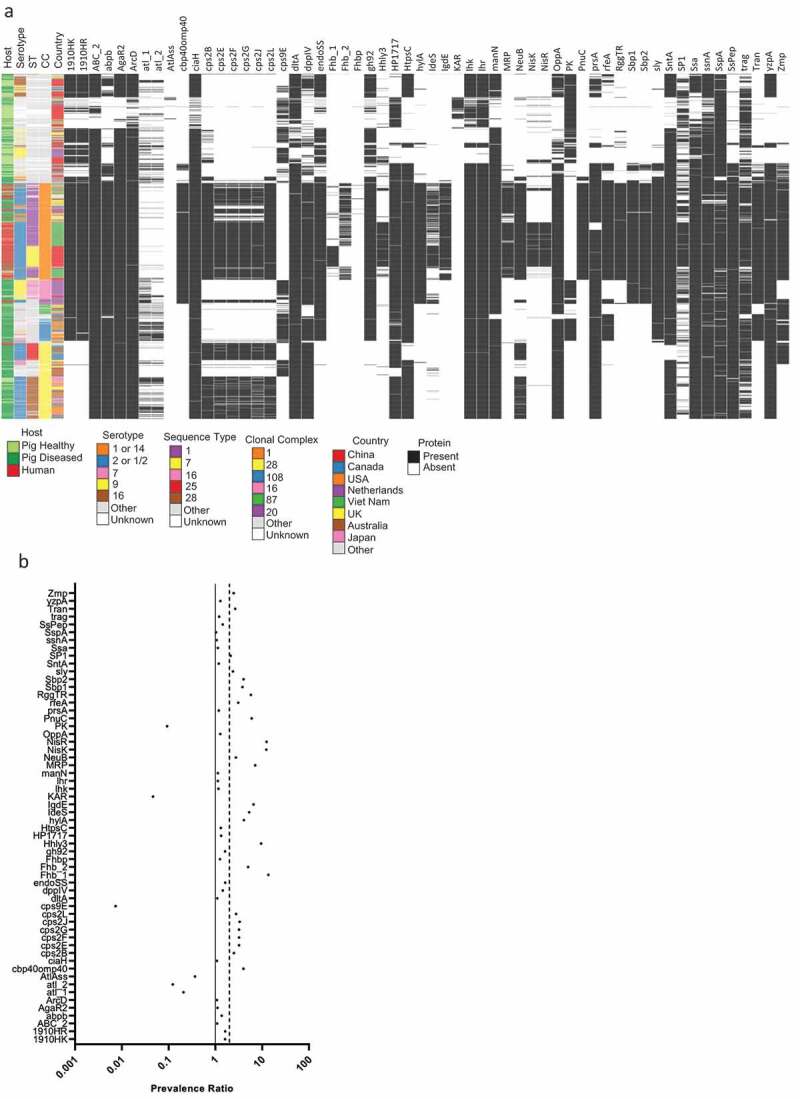
Presence of virulence factors in *S. suis* isolates and the corresponding virulence factor prevalence ratio in human isolates compared to pig isolates

Out of 111 unique protein sequences, including multiple alleles for four proteins, 53 proteins were encoded by genes which are part of the *S. suis* core genome (appendix S1 p8). The remaining 58 proteins were encoded by genes which are part of the accessory genome. The presence of these 58 accessory proteins together with isolate metadata was plotted against a clustered core genome alignment [11–14] (Figure 3a) and the human-pig prevalence ratio was calculated (Figure 3b, appendix 1 p9). Six proteins (Atl1, Atl2, AtlAss, CPS9E, KAR and PK) had a prevalence ratio below 1. For 26 proteins, including MRP, Sly and CPS2B/E/F/G/J/L which form a single operon [15], the prevalence ratio was above 2 and three of these proteins, Fhb_1, NisK and NisR were at least ten times more prevalent in human isolates than in pig isolates.

(A) The 1703 assemblies were clustered using IQ-TREE [11] based on a Roary [12] core gene alignment of a Prokka [13] annotated assembly. Presence of proteins in *S. Suis* isolates and isolate metadata were visualized in Phandango[14]. (B) Prevalence ratio of virulence factors in human isolates over pig isolates was based on virulence factor presence in *S. suis* genomes.

Ninety percent of human *S. suis* isolates had the same genetic background (CC1) while the pig isolates were genetically more diverse (Figure 3a). To adjust for potential lineage effects, we repeated our analysis for the 52 proteins with prevalence ratio above 1 in the first analysis, but restricted to CC1 isolates. Of these 52 proteins, 35 were encoded by genes which are part of the CC1 core genome, including Sly and CPS2B/L. Four proteins (nisin dependent two-component signal transduction system [NisK/R], putative hemolysin-III-related protein [Hhly3] and Fhb_1) had a prevalence ratio of at least 2 (appendix S1 p10-11). NisK/R and Hhly3 were initially discovered on the 89 K pathogenicity island found in Chinese human *S. suis* outbreak isolates belonging to ST7 [66,67]. Outside ST7 but within CC1, both PZVFs were also present in 110 Vietnamese human isolates from ST 1 (105), ST144 (3), ST869 (1) and ST951 (1), and in 4 Chinese human isolates from ST1 (2), ST665 (1) and ST658 (1).

Geographical clustering of *S. suis* lineages may explain the presence of NisK/R and Hhly3 in zoonotic isolates from certain countries. Therefore, we determined the prevalence ratio of these proteins per country of origin. The prevalence ratio within Chinese isolates was 6·4 for NisK/R and 5·5 for Hhly3. In addition, inclusion of multiple strains belonging to a single outbreak may cause confounding. When isolates from the Chinese outbreak in 2005 [68], which all except one harbored NisK/R and Hhly3, were excluded, the prevalence ratio within Chinese isolates was 3·7 for NisK/R and 3·1 for Hhly3. In Vietnamese isolates the prevalence ratio for NisK/R was 1·5 and for Hhly3 1·4. NisK/R and Hhly3 were not detected in human isolates from other countries than China and Vietnam.

## Discussion

We identified 124 *S. suis* putative virulence factors studied in a human model in our systematic review. In our subsequent genomic meta-analysis, we identified 26 putative virulence factors with prevalence at least two times higher in human isolates than in pig isolates, which were therefore considered as PZVFs.

The five virulence factors most studied in *in vitro* models of human origin were CPS, Sly, MRP, Fhb and enolase. The contribution of these five virulence factors to *S. suis* virulence has also been studied *in vivo* in pig and mouse infection models. In a review of studies of Sly, MRP and Fhb, these putative virulence factors were found not to be critical for virulence in all models [4]. CPS was shown to contribute to *S. suis* virulence *in vivo* in pig and mice [15,21,69,70,71]. Both Sly and MRP contributed to virulence in mice [50,51,28,72,73], but a Sly or MRP KO did not show decreased virulence in pigs [38,43,74]. Fhb was shown to contribute to virulence in pigs [55] and to be essential to cross the BBB via Gb3 in mice [58]. Enolase was only tested in mice and increased the BBB permeability [75]. Pig and mouse *in vivo* infection models appear to yield different outcomes for certain virulence factors. A similar observation was made for the difference in virulence of different *S. suis* serotype 2 strains, observed after experimental infections in pig and mouse [2]. These data indicate that, although we can learn much from these *in vivo* models, the translation of mice or pig infection studies to the human *S. suis* pathogenesis can be challenging.

In our genomic meta-analysis, proteins involved in the serotype 2 capsular polysaccharide biosynthesis were more prevalent in zoonotic isolates, confirming epidemiological observations [1]. Sly, MRP, and Fhb were identified as PZVF, while enolase was found to be part of the *S. suis* core genome and therefore not identified as PZVF. Only Fhb_1 remained more prevalent in human than in pig isolates within the CC1 lineage. In a previous genomic analysis, a comparison between human and pig isolates from Vietnam and pig isolates from the UK did not find a substantial enrichment of specific accessory genes in human isolates [76]. The prevalence of virulence factors was higher in clinical pig isolates than in non-clinical isolates from the UK [76]. Putative virulence factors were more abundant in Dutch zoonotic isolates than in non-zoonotic isolates [3]. Zoonotic and non-zoonotic strains could only be separated based on their accessory genome and not based on their core genome [3]. Moreover, zoonotic isolates with dissimilar core genomes showed similarity in their accessory genome [3], implying that PZVFs are most likely part of the accessory genome. Here, 53 of the putative virulence factors were encoded by genes which are part of the *S. suis* core genome. Given their function (appendix S2), many of these putative virulence factors are likely to be involved in *S. suis* metabolism although a role in pathogenesis cannot be ruled out. As was noted before and was also observed in this study, many *S. suis* putative virulence factors have not yet been thoroughly characterized [4]. Most virulence factors were studied in a single isolate instead of multiple isolates, introducing potential bias [77]. An additional concern is that isogenic KO mutants used to study the virulence factors were not always properly characterized. In 37% of the studies that used an isogenic KO mutant, the impact of the mutation on growth rate was not verified and therefore a direct effect of the KO on the experimental outcome due to changes in growth rate, instead of or in addition to a potential functional effect, cannot be ruled out.

Independent parallel genomic acquisition events can introduce different PZVFs that could drive the emergence of a zoonotic *S. suis* lineage, as observed in the Dutch zoonotic CC20 lineage [3]. Such acquisition event could explain why NisK/R or Hhly3 are not present in all human *S. suis* isolates. These findings suggest that these specific PZVFs are not essential for zoonotic potential per se, as the acquisition of other genes could confer zoonotic potential as well. However, within the zoonotic CC1 lineage or after stratification by country of origin, NisK/R and Hhly3, as well as Fhb_1 are still more prevalent in human isolates than in pig isolates suggesting that these PZVFs contribute to zoonotic potential.

Hhly3 is a cholesterol-independent hemolysin first discovered in the foodborne pathogen *Bacillus cereus* [78] and later also identified in the foodborne pathogen *Vibrio vulnificus*[79]. Hhly3 monomers bind in a temperature-dependent fashion to host cell membranes and form 3–3·5 mm pores after multimerization [80]. The cholesterol independency of Hhly3 could give *S. suis* the ability to induce pores in membranes with low cholesterol or unavailable cholesterol, such as endosomes [81]. The contribution of Hhly3 to *S. suis* virulence has not been studied in *in vivo* pig or mouse infection models yet.

Nisin is al antibiotic produced by several *Lactococcus* and *Streptococcus* species with antimicrobial properties against Gram-positive and Gram-negative bacteria [82]. Operons conferring nisin resistance in strains that cannot produce nisin themselves have mainly been found in human pathogenic strains, including *Streptococcus mutants* and *Streptococcus agalactiae*[82]. In *S. suis*, three independent acquisitions of nisin resistance genes have been reported. A complete nisin production and resistance locus including NisK/R was found on two different pathogenicity islands in two unrelated strains [83,84]. NisK/R was also present on the 89 K pathogenicity island in a CC1/ST7 strain from China [66]. Here we also detected NisK/R in CC1/ST1 strains from Vietnam. Besides conferring nisin resistance, NisK/R could potentially contribute to zoonotic potential by regulating gene expression [85]. NisK/R was demonstrated to contribute to *S. suis* virulence in mice [66]. A NisK/R KO mutant was shown to have decreased hemolytic activity and decreased adhesion to and invasion of HeLa cells [66].

Our study has several limitations. To determine the presence of the putative virulence factors in *S. suis* genomes, we mapped the proteins to the assembled genomes using a minimal identity of 95%. Although this cutoff can distinguish between virulent and avirulent MRP [50], it cannot distinguish small differences at the amino acid level. However, a single amino acid change can affect the function of a putative virulence factor, as for example recently shown for SadP [86]. Additionally, we included two articles in the systematic review that studied sRNAs [87,88], but our protein mapping approach did not permit meta-analysis of regulatory RNA molecules or regulatory non-coding DNA sequences that could contribute to virulence. Moreover, we determined the presence of single virulence factors and did not study a potential combined effect of virulence factors. For the proteins encoded by genes of the accessory genome we attempted to compare their prevalence in human and pig isolates per study, which would allow for a combined statistical analysis comparable to an individual patient data meta-analysis. However, only a single study systematically sampled both pig and human isolates [3], precluding such meta-analysis. Whilst we included all *S. suis* genomes with accompanying metadata present in NCBI BioSample, for 31% of BioSample records metadata were lacking, likely introducing further bias. We tried to overcome this limitation partly by performing our analysis within genomic lineage CC1 and for individual countries.

Genomic determinants associated with particular bacterial traits are increasingly identified using genome-wide association studies. Such studies require confirmation of biological relevance of genes with significant association. Here, we used a different approach by starting with a systematic approach toward identification of functional proteins and subsequent estimation of their relative frequency in genomes of strains representing different *S. suis* populations. The collected metadata with corresponding assembled genomes and the list of PZVF are valuable tools for further research into zoonotic potential of *S. suis*, the pathogenesis of zoonotic *S. suis* infections, and for early detection of emerging zoonotic lineages.

## Supplementary Material

Supplemental Material

## Data Availability

“The data that support the findings of this study are available in Zenodo at https://doi.org/10.5281/zenodo.4686597. These data were derived from the following resources available in the public domain: NCBI (https://www.ncbi.nlm.nih.gov/).”

## References

[1] Huong VTL, Ha N, Huy NT, *et al*. Epidemiology, clinical manifestations, and outcomes of Streptococcus suis infection in humans. Emerg Infect Dis J. 2014;20:1105. 10.3201/eid2007.131594 PMC4073838 24959701

[2] Vecht U, Stockhofe-Zurwieden N, Tetenburg BJ, et al. Virulence of Streptococcus suis type 2 for mice and pigs appeared host- specific. Vet Microbiol. 1997;58:53–60. 9451461 10.1016/s0378-1135(97)00131-4

[3] Willemse N, Howell KJ, Weinert LA, *et al*. An emerging zoonotic clone in the Netherlands provides clues to virulence and zoonotic potential of Streptococcus suis. Sci Rep. 2016;6:28984. 27381348 10.1038/srep28984 PMC4933891

[4] Segura M, Fittipaldi N, Calzas C, et al. Critical Streptococcus suis virulence factors: are they all really critical? Trends Microbiol. 2017;25:585–599. 28274524 10.1016/j.tim.2017.02.005

[5] Fulde M, Valentin-Weigand P. Epidemiology and pathogenicity of zoonotic streptococci. Curr Top Microbiol Immunol. 2013;368:49-81. DOI: 10.1007/82_2012_277.PMID:23192319 10.1007/82_2012_277 23192319

[6] Arenas J, Zomer A, Harders-Westerveen J, *et al*. Identification of conditionally essential genes for Streptococcus suis infection in pigs. Virulence. 2020;11:446–464. 32419603 10.1080/21505594.2020.1764173 PMC7239030

[7] Saralahti, A. and Rämet, M. Zebrafish and Streptococcal Infections.Scand J Immunol. 2015;82:174–183. 26095827 10.1111/sji.12320

[8] Moher D, Shamseer L, Clarke M, *et al*. Preferred reporting items for systematic review and meta-analysis protocols (PRISMA-P) 2015 statement. Syst Rev. 2015;4:1. 25554246 10.1186/2046-4053-4-1 PMC4320440

[9] Koster J. Pubmed Pubreminer. AMC, UvA; 2004. http://hgserver2.amc.nl/cgi-bin/miner/miner2.cgi

[10] Jolley KA, Bray JE, Maiden MCJ. Open-access bacterial population genomics: bIGSdb software, the PubMLST.org website and their applications. Wellcome Open Res. 2018;3:124. 30345391 10.12688/wellcomeopenres.14826.1 PMC6192448

[11] Minh BQ, Schmidt HA, Chernomor O, *et al*. IQ-TREE 2: new models and efficient methods for phylogenetic inference in the genomic Era. Mol Biol Evol. 2020;37:1530–1534. 32011700 10.1093/molbev/msaa015 PMC7182206

[12] Page AJ, Cummins CA, Hunt M, *et al*. Roary: rapid large-scale prokaryote pan genome analysis. Bioinformatics. 2015;31:3691–3693. 26198102 10.1093/bioinformatics/btv421 PMC4817141

[13] Seemann T. Prokka: rapid prokaryotic genome annotation. Bioinformatics. 2014;30:2068–2069. 24642063 10.1093/bioinformatics/btu153

[14] Hadfield J, Croucher NJ, Goater RJ, et al. Phandango: an interactive viewer for bacterial population genomics. Bioinformatics. 2017;34:292–293. 10.1093/bioinformatics/btx610 PMC5860215 29028899

[15] Smith HE, Damman M, van der Velde J, *et al*. Identification and characterization of the cps locus of Streptococcus suis serotype 2: the capsule protects against phagocytosis and is an important virulence factor. Infect Immun. 1999;67:1750–1756. 10085014 10.1128/iai.67.4.1750-1756.1999 PMC96524

[16] Okura M, Takamatsu D, Maruyama F, *et al*. Genetic analysis of capsular polysaccharide synthesis gene clusters from all serotypes of Streptococcus suis: potential mechanisms for generation of capsular variation. Appl Environ Microbiol. 2013;79:2796–2806. 23416996 10.1128/AEM.03742-12 PMC3623174

[17] Lalonde M, Segura M, Lacouture S, et al. Interactions between Streptococcus suis serotype 2 and different epithelial cell lines. Microbiology-Sgm. 2000;146:1913–1921. 10.1099/00221287-146-8-1913 10931895

[18] Zhang Y, Ding D, Liu M, *et al*. Effect of the glycosyltransferases on the capsular polysaccharide synthesis of Streptococcus suis serotype 2. Microbiol Res. 2016;185:45–54. 26946377 10.1016/j.micres.2016.02.002

[19] Benga L, Goethe R, Rohde M, et al. Non-encapsulated strains reveal novel insights in invasion and survival of Streptococcus suis in epithelial cells. Cell Microbiol. 2004;6:867–881. 15272867 10.1111/j.1462-5822.2004.00409.x

[20] Ferrando ML, De Greeff A, van Rooijen WJ, *et al*. Host-pathogen Interaction at the intestinal mucosa correlates with zoonotic potential of Streptococcus suis. J Infect Dis. 2015;212:95–105. 25525050 10.1093/infdis/jiu813 PMC4462715

[21] Feng Y, Cao M, Shi J, *et al*. Attenuation of Streptococcus suis virulence by the alteration of bacterial surface architecture. Sci Rep. 2012;2:710. 23050094 10.1038/srep00710 PMC3464449

[22] Salasia SI, Lammler C, Herrmann G. Properties of a Streptococcus suis isolate of serotype 2 and two capsular mutants. Vet Microbiol. 1995;45:151–156. 7571366 10.1016/0378-1135(95)00036-a

[23] Huang W, Chen Y, Li Q, *et al*. LytR plays a role in normal septum formation and contributes to full virulence in Streptococcus suis. Vet Microbiol. 2021;254:109003. 10.1016/j.vetmic.2021.109003 33561639

[24] Esgleas M, Lacouture S, Gottschalk M. Streptococcus suis serotype 2 binding to extracellular matrix proteins. FEMS Microbiol Lett. 2005;244:33–40. 15727818 10.1016/j.femsle.2005.01.017

[25] Jobin MC, Gottschalk M, Grenier D. Upregulation of prostaglandin E2 and matrix metalloproteinase 9 production by human macrophage-like cells: synergistic effect of capsular material and cell wall from Streptococcus suis. Microb Pathog. 2006;40:29–34. 16324819 10.1016/j.micpath.2005.10.003

[26] Meijerink M, Ferrando ML, Lammers G, et al. Immunomodulatory effects of Streptococcus suis capsule type on human dendritic cell responses, phagocytosis and intracellular survival. PLoS One. 2012;7:e35849. 22558240 10.1371/journal.pone.0035849 PMC3338795

[27] Zaccaria E, Cao R, Wells JM, et al. Model to assess virulence of porcine Streptococcus suis strains. PLoS One. 2016;11:e0151623. 26999052 10.1371/journal.pone.0151623 PMC4801416

[28] Lin L, Xu L, Lv W, *et al*. An NLRP3 inflammasome-triggered cytokine storm contributes to streptococcal toxic shock-like syndrome (STSLS). PLoS Pathog. 2019;15:e1007795. 10.1371/journal.ppat.1007795 PMC6553798 31170267

[29] Roy D, Grenier D, Segura M, et al. Recruitment of factor H to the Streptococcus suis cell surface is multifactorial. Pathogens. 2016;5. DOI:10.3390/pathogens5030047. PMC5039427 27399785

[30] Graveline R, Segura M, Radzioch D, et al. TLR2-dependent recognition of Streptococcus suis is modulated by the presence of capsular polysaccharide which modifies macrophage responsiveness. Int Immunol. 2007;19:375–389. 17307800 10.1093/intimm/dxm003

[31] Auger JP, Christodoulides M, Segura M, et al. Interactions of Streptococcus suis serotype 2 with human meningeal cells and astrocytes. BMC Res Notes. 2015;8:607. 26502903 10.1186/s13104-015-1581-2 PMC4624383

[32] Schwerk C, Papandreou T, Schuhmann D, *et al*. Polar invasion and translocation of neisseria meningitidis and Streptococcus suis in a novel human model of the blood-cerebrospinal fluid barrier. PLoS One. 2012;7:e30069. 22253884 10.1371/journal.pone.0030069 PMC3256222

[33] Vadeboncoeur N, Segura M, Al-Numani D, et al. Pro-inflammatory cytokine and chemokine release by human brain microvascular endothelial cells stimulated by Streptococcus suis serotype 2. FEMS Immunol Med Microbiol. 2003;35:49–58. 12589957 10.1111/j.1574-695X.2003.tb00648.x

[34] Jacobs AA, Loeffen PL, Van Den Berg AJ, et al. Identification, purification, and characterization of a thiol-activated hemolysin (suilysin) of Streptococcus suis. Infect Immun. 1994;62:1742–1748. 8168935 10.1093/benz/9780199773787.article.b00034458 PMC186398

[35] Leung C, N V D, Lukoyanova N, *et al*. Stepwise visualization of membrane pore formation by suilysin, a bacterial cholesterol-dependent cytolysin. Elife. 2014;3:e04247. 25457051 10.7554/eLife.04247 PMC4381977

[36] Norton PM, Rolph C, Ward PN, et al. Epithelial invasion and cell lysis by virulent strains of Streptococcus suis is enhanced by the presence of suilysin. FEMS Immunol Med Microbiol. 1999;26:25–35. 10518040 10.1111/j.1574-695X.1999.tb01369.x

[37] Seitz M, Baums CG, Neis C, *et al*. Subcytolytic effects of suilysin on interaction of Streptococcus suis with epithelial cells. Vet Microbiol. 2013;167:584–591. 24095145 10.1016/j.vetmic.2013.09.010

[38] Lun S, Perez-Casal J, Connor W, et al. Role of suilysin in pathogenesis of Streptococcus suis capsular serotype 2. Microb Pathog. 2003;34:27–37. 12620382 10.1016/s0882-4010(02)00192-4

[39] Chen S, Xie W, Wu K, *et al*. Suilysin stimulates the release of heparin binding protein from neutrophils and increases vascular permeability in mice. Front Microbiol. 2016;7:1338. 27617009 10.3389/fmicb.2016.01338 PMC4999480

[40] Zhang S, Zheng Y, Chen S, *et al*. Suilysin-induced platelet-neutrophil complexes formation is triggered by pore formation-dependent calcium influx. Sci Rep. 2016;6:36787. 27830834 10.1038/srep36787 PMC5103290

[41] Jobin MC, Fortin J, Willson PJ, et al. Acquisition of plasmin activity and induction of arachidonic acid release by Streptococcus suis in contact with human brain microvascular endothelial cells. FEMS Microbiol Lett. 2005;252:105–111. 16182470 10.1016/j.femsle.2005.08.044

[42] Vecht U, Wisselink HJ, Jellema ML, et al. Identification of two proteins associated with virulence of Streptococcus suis type 2. Infect Immun. 1991;59:3156–3162. 1879937 10.1128/iai.59.9.3156-3162.1991 PMC258147

[43] Smith HE, Vecht U, Wisselink HJ, et al. Mutants of Streptococcus suis types 1 and 2 impaired in expression of muramidase-released protein and extracellular protein induce disease in newborn germfree pigs. Infect Immun. 1996;64:4409–4412. 8926123 10.1128/iai.64.10.4409-4412.1996 PMC174391

[44] Baums CG, Valentin-Weigand P. Surface-associated and secreted factors of Streptococcus suis in epidemiology, pathogenesis and vaccine development. Anim Heal Res Rev. 2009;10:65. 10.1017/S146625230999003X 19558750

[45] Smith HE, Vecht U, Gielkens AL, et al. Cloning and nucleotide sequence of the gene encoding the 136-kilodalton surface protein (muramidase-released protein) of Streptococcus suis type 2. Infect Immun. 1992;60:2361–2367. 1587602 10.1128/iai.60.6.2361-2367.1992 PMC257166

[46] Zhang W, Liu G, Tang F, *et al*. Pre-absorbed immunoproteomics: a novel method for the detection of Streptococcus suis surface proteins. PLoS One. 2011;6:e21234. 21713002 10.1371/journal.pone.0021234 PMC3119691

[47] Rui L, Weiyi L, Yu M, *et al*. The serine/threonine protein kinase of Streptococcus suis serotype 2 affects the ability of the pathogen to penetrate the blood-brain barrier. Cell Microbiol. 2018;20:e12862. 29797543 10.1111/cmi.12862

[48] Pian Y, Wang P, Liu P, *et al*. Proteomics identification of novel fibrinogen-binding proteins of Streptococcus suis contributing to antiphagocytosis. Front Cell Infect Microbiol. 2015;5:19. 25789245 10.3389/fcimb.2015.00019 PMC4349166

[49] Pian Y, Li X, Zheng Y, et al. Binding of human fibrinogen to MRP enhances Streptococcus suis survival in host blood in a alphaXbeta2 Integrin-dependent manner. Sci Rep. 2016;6:26966. 27231021 10.1038/srep26966 PMC4882601

[50] Li Q, Fu Y, Ma C, *et al*. The non-conserved region of MRP is involved in the virulence of Streptococcus suis serotype 2. Virulence. 2017;8:1274–1289. 28362221 10.1080/21505594.2017.1313373 PMC5711419

[51] Wang J, Kong D, Zhang S, *et al*. Interaction of fibrinogen and muramidase-released protein promotes the development of Streptococcus suis meningitis. Front Microbiol. 2015;6:1001. 26441928 10.3389/fmicb.2015.01001 PMC4585153

[52] Li Q, Ma C, Fu Y, *et al*. Factor H specifically capture novel Factor H-binding proteins of Streptococcus suis and contribute to the virulence of the bacteria. Microbiol Res. 2017;196:17–25. 28164787 10.1016/j.micres.2016.11.011

[53] Li X, Liu P, Gan S, *et al*. Mechanisms of host-pathogen protein complex formation and bacterial immune evasion of Streptococcus suis protein Fhb. J Biol Chem. 2016;291:17122–17132. 27342778 10.1074/jbc.M116.719443 PMC5016116

[54] Kouki A, Haataja S, Loimaranta V, et al. Identification of a novel streptococcal adhesin P (SadP) protein recognizing galactosyl-α1-4-galactose-containing glycoconjugates: convergent evolution of bacterial pathogens to binding of the same host receptor. J Biol Chem. 2011;286:38854–38864. 21908601 10.1074/jbc.M111.260992 PMC3234710

[55] Pian Y, Gan S, Wang S, *et al*. Fhb, a novel factor H-binding surface protein, contributes to the antiphagocytic ability and virulence of Streptococcus suis. Infect Immun. 2012;80:2402–2413. 22526676 10.1128/IAI.06294-11 PMC3416472

[56] Madar Johansson M, Bélurier E, Papageorgiou AC, *et al*. The binding mechanism of the virulence factor Streptococcus suis adhesin P subtype to Globotetraosylceramide is associated with systemic disease. J Biol Chem. 2020;295:14305–14324. 32796033 10.1074/jbc.RA120.014818 PMC7573278

[57] Ferrando ML, Willemse N, Zaccaria E, et al. Streptococcal adhesin P (SadP) contributes to Streptococcus suis adhesion to the human intestinal epithelium. PLoS One. 2017;12:e0175639. 28407026 10.1371/journal.pone.0175639 PMC5391093

[58] Kong D, Chen Z, Wang J, *et al*. Interaction of factor H-binding protein of Streptococcus suis with globotriaosylceramide promotes the development of meningitis. Virulence. 2017;8:1290–1302. 28402705 10.1080/21505594.2017.1317426 PMC5711355

[59] À D-R, Roig-Borrellas A, García-Melero A, et al. α-Enolase, a multifunctional protein: its role on pathophysiological situations. J Biomed Biotechnol. 2012;2012:156795. doi: 10.1155/2012/156795 PMC3479624 23118496

[60] Esgleas M, Li Y, Hancock MA, et al. Isolation and characterization of alpha-enolase, a novel fibronectin-binding protein from Streptococcus suis. Microbiology. 2008;154:2668–2679. 18757800 10.1099/mic.0.2008/017145-0

[61] Li Q, Liu H, Du D, *et al*. Identification of novel laminin- and fibronectin-binding proteins by far-western blot: capturing the adhesins of Streptococcus suis type 2. Front Cell Infect Microbiol. 2015;5:82. 26636044 10.3389/fcimb.2015.00082 PMC4644805

[62] Feng Y, Pan X, Sun W, *et al*. Streptococcus suis enolase functions as a protective antigen displayed on the bacterial cell surface. J Infect Dis. 2009;200:1583–1592. 19848587 10.1086/644602

[63] Chen B, Zhang A, Xu Z, et al. Large-scale identification of bacteria-host crosstalk by affinity chromatography: capturing the interactions of Streptococcus suis proteins with host cells. J Proteome Res. 2011;10:5163–5174. 21942651 10.1021/pr200758q

[64] Jiang H, Wu T, Liu J, *et al*. Caveolae/rafts protect human cerebral microvascular endothelial cells from Streptococcus suis serotype 2 α-enolase-mediated injury. Vet Microbiol. 2021;254:108981. 10.1016/j.vetmic.2021.108981 33445055

[65] Liu H, Lei S, Jia L, *et al*. Streptococcus suis serotype 2 enolase interaction with host brain microvascular endothelial cells and RPSA-induced apoptosis lead to loss of BBB integrity. Vet Res. 2021;52. DOI:10.1186/s13567-020-00887-6. PMC7898445 33618766

[66] Xu J, Fu S, Liu M, *et al*. The two-component system NisK/NisR contributes to the virulence of Streptococcus suis serotype 2. Microbiol Res. 2014;169:541–546. 24342108 10.1016/j.micres.2013.11.002

[67] Zheng JX, Li Y, Zhang H, et al. Identification and characterization of a novel hemolysis-related gene in Streptococcus suis serotype 2. PLoS One. 2013;8:e74674. 24069329 10.1371/journal.pone.0074674 PMC3775796

[68] Du P, Zheng H, Zhou J, *et al*. Detection of multiple parallel transmission outbreak of Streptococcus suis human infection by use of genome epidemiology, China, 2005. Emerg Infect Dis. 2017;23:204–211. 27997331 10.3201/eid2302.160297 PMC5324821

[69] Charland N, Harel J, Kobisch M, et al. Streptococcus suis serotype 2 mutants deficient in capsular expression. Microbiology. 1998;144(Pt 2):325–332. 9493370 10.1099/00221287-144-2-325

[70] Okura M, Auger J-P, Shibahara T, *et al*. Capsular polysaccharide switching in Streptococcus suis modulates host cell interactions and virulence. Sci Rep. 2021;11. DOI:10.1038/s41598-021-85882-3. PMC7985379 33753801

[71] Lin L, Xu L, Lv W, *et al*. An NLRP3 inflammasome-triggered cytokine storm contributes to streptococcal toxic shock-like syndrome (STSLS). PLoS Pathog. 2019;15:e1007795. 10.1371/journal.ppat.1007795 PMC6553798 31170267

[72] He Z, Pian Y, Ren Z, *et al*. Increased production of suilysin contributes to invasive infection of the Streptococcus suis strain 05ZYH33. Mol Med Rep. 2014;10:2819–2826. 25241621 10.3892/mmr.2014.2586 PMC4227431

[73] Takeuchi D, Akeda Y, Nakayama T, *et al*. The contribution of suilysin to the pathogenesis of Streptococcus suis meningitis. J Infect Dis. 2014;209:1509–1519. 24285845 10.1093/infdis/jit661

[74] Allen AG, Bolitho S, Lindsay H, *et al*. Generation and characterization of a defined mutant of Streptococcus suis lacking suilysin. Infect Immun. 2001;69:2732–2735. 11254643 10.1128/IAI.69.4.2732-2735.2001 PMC98215

[75] Sun Y, Li N, Zhang J, *et al*. Enolase of Streptococcus suis serotype 2 enhances blood-brain barrier permeability by inducing IL-8 release. Inflammation. 2016;39:718–726. 26732390 10.1007/s10753-015-0298-7

[76] Weinert LA, Chaudhuri RR, Wang J, *et al*. Genomic signatures of human and animal disease in the zoonotic pathogen Streptococcus suis. Nat Commun. 2015;6:6740. 25824154 10.1038/ncomms7740 PMC4389249

[77] Auger JP, Chuzeville S, Roy D, *et al*. The bias of experimental design, including strain background, in the determination of critical Streptococcus suis serotype 2 virulence factors. PLoS One. 2017;12:e0181920. 28753679 10.1371/journal.pone.0181920 PMC5533308

[78] Baida GE, Kuzmin NP. Cloning and primary structure of a new hemolysin gene from Bacillus cereus. Biochim Biophys Acta. 1995;1264:151–154. 7495855 10.1016/0167-4781(95)00150-f

[79] Chen Y-C, Chang M-C, Chuang Y-C, et al. Characterization and virulence of hemolysin III from vibrio vulnificus. Curr Microbiol. 2004;49:175–179. 15386100 10.1007/s00284-004-4288-5

[80] Baida GE, Kuzmin NP. Mechanism of action of hemolysin III from Bacillus cereus. Biochim Biophys Acta (BBA)-Biomembranes. 1996;1284:122–124. 8962879 10.1016/s0005-2736(96)00168-x

[81] Bieberich E. Sphingolipids and lipid rafts: novel concepts and methods of analysis. Chem Phys Lipids. 2018;216:114–131. 30194926 10.1016/j.chemphyslip.2018.08.003 PMC6196108

[82] Khosa S, Lagedroste M, Smits SHJ. Protein defense systems against the lantibiotic nisin: function of the immunity protein NisI and the resistance protein NSR. Front Microbiol. 2016;7:504. 27148193 10.3389/fmicb.2016.00504 PMC4828448

[83] Wu Z, Wang W, Tang M, *et al*. Comparative genomic analysis shows that Streptococcus suis meningitis isolate SC070731 contains a unique 105K genomic island. Gene. 2014;535:156–164. 24316490 10.1016/j.gene.2013.11.044

[84] Zhu Y, Zhang Y, Ma J, *et al*. ICESsuHN105, a novel multiple antibiotic resistant ICE in Streptococcus suis serotype 5 strain HN105. Front Microbiol. 2019;10:274. 30863372 10.3389/fmicb.2019.00274 PMC6399138

[85] Kawada-Matsuo M, Watanabe A, Arii K, *et al*. Staphylococcus aureus virulence affected by an alternative Nisin A resistance mechanism. Appl Environ Microbiol. 2020Apr 1;86(8):e02923-19. doi: 10.1128/AEM.02923-19. PMID: 32086306. PMC7117933 32086306

[86] Johansson MM, Bélurier E, Papageorgiou AC, *et al*. The binding mechanism of the virulence factor Streptococcus suis adhesin P subtype to globotetraosylceramide is associated with systemic disease. J Biol Chem. 2020;295:14305–14324. 32796033 10.1074/jbc.RA120.014818 PMC7573278

[87] Wu Z, Wu C, Shao J, *et al*. The Streptococcus suis transcriptional landscape reveals adaptation mechanisms in pig blood and cerebrospinal fluid. RNA. 2014;20:882–898. 24759092 10.1261/rna.041822.113 PMC4024642

[88] Gong X, Zhuge Y, Ding C, *et al*. A novel small RNA contributes to restrain cellular chain length and anti-phagocytic ability in Streptococcus suis 2. Microb Pathog. 2019;137:103730. 31499182 10.1016/j.micpath.2019.103730

