# Supplementary material for: Identification of Streptococcus suis putative zoonotic virulence factors: A systematic review and genomic meta-analysis
Source: Virulence. 2021 Nov 25;12(1):2787–97. doi: 10.1080/21505594.2021.1985760 (PMC8632099; doi:10.1080/21505594.2021.1985760)
Supplement: Supplemental Material [file KVIR_A_1985760_SM7428.zip › supplementary/2021.09.22_Appendix1 (1).docx]

**Appendix**

**Search queries**

**PubMed**"STREPTOCOCCUS SUIS" AND (VIRULENCE OR VIRULENT OR ZOONOTIC OR HOST-PATHOGEN OR BACTERIA-HOST OR “HOST CELLS”) AND ((ADHERE OR ADHERED OR ADHERES) OR (ADHERENT OR ADHERENCE) OR (ADHESIN OR ADHESINS) OR (IMMUNE OR IMMUNITY OR MACROPHAGES) OR (INTRACELLULAR OR INTRACELLULARLY) OR (INVADE OR INVADES) OR (INVAS OR INVASION) OR (TRANSLOCATE OR TRANSLOCATED OR TRANSLOCATION) OR ADHESION OR ADHESIVE OR BLOOD OR IMMUNOLOGY OR INVASIVE OR INVASIVENESS OR ZEBRAFISH OR MENINGITIS OR CSF OR SURVIV*) NOT (CASE REPORTS OR LETTER OR REVIEW)

**Scopus**TITLE-ABS-KEY ( "STREPTOCOCCUS SUIS" AND (VIRULENCE OR VIRULENT OR ZOONOTIC OR HOST-PATHOGEN OR BACTERIA-HOST OR “HOST CELLS”) AND ((ADHERE OR ADHERED OR ADHERES) OR (ADHERENT OR ADHERENCE) OR (ADHESIN OR ADHESINS) OR (IMMUNE OR IMMUNITY OR MACROPHAGES) OR (INTRACELLULAR OR INTRACELLULARLY) OR (INVADE OR INVADES) OR (INVAS OR INVASION) OR (TRANSLOCATE OR TRANSLOCATED OR TRANSLOCATION) OR ADHESION OR ADHESIVE OR BLOOD OR IMMUNOLOGY OR INVASIVE OR INVASIVENESS OR ZEBRAFISH OR MENINGITIS OR CSF OR SURVIV*)) AND ( LIMIT-TO ( DOCTYPE , "ar" ) )

**Table 1 Data extracted from included articles supplemented with core or accessory genome assignment of putative virulence factors in the genomic meta-analysis.** Abbreviations: BBB: Blood Brain Barrier, Rec: Recombinant, Pur: Purified, KO: Knockout, NA: Not Applicable, ND: Not Determined. (Appendix 2)

**Bacterial genome and metadata acquisition**

We downloaded BioSample records from NCBI mentioning “*Streptococcus suis”*. Missing metadata were searched in the BioSample record corresponding publications and pubMLST^1^, and added. Data not found were marked as unknown. Genomes were included if at least metadata on host, host health status, and country of origin were available. BioSample records were assigned to one of three groups according to the host origin of the isolate: human, diseased pig and healthy pig according to BioSample record keywords (appendix 1 p3, see below). Whole genome sequences linked to the BioSample records were subsequently downloaded. Raw Illumina sequencing data were assembled using SKESA v2.1.0.^2^ If raw Illumina data were not available, Genbank assemblies were included. Complete genome assemblies were preferred over SKESA reassemblies. Assembly quality was assessed with Quast v5.0.2 and the results were summarized using MultiQC v1.6.^3,4^ Any assembly with more than 500 contigs, a N50 lower than 10 kbp, a genome size outside 1.6-3.0 Mbp, a GC content outside 40.0-42.5% or more than 50 Ns/100 kbp was excluded. Average nucleotide identity (ANI) values were calculated between all genomes using fastANI v1.1.^5^ A network was constructed only retaining edges with ANI values above 95%, using network_analysis_scripts^6^ and visualized in Cytoscape v3.7.1.^7^ Genomes outside the main discrete cluster of *S. suis* genomes were excluded. Serotype was determined using the reference alleles from the SsuiSerotyping pipeline in ABRicate^8,9^ and supplemented with available BioSample record metadata. Clonal complex was assigned based on sequence type (ST) inferred using mlst and the PubMLST typing schemes.^1^ The curated set of assembled genomes with corresponding metadata was deposited on Zenodo (https://doi.org/10.5281/zenodo.4686597).

**Genome meta-analysis**Protein sequences of PZVF were downloaded from the NCBI database using respective NCBI protein ID. Duplicates were removed by protein clustering using CD-HIT v4.7 with default settings.^10^ Presence of PZVF protein sequences was determined in translated genome assemblies using tblastn implemented in ABRicate with a minimal protein identity of 95% and coverage of 60% without a culling limit^8^ and visualized together with metadata in phandango.^11^

**Keywords for BioSample record categorization**

**Human**
Host = Human or Homo sapiens

**Pig Diseased**
Host = Pig OR swine OR Sus OR Sus crofa OR Sus crofa domestica OR Sus crofa domesticus
Health= Diseased, Meningitis, Arthritis, Septicemia, Virulent, Pneumonia, Systemic, Clinical (isolate/disease), Endocarditis, Polyserositis, *S. suis* Infection, streptococcosis and Respiratory with in the lung presence of gross lesions of pneumonia

**Pig Healthy**Host = Pig OR swine OR Sus OR Sus crofa OR Sus crofa domestica OR Sus crofa domesticus
Health=Clinically healthy, healthy, Carrier, Non-clinical, Non-pathogenic, Avirulent, Host disease no and (Clinically) asymptomatic

**Table 2 Summary of experimental outcomes per model for the five most studied virulence factors.** For each of the five virulence factors the analysis approach, which in vitro models were used, and the experimental outcomes were recorded.

| **Factor** | **Studied as** | ***In vitro* model** | **Experimental Outcome** |
| --- | --- | --- | --- |
| Capsular Polysaccharide | KO | HEp2 | Increased adherence and invasion.^12–14^ |
|  |  | Caco-2 | Increased adherence, invasion and translocation.^15^ |
|  |  | THP-1 | Increased phagocytosis,^16^ inflammasome activation^17^ and secretion of TNF and IL1 but decreased MCP-1 secretion.^18^ |
|  |  | HeLa | Adhesion was unaffected.^19^  Adhesion was increased.^20^ |
|  |  | PMN | Increased phagocytosis^19^ and decreased intracellular survival.^21^ |
|  |  | DC | Increased phagocytosis and unaffected intracellular survival.^22^ |
|  |  | Zebrafish larvae | Decreased zebrafish survival.^23^ |
|  |  | BMEC | Increased secretion of IL6 and IL8 but decreased MCP-1 secretion.^24^ |
|  |  | HIBCCP | Trend in increased translocation.^25^ |
|  |  | Meningeal cells | Increased adherence and invasion.^26^ |
|  |  | Fetal Astrocytes |  |
|  |  | Buccal Mucosa Epithelial cells | Increased adherence.^19^ |
|  |  | HUVEC | Increased adherence.^13^ |
|  |  | A549 | Increased adherence.^20^ |
|  |  | Fibrinogen | Increased adherence.^27^ |
|  |  | Blood | Decreased survival.^21^ |
|  | Purified CPS | U937 | Induced PGE2 and MMP-9 secretion^28^ |
|  |  | Factor H | Binds factor H ^16^ |
| Suilysin | Antibody | HEp2 | Anti-Sly antibody decreases HEp2 lysis.^29^ |
|  | KO |  | Decreased invasion but unaffected adherence.^30^ |
|  |  | Caco-2 | Translocation unaffected.^15^ |
|  |  | THP-1 | Decreased inflammasome activation.^17^ |
|  | Recombinant Protein | Blood | Induces p-selectin mediated platelet neutrophil complex formation.^31^ |
|  | KO |  | Decreased heparin binding protein release.^32^ |
|  |  | PMN | Decreased heparin binding protein release and PMN degranulation.^32^ |
|  | Recombinant Protein | Monocytes | Increased TNF secretion.^33^ |
|  | KO | BMEC | Decreased arachidonic acid release.^34^ |
|  |  | Meningeal cells | Adherence and invasion were unaffected.^26^ |
|  |  | Fetal Astrocytes | Adherence was unaffected, invasion was decreased.^26^ |
| Muramidase-released protein | KO | Blood | Decreased survival.^35,36^ |
|  |  | PMN | Decreased survival.^35,36^ |
|  |  | Serum | Unaffected PMN killing.^36^ |
|  |  | Plasma | Decreased resistance to PMN killing.^36^ |
|  |  | HEp2 | Decreased adherence.^37^ |
|  | Recombinant Protein |  | Bound.^38^ |
|  |  | Factor H | Bound.^37,39^ |
|  |  | Fibronectin | Bound.^37^ |
|  |  | Fibrinogen | Bound.^37^ |
|  | KO |  | Decreased binding.^35,36^ |
|  |  |  | Dose dependent decreased resistance to PMN killing.^36^ |
|  |  | hCMEC/D3 with fibrinogen | Decreased adhesion, translocation and p120-catenin.^40^ |
| Factor H binding protein/  Streptococcal adhesin P | Recombinant Protein | Factor H | Dose dependent binding.^39^ |
|  | KO |  |  |
|  |  |  | Deposition unaffected.^16^  Decreased deposition.^41,42^ |
|  |  | C3b/iC3b | Increased deposition,^41,42^ |
|  | Extracted Protein | C3b and C3d | Dose dependent binding.^42^ |
|  | KO | C3b | Cleavage of C3b is not affected.^16^ |
|  |  | PMN | Decreased survival in PMN.^41,42^ |
|  |  | Blood | Decreased survival.^41^ |
|  |  | hCMEC/D3 | Decreased adhesion and translocation.^43^ |
|  |  | Caco-2 | Decreased adhesion and translocation.^44^ |
|  |  | A549 | Adhesion not affected.^16^ |
|  |  | HBMEC | Adhesion not affected.^16^ |
|  |  | Macrophages | Phagocytosis not affected.^16^ |
|  |  | Fibrinogen | Dose dependent binding.^35^ |
|  |  | EA.hy926 | Decreased adhesion.^45^ |
|  | Recombinant Protein | Gb3 | Strong binding by SadP_n_ and SadP_o_.^45^ |
|  |  | Gb4 | Occasional binding by SadP_n_ and no binding by SadP_o_.^45^ |
| Enolase | Recombinant Protein | Laminin | Dose dependent binding.^46^ |
|  |  | Fibronectin | Dose dependent binding.^46^ |
|  |  | Factor H | Dose dependent binding and blocking enolase decreased factor H binding by *S. suis*.^39^ |
|  |  | hCMEC/D3 | Eno increases RPSA at cell surface.^47^  Eno induces cell death.^47,48^  Eno induced apoptosis is prevented by caveolae/rafts.^47^ |
|  |  | HEK293T RSPA transfected | Eno interacts with RSPA.^47,48^  Eno induces apoptosis.^48^ |
|  |  | HEp2 | Recombinant and purified protein decreased adhesion.^46,49,50^ |
|  | Purified Protein |  |  |

**Figure 1 Flow chart of inclusion of S. suis genomes from NCBI BioSample records.** Included BioSample records with collected metadata is provided in appendix 3.

**Table 3 The 53 putative virulence factors encoded by genes of the *S. suis* core genome.
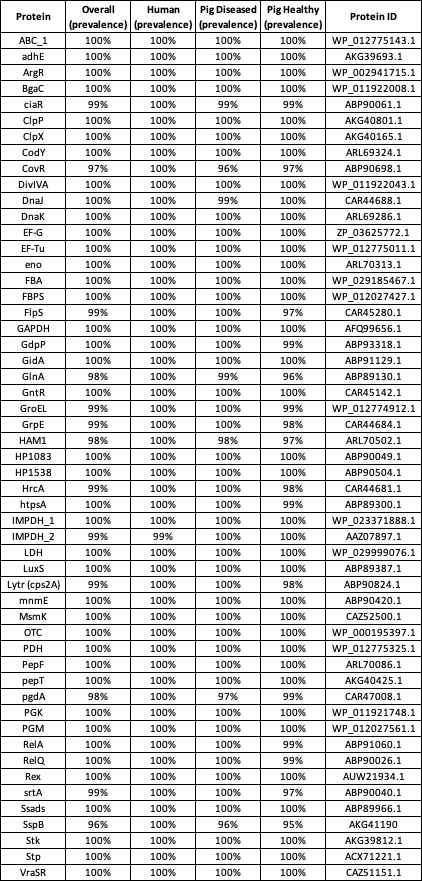
**

**Table 4 Prevalence of proteins encoded by genes of the accessory genome and their Human-Pig prevalence ratio. Proteins with a prevalence ratio above 2 were identified as PZVF.**

***
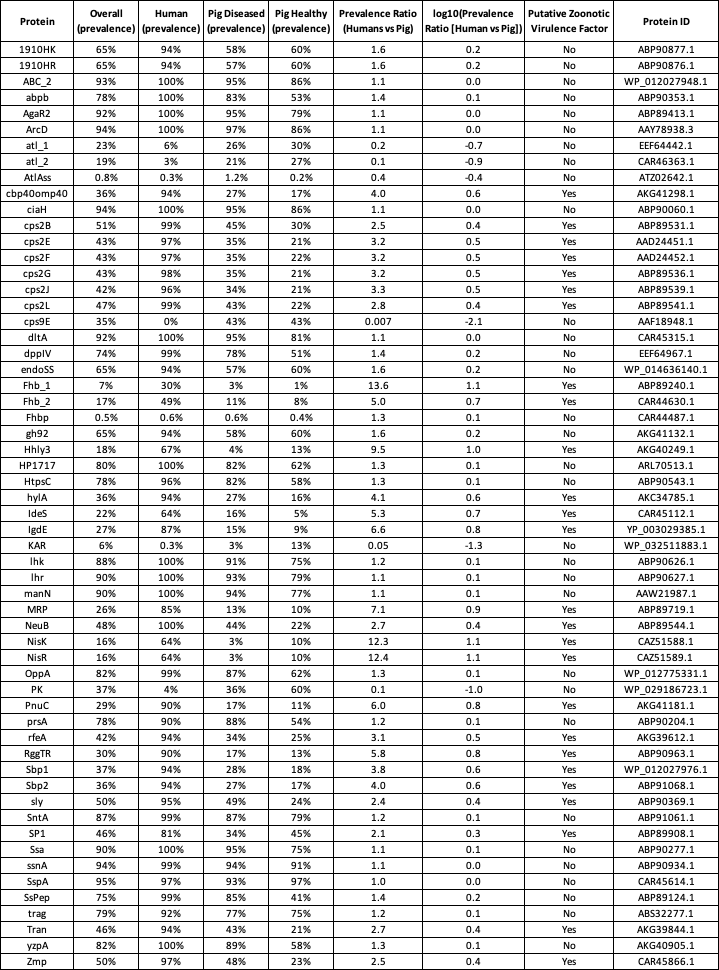
***

**Figure 2 Presence of putative virulence factors in *S. suis* CC1 isolates and the corresponding virulence factor prevalence ratio in human isolates compared to pig isolates.**(A) The 1703 assemblies were clustered using IQ-TREE^64^ based on a Roary^65^ core gene alignment of a Prokka^66^ annotated assembly. Presence of proteins in *S. suis* isolates and isolate metadata was visualized in Phandango.^19^ (B) Prevalence ratio of virulence factors in human isolates over pig isolates was based on virulence factor presence in CC1 *S. suis* genomes. Dotted line indicates a prevalence ratio of 2.

**Table 5 Prevalence of putative virulence factors within *S. suis* isolates from CC1.**

^^

## **References**

1 Jolley KA, Bray JE, Maiden MCJ. Open-access bacterial population genomics: BIGSdb software, the PubMLST.org website and their applications. *Wellcome open Res* 2018; **3**: 124.

2 Souvorov A, Agarwala R, Lipman DJ. SKESA: strategic k-mer extension for scrupulous assemblies. *Genome Biol* 2018; **19**: 153.

3 Gurevich A, Saveliev V, Vyahhi N, Tesler G. QUAST: quality assessment tool for genome assemblies. *Bioinformatics* 2013; **29**: 1072–5.

4 Ewels P, Magnusson M, Lundin S, Käller M. MultiQC: summarize analysis results for multiple tools and samples in a single report. *Bioinformatics* 2016; **32**: 3047–8.

5 Jain C, Rodriguez-R LM, Phillippy AM, Konstantinidis KT, Aluru S. High throughput ANI analysis of 90K prokaryotic genomes reveals clear species boundaries. *Nat Commun* 2018; **9**: 5114.

6 Putten van der BCL. network_analysis_scripts. GitHub. 2020. https://github.com/boasvdp/network_analysis_scripts (accessed Oct 3, 2020).

7 Shannon P, Markiel A, Ozier O, *et al.* Cytoscape: a software environment for integrated models of biomolecular interaction networks. *Genome Res* 2003; **13**: 2498–504.

8 Seemann T. Abricate: mass screening of contigs for antimicrobial and virulence genes. *Dep Microbiol Immunol Univ Melbourne, Melbourne, Aust Available online https//github com/tseemann/abricate (accessed 28 Febr 2019)* 2018.

9 Athey TBT, Teatero S, Lacouture S, Takamatsu D, Gottschalk M, Fittipaldi N. Determining Streptococcus suis serotype from short-read whole-genome sequencing data. *BMC Microbiol* 2016; **16**: 162.

10 Fu L, Niu B, Zhu Z, Wu S, Li W. CD-HIT: accelerated for clustering the next-generation sequencing data. *Bioinformatics* 2012; **28**: 3150–2.

11 Hadfield J, Croucher NJ, Goater RJ, Abudahab K, Aanensen DM, Harris SR. Phandango: an interactive viewer for bacterial population genomics. *Bioinformatics* 2017; **34**: 292–3.

12 Zhang Y, Ding D, Liu M, *et al.* Effect of the glycosyltransferases on the capsular polysaccharide synthesis of Streptococcus suis serotype 2. *Microbiol Res* 2016; **185**: 45–54.

13 Feng Y, Cao M, Shi J, *et al.* Attenuation of Streptococcus suis virulence by the alteration of bacterial surface architecture. *Sci Rep* 2012; **2**: 710.

14 Benga L, Goethe R, Rohde M, Valentin-Weigand P. Non-encapsulated strains reveal novel insights in invasion and survival of Streptococcus suis in epithelial cells. *Cell Microbiol* 2004; **6**: 867–81.

15 Ferrando ML, de Greeff A, van Rooijen WJ, *et al.* Host-pathogen Interaction at the Intestinal Mucosa Correlates With Zoonotic Potential of Streptococcus suis. *J Infect Dis* 2015; **212**: 95–105.

16 Roy D, Grenier D, Segura M, Mathieu-Denoncourt A, Gottschalk M. Recruitment of Factor H to the Streptococcus suis Cell Surface is Multifactorial. *Pathogens* 2016; **5**. DOI:10.3390/pathogens5030047.

17 Lin L, Xu L, Lv W, *et al.* An NLRP3 inflammasome-triggered cytokine storm contributes to streptococcal toxic shock-like syndrome (STSLS). *PLoS Pathog* 2019; **15**. DOI:10.1371/journal.ppat.1007795.

18 Graveline R, Segura M, Radzioch D, Gottschalk M. TLR2-dependent recognition of Streptococcus suis is modulated by the presence of capsular polysaccharide which modifies macrophage responsiveness. *Int Immunol* 2007; **19**: 375–89.

19 Salasia SI, Lammler C, Herrmann G. Properties of a Streptococcus suis isolate of serotype 2 and two capsular mutants. *Vet Microbiol* 1995; **45**: 151–6.

20 Lalonde M, Segura M, Lacouture S, Gottschalk M. Interactions between Streptococcus suis serotype 2 and different epithelial cell lines. *Microbiology-Sgm* 2000; **146**: 1913–21.

21 Huang W, Chen Y, Li Q, *et al.* LytR plays a role in normal septum formation and contributes to full virulence in Streptococcus suis. *Vet Microbiol* 2021; **254**. DOI:10.1016/j.vetmic.2021.109003.

22 Meijerink M, Ferrando ML, Lammers G, Taverne N, Smith HE, Wells JM. Immunomodulatory effects of Streptococcus suis capsule type on human dendritic cell responses, phagocytosis and intracellular survival. *PLoS One* 2012; **7**: e35849.

23 Zaccaria E, Cao R, Wells JM, van Baarlen P. A Zebrafish Larval Model to Assess Virulence of Porcine Streptococcus suis Strains. *PLoS One* 2016; **11**: e0151623.

24 Vadeboncoeur N, Segura M, Al-Numani D, Vanier G, Gottschalk M. Pro-inflammatory cytokine and chemokine release by human brain microvascular endothelial cells stimulated by Streptococcus suis serotype 2. *FEMS Immunol Med Microbiol* 2003; **35**: 49–58.

25 Schwerk C, Papandreou T, Schuhmann D, *et al.* Polar invasion and translocation of Neisseria meningitidis and Streptococcus suis in a novel human model of the blood-cerebrospinal fluid barrier. *PLoS One* 2012; **7**: e30069.

26 Auger JP, Christodoulides M, Segura M, Xu J, Gottschalk M. Interactions of Streptococcus suis serotype 2 with human meningeal cells and astrocytes. *BMC Res Notes* 2015; **8**: 607.

27 Esgleas M, Lacouture S, Gottschalk M. Streptococcus suis serotype 2 binding to extracellular matrix proteins. *FEMS Microbiol Lett* 2005; **244**: 33–40.

28 Jobin MC, Gottschalk M, Grenier D. Upregulation of prostaglandin E2 and matrix metalloproteinase 9 production by human macrophage-like cells: synergistic effect of capsular material and cell wall from Streptococcus suis. *Microb Pathog* 2006; **40**: 29–34.

29 Norton PM, Rolph C, Ward PN, Bentley RW, Leigh JA. Epithelial invasion and cell lysis by virulent strains of Streptococcus suis is enhanced by the presence of suilysin. *FEMS Immunol Med Microbiol* 1999; **26**: 25–35.

30 Seitz M, Baums CG, Neis C, *et al.* Subcytolytic effects of suilysin on interaction of Streptococcus suis with epithelial cells. *Vet Microbiol* 2013; **167**: 584–91.

31 Zhang S, Zheng Y, Chen S, *et al.* Suilysin-induced Platelet-Neutrophil Complexes Formation is Triggered by Pore Formation-dependent Calcium Influx. *Sci Rep* 2016; **6**: 36787.

32 Chen S, Xie W, Wu K, *et al.* Suilysin Stimulates the Release of Heparin Binding Protein from Neutrophils and Increases Vascular Permeability in Mice. *Front Microbiol* 2016; **7**: 1338.

33 Lun S, Perez-Casal J, Connor W, Willson PJ. Role of suilysin in pathogenesis of Streptococcus suis capsular serotype 2. *Microb Pathog* 2003; **34**: 27–37.

34 Jobin MC, Fortin J, Willson PJ, Gottschalk M, Grenier D. Acquisition of plasmin activity and induction of arachidonic acid release by Streptococcus suis in contact with human brain microvascular endothelial cells. *FEMS Microbiol Lett* 2005; **252**: 105–11.

35 Pian Y, Wang P, Liu P, *et al.* Proteomics identification of novel fibrinogen-binding proteins of Streptococcus suis contributing to antiphagocytosis. *Front Cell Infect Microbiol* 2015; **5**: 19.

36 Pian Y, Li X, Zheng Y, Wu X, Yuan Y, Jiang Y. Binding of Human Fibrinogen to MRP Enhances Streptococcus suis Survival in Host Blood in a alphaXbeta2 Integrin-dependent Manner. *Sci Rep* 2016; **6**: 26966.

37 Li Q, Fu Y, Ma C, *et al.* The non-conserved region of MRP is involved in the virulence of Streptococcus suis serotype 2. *Virulence* 2017; **8**: 1274–89.

38 Zhang W, Liu G, Tang F, *et al.* Pre-absorbed immunoproteomics: a novel method for the detection of Streptococcus suis surface proteins. *PLoS One* 2011; **6**: e21234.

39 Li Q, Ma C, Fu Y, *et al.* Factor H specifically capture novel Factor H-binding proteins of Streptococcus suis and contribute to the virulence of the bacteria. *Microbiol Res* 2017; **196**: 17–25.

40 Wang J, Kong D, Zhang S, *et al.* Interaction of fibrinogen and muramidase-released protein promotes the development of Streptococcus suis meningitis. *Front Microbiol* 2015; **6**: 1001.

41 Pian Y, Gan S, Wang S, *et al.* Fhb, a novel factor H-binding surface protein, contributes to the antiphagocytic ability and virulence of Streptococcus suis. *Infect Immun* 2012; **80**: 2402–13.

42 Li X, Liu P, Gan S, *et al.* Mechanisms of Host-Pathogen Protein Complex Formation and Bacterial Immune Evasion of Streptococcus suis Protein Fhb. *J Biol Chem* 2016; **291**: 17122–32.

43 Kong D, Chen Z, Wang J, *et al.* Interaction of factor H-binding protein of Streptococcus suis with globotriaosylceramide promotes the development of meningitis. *Virulence* 2017; **8**: 1290–302.

44 Ferrando ML, Willemse N, Zaccaria E, Pannekoek Y, van der Ende A, Schultsz C. Streptococcal Adhesin P (SadP) contributes to Streptococcus suis adhesion to the human intestinal epithelium. *PLoS One* 2017; **12**: e0175639.

45 Madar Johansson M, Bélurier E, Papageorgiou AC, *et al.* The binding mechanism of the virulence factor Streptococcus suis adhesin P subtype to globotetraosylceramide is associated with systemic disease. *J Biol Chem* 2020; **295**: 14305–24.

46 Li Q, Liu H, Du D, *et al.* Identification of Novel Laminin- and Fibronectin-binding Proteins by Far-Western Blot: Capturing the Adhesins of Streptococcus suis Type 2. *Front Cell Infect Microbiol* 2015; **5**: 82.

47 Jiang H, Wu T, Liu J, *et al.* Caveolae/rafts protect human cerebral microvascular endothelial cells from Streptococcus suis serotype 2 α-enolase-mediated injury. *Vet Microbiol* 2021; **254**. DOI:10.1016/j.vetmic.2021.108981.

48 Liu H, Lei S, Jia L, *et al.* Streptococcus suis serotype 2 enolase interaction with host brain microvascular endothelial cells and RPSA-induced apoptosis lead to loss of BBB integrity. *Vet Res* 2021; **52**. DOI:10.1186/s13567-020-00887-6.

49 Feng Y, Pan X, Sun W, *et al.* Streptococcus suis enolase functions as a protective antigen displayed on the bacterial cell surface. *J Infect Dis* 2009; **200**: 1583–92.

50 Chen B, Zhang A, Xu Z, Li R, Chen H, Jin M. Large-scale identification of bacteria-host crosstalk by affinity chromatography: capturing the interactions of Streptococcus suis proteins with host cells. *J Proteome Res* 2011; **10**: 5163–74.
