# Supplementary material for: Identification of Streptococcus suis putative zoonotic virulence factors: A systematic review and genomic meta-analysis
Source: Virulence. 2021 Nov 25;12(1):2787–97. doi: 10.1080/21505594.2021.1985760 (PMC8632099; doi:10.1080/21505594.2021.1985760)
Supplement: Supplemental Material [file KVIR_A_1985760_SM7428.zip › supplementary/2021.09.22_Appendix2_Data_Extraction_Table_PDFView.pdf]

Authours (year)	DOI	Factor	Function	Serotype	Strain	Sequence Type	isolated from	Human model	Experimental outcome in human model	Representative body sites	Factor or Regulator	Virulence factor studied in	Growth curve	Growth affected	Complemented	Protein ID	Core or Accessory genome
Auger et al., 2015	10.1186/s13104-015-1581-2	cps2F	CPS biosynthesis	SS2	P1/7	ST1	Pig	Meningeal cells, Fetal astrocyte cells	Increased adherence and invasion, increased adherence and invasion	BBB	Factor	KO	Yes	No	No	CAR45153.1	Accessory
Auger et al., 2015	10.1186/s13104-015-1581-2	dlta	LTA D-alanylation	SS2	31533	ST1	Pig	Meningeal cells, Fetal astrocyte cells	Not affected adherence or invasion, not affected adherence but attenuated invasion	BBB	Factor	KO	Yes	No	No	CAR45315.1 (P1/7 derived)	Accessory
Auger et al., 2015	10.1186/s13104-015-1581-2	pgdA	N-deacetylation of peptidoglycan	SS2	31533	ST1	Pig	Meningeal cells, Fetal astrocyte cells	Not affected adherence or invasion, not affected adherence or invasion	BBB	Factor	KO	No	NA	No	CAR47008.1	Core
Auger et al., 2015	10.1186/s13104-015-1581-2	sly	Hemolysin	SS2	31533	ST1	Pig	Meningeal cells, Fetal astrocyte cells	Not affected adherence or invasion, not affected adherence but attenuated invasion	BBB	Factor	KO	Yes	No	No	AY139031.1	Accessory
Auger et al., 2017	10.1371/journal.pone.0181920	atl	autolysin	SS2	89-1591	ST25	Pig	Fibronectin	Not affected adhesion	ECM, Blood	Factor	KO	Yes	No	No	EEF64442.1	Accessory
Auger et al., 2017	10.1371/journal.pone.0181920	atl	autolysin	SS2	P1/7	ST1	Pig	Fibronectin	Not affected adhesion	ECM, Blood	Factor	KO	Yes	No	No	CAR46363.1	Accessory
Auger et al., 2017	10.1371/journal.pone.0181920	atl	autolysin	SS2	SC84	ST7	Human	Fibronectin	Not affected adhesion	ECM, Blood	Factor	KO	Yes	No	No	CAZ51936.1	Accessory
Auger et al., 2017	10.1371/journal.pone.0181920	dppIV	dipeptidyl peptidase IV binding of fibronectin	SS2	89-1591	ST25	Pig	Fibronectin	Not affected adherence	ECM, Blood	Factor	KO	Yes	No	No	EEF64967.1	Accessory
Auger et al., 2017	10.1371/journal.pone.0181920	dppIV	dipeptidyl peptidase IV binding of fibronectin	SS2	P1/7	ST1	Pig	Fibronectin	Not affected adherence	ECM, Blood	Factor	KO	Yes	No	No	CAR44490.1	Accessory
Auger et al., 2017	10.1371/journal.pone.0181920	dppIV	dipeptidyl peptidase IV binding of fibronectin	SS2	SC84	ST7	Human	Fibronectin	Not affected adherence	ECM, Blood	Factor	KO	Yes	No	No	CAZ50957.1	Accessory
Bai et al., 2020	10.1080/21505594.2020.1838740	SspA	Surface associated subtilisin-like protease	SS2	P1/7	ST1	Pig	C5a	Not cleaved	Blood	Factor	Rec. Protein	NA	NA	NA	CAR45614	Accessory
Bai et al., 2020	10.1080/21505594.2020.1838740	SspB	Surface associated subtilisin-like protease B	SS2	ZY05719	ST7	Pig	C5a	Not cleaved	Blood	Factor	Rec. Protein	NA	NA	NA	AKG41190	Core
Bai et al., 2020	10.1080/21505594.2020.1838740	yzpA	YSIRK-G/S related zinc-binding protein A	SS2	ZY05719	ST7	Pig	HEp2	Not affected adhesion	Epithelium	Factor	KO	No	NA	Yes	AKG40905.1	Accessory
Benga et al., 2004	10.1111/j.1462-5822.2004.00409.x	cps2E+F	CPS biosynthesis	SS2	S10 (strain 10)	ST1	Pig	HEp2	Increased adherence and invasion	Epithelium	Factor	KO	No	NA	No	AAD24451.1 (CpsE) and AAD24452.1(CpsF)	Accessory
Bonifait and Grenier, 2011	10.1186/1471-2180-11-47	SspA	Surface associated subtilisin-like protease	SS2	P1/7	ST1	Pig	U937	Increased pro-inflammatory cytokines	Blood	Factor	KO	No	NA	No	CAR45614.1	Accessory
Bonifait et al., 2011	10.1016/j.vetmic.2010.09.024	SspA	Surface associated subtilisin-like protease	SS2	P1/7	ST1	Pig	BMEC	Decreased cell viability	BBB	Factor	Rec. Protein	NA	NA	NA	CAR45614.0	Accessory
Bonifait et al., 2010	10.1186/1471-2180-10-42	SspA	Surface associated subtilisin-like protease	SS2	P1/7	ST1	Pig	Blood	Attenuated survival	Blood	Factor	KO	Yes	Slower	No	CAR45614.1	Accessory
Cao et al., 2011	10.1007/s12275-011-1523-1	LuxS	Involved in AI-2 quorum sensing	SS2	05ZYH33	ST7	Human	HEp2, HUVEC	Attenuated adherence, attenuated adherence	Epithelium, Blood, BBB	Regulator	KO	Yes	Slower	molecule (DPD)	ABP89387.1	Core
Chang et al., 2018	10.1080/21505594.2018.1428519	VraSR	Two-component system signal transduction system	SS2	SC-19	ST7	Pig	Blood, PMNs, BMEC	Attenuated survival, increased phagocytosis, attenuated adherence	Blood, BBB	Regulator	KO	Yes	No	Yes	CAZ51151.1	Core
Charland et al., 2000	10.1128/iai.68.2.637-643.2000	aro operon	Promotor of aro operon (aroA, aroK, pheA and orf10 genes), insertion of Tn916 causes unencapsulation	SS2	S735	ST1	Pig	BMEC, HUVEC	Not affected adhesion, not affected adhesion	BBB, Blood	Factor	KO	No	NA	No	promotor	ND
Chen et al., 2011	10.1021/pr200758q	DivIVA	cell division initiation protein	SS2	SC-19	ST7	Pig	HEp2	Not affected adhesion, not affected adhesion	Epithelium	Factor	Rec. Protein	NA	NA	NA	WP_011922043.1	Core
Chen et al., 2011	10.1021/pr200758q	DnaK	Chaperone protein/Surface associated adhesing protein	SS2	SC-19	ST7	Pig	HEp2	Decreased adhesion	Epithelium	Regulator/ Factor	Rec. Protein	NA	NA	NA	ARL69286.1	Core
Chen et al., 2011	10.1021/pr200758q	eno	Enolase, surface associated protein binding to factor H, fibrinogen fibronectin and plasminogen	SS2	SC-19	ST7	Pig	HEp2	Decreased adhesion	Epithelium	Factor	Rec. Protein	NA	NA	NA	ARL70313.1	Core
Chen et al., 2011	10.1021/pr200758q	FBPS	Fibornectin binding protein	SS2	SC-19	ST7	Pig	HEp2	Adhesion not affected	Epithelium	Factor	Rec. Protein	NA	NA	NA	WP_012027427.1	Core
Chen et al., 2011	10.1021/pr200758q	HAM1	fused deoxyribonucleotide triphosphate pyrophosphatase/unknown domain-containing protein	SS2	SC-19	ST7	Pig	HEp2	Decreased adhesion	Epithelium	Factor	Rec. Protein	NA	NA	NA	ARL70502.1	Core
Chen et al., 2011	10.1021/pr200758q	HP0272 (Fhb)	Cell wall anchored protein, function unknown	SS2	SC-19	ST7	Pig	HEp2	Adhesion not affected	Epithelium	Factor	Rec. Protein	NA	NA	NA	ABP89240.1	Accessory
Chen et al., 2011	10.1021/pr200758q	Pep F	oligoendopeptidase F	SS2	SC-19	ST7	Pig	HEp2	Adhesion not affected	Epithelium	Factor	Rec. Protein	NA	NA	NA	ARL70086.1	Core
Chen et al., 2011	10.1021/pr200758q	ssnA (DNAse)	Secreted Dnase, NET degradation	SS2	SC-19	ST7	Pig	HEp2	Adhesion not affected	Epithelium	Factor	Rec. Protein	NA	NA	NA	ABP90934.1	Core
Chen et al., 2016	10.3389/fmicb.2016.01338	sly	Hemolysin	SS2	05ZYH33	ST7	Human	Blood, PMNs	Attentuated Ca2+ dependent heparin binding protein (HBP) secretion, decreased PMN degranulation	Blood	Factor	KO	Yes	No	Yes	ABP90369.1	Accessory
de Buhr et al., 2014	10.1099/mic.0.072199-0	ssnA (DNAse)	Secreted Dnase, NET degradation	SS2	S10 (strain 10)	ST1	Pig	Blood, Neutrophils	Not affected survival, attenuated in NET degradation and survival	Blood	Factor	KO	Yes	No	No	WP_012775354.1	Core
de Buhr et al., 2015	10.1099/mic.0.000040	EndA (ssnA)	Degradation of NET (especially during exp. Growth), cell wall anchored	SS2	S10 (strain 10)	ST1	Pig	Neutrophils	Attenuated NET degradation	Blood	Factor	KO	Yes	No	No	CAR47454.1	Core
Deng et al., 2018	10.3389/fimmu.2018.01063	SntA	Putative heme-binding protein (cell wall-anchored)	SS2	SC-19	ST7	Pig	Blood, PMNs, serum, serum HI, complement	Attenuated survival, atternuated survival, attenuated survival, not affected survival, attenuated complement consumption	Blood	Factor	KO	Yes	No	Yes	ABP91061.1	Accessory
Du et al., 2014	10.1016/j.micres.2014.01.002	GdpP	Degradation of host c-di-AMP	SS2	HA9801	ST378	Pig	HEp2	Attenuated adherence and invasion	Epithelium	Regulator	KO	Yes	No	Yes	ABP93318.1	Core
Dumesnil et al., 2018	10.1186/s13567-018-0606-y	Zmp	zinc metalloprotease	SS2	P1/7	ST1	Pig	IgA1, MUC16	Like wildtype IgA1 is not cleaved, partly attenuated cleavage	Epithelium	Factor	KO	Yes	No	Yes	CAR45866.1	Accessory
Esgleas et al., 2005	10.1016/j.femsle.2005.01.017	cps	CPS biosynthesis	SS2	S735	ST1	Pig	Fibrinogen	Increased adherence	ECM, Blood	Factor	KO	No	NA	No	not found	Accessory
Fang et al., 2017	10.4142/jvs.2017.18.4.439	Stp	Serine/threonine protein phosphatase	SS2	ZJ081101	not found	Pig	HEp2	Increased adhesion	Epithelium	Regulator	KO	No	NA	No	not found	Core
Feng et al., 2016	10.1038/srep21241	CodY	Transcriptional regulator	SS2	SC-19	ST7	Pig	HEp2	Attenuated adherence and invasion	Epithelium	Regulator	KO	Yes	No	No	ARL69324.1	Core
Feng et al., 2012	10.1038/srep00710	cps2B	CPS biosynthesis	SS2	05ZYH33	ST7	Human	HEp2, HUVEC, Blood	Increased adherence and invasion, Increased adherence, attenuated survival	Epithelium, Blood, BBB	Factor	KO	Yes	No	Yes	ABP89531.1	Accessory
Feng et al., 2012	10.1038/srep00710	NeuB	N-acetylneuramic acid (sialic acid) synthetase, CPS biosynthesis, thinner CPS	SS2	05ZYH33	ST7	Human	HEp2, HUVEC	Increased adherence	Epithelium, Blood, BBB	Factor	KO	Yes	No	Yes	ABP89544.1	Accessory
Feng et al., 2009	10.1086/644602	eno	Enolase, surface associated protein binding to factor H, fibrinogen fibronectin and plasminogen	SS2	05ZYH33	ST7	Human	HEp2	Attenuated adherence	Epithelium	Factor	Pur. Protein	NA	NA	NA	ACS66679.1	Core
Ferrando et al., 2015	10.1093/infdis/jiu813	cps2E+F	CPS biosynthesis	SS2	S10 (strain 10)	ST1	Pig	Caco-2	Increased adhesion, invasion and translocation	Epithelium	Factor	KO	No	NA	No	AAD24451.1 and AAD24452.1	Accessory
Ferrando et al., 2015	10.1093/infdis/jiu813	cpsE	CPS biosynthesis	SS9	8067	ST136	Pig	Caco-2	Increased adherence and invasion	Epithelium	Factor	KO	No	NA	No	AF155805_2	Accessory
Ferrando et al., 2015	10.1093/infdis/jiu813	sly	Hemolysin	SS2	P1/7	ST1	Pig	Caco-2	Translocation not affected	Epithelium	Factor	KO	No	NA	No	CAR46566.1	Accessory
Ferrando et al., 2017	10.1371/journal.pone.0175639	SadP	Adhesin: recognizes galabiose present at the terminal epitope of Gb3/CD77 receptor (cell-wall associated)	SS2	S10 (strain 10)	ST1	Pig	Caco-2	Attenuated adhesion and translocation	Epithelium	Factor	KO	Yes	No	No	WP_012775427.1	Accessory
Fulde et al., 2014	10.3389/fcimb.2014.00107	ArcD	Membrane-associated arginine-ornithine antiporter, intracellular survival in acidic environment	SS2	S10 (strain 10)	ST1	Pig	HEp2	Attenuated intracellular survival	Epithelium	Factor	KO	Yes	Slower	No	AAY78938.3	Core
Fulde et al., 2011	10.1099/mic.0.043067-0	ArgR	Transriptional regulator of arcABC operon	SS2	S10 (strain 10)	ST1	Pig	HEp2	Attenuated intracellular survival	Epithelium	Regulator	KO	Yes	Slower	No	WP_002941715.1	Core
Gao et al., 2016	10.3389/fcimb.2016.00044	GidA	tRNA modification enzyme (translational regulator)	SS2	SC-19	ST7	Pig	HEp2	Attenuated adherence and invasion	Epithelium	Regulator	KO	Yes	Slower	No	ABP91129.1	Core
Gao et al., 2019	10.3389/fcimb.2019.00173	mnmE	tRNA modification GTPase (translational regulator)	SS2	SC-19	ST7	Pig	HEp2	Attenuated adhesion and invasion	Epithelium	Regulator	KO	Yes	Slower	Yes	ABP90420.1	Core
Ge et al., 2009	10.1007/s00284-009-9425-8	dppIV	dipeptidyl peptidase IV binding of fibronectin	SS2	05ZYH33	ST7	Human	HEp2	Attenuated adherence	Epithelium	Factor	KO	Yes	No	Yes	ABP89165.1	Accessory

Gong et al., 2019	10.1016/j.micpath.2019.103730	sRNA34	Regulates gene expression	SS2	05ZYH33	ST7	Human	HEp2	Not affected adhesion	Epithelium	Regulator	KO	Yes	No	Yes	NA	NA
Graveline et al., 2007	10.1093/intimm/dxm003	cps	CPS biosynthesis	SS2	31533	ST1	Pig	THP-1	Increased TNF and IL-1 secretion via TLR2 and CD14 modulation and decreased MCP-1 secretion	Blood	Factor	KO	No	NA	No	not found	Accessory
Grenier and Bodet, 2008	10.1111/j.1574-695X.2008.00476.x	aro operon	Promotor of aro operon (aroA, aroK, pheA and orf10 genes), deletion causes unencapsulation	SS2	S735	ST1	Pig	BMEC	Increased shedding of ICAM-1	BBB	Factor	KO	No	NA	No	promotor	ND
Haas et al., 2014	10.1186/1756-0500-7-424	ssnA (DNAse)	Secreted Dnase, NET degradation	SS2	P1/7	ST1	Pig	U937	Attenuated pro-inflammatory cytokine production	Blood	Factor	KO	No	NA	No	CAR47454.1	Core
Haas et al., 2015	10.1186/s13104-015-1692-9	hylA	hyaluronic acid lysase	SS2	P1/7	ST1	Pig	BMEC	At low concentration increased adherence, at high concentration decreased adherence	BBB	Factor	Rec. Protein	NA	NA	NA	AKC34785.1	Accessory
Han et al., 2012	10.1099/mic.0.057448-0	Ihk+Irr	Two-component system signal transduction system	SS2	05ZYH33	ST7	Human	HEp2, HeLa	Attenuated adherence, attenuated adherence	Epithelium	Regulator	KO	Yes	No	Yes	ABP90626.1 (IhK) and ABP90627.1 (IhR)	Accessory and Accessory
Hu et al., 2014	10.1038/srep04140	BgaC	Surface-anchored bèta-galactosidase	SS2	05ZYH33	ST7	Human	HEp2	Not affected adherence	Epithelium	Factor	KO	No	NA	No	WP_011922008.1	Core
Huang et al., 2015	10.1016/j.micres.2014.08.001	abpb/AdpS	Surface-associated arginine peptidase	SS2	SC-19	ST7	Pig	HEp2	Attenuated adherence	Epithelium	Factor	KO	Yes	No	No	ABP90353.1	Accessory
Huang et al., 2021	10.1016/j.vetmic.2021.109003	lytR (cps2A)	Transcriptional Regulator	SS2	05ZYH33	ST7	Human	Blood, PMN	Attenuated survival, Attenuated survival	Blood	Regulator	KO	Yes	No	Yes	ABP90824.1	Core
Jiang et al., 2021	10.1016/j.vetmic.2021.108981	eno	Enolase, surface associated protein binding to factor H, fibrinogen fibronectin and plasminogen	SS2	JZLQ022	not found	Pig	hCMEC/D3, HEK293T (RPSA transfected)	Increases RPSA on cell surface;induces apoptosis via RSPA and apoptosis is inhibited by caveolae, Eno interacts with RSPA and promotes CAV1 RSPA interaction	BBB	Factor	Rec. Protein	NA	NA	NA	not found	Core
Jiang et al., 2016	10.3389/fcimb.2016.00172	VirD4 (trag)	Key component in putative type-IV-like secretion system	SS2	HA9801	ST378	Pig	THP-1	Increased phagocytosis	Blood	Factor	KO	Yes	No	No	ABP89939.1	Accessory
Jobin et al., 2004	10.1128/iai.72.1.606-610.2004	GAPDH	Surface associated protein binding to plasmin	SS2	S735	ST1	Pig	Plasminogen	Reduced binding	Blood	Factor	KO	No	NA	No	AFQ99656.1	Core
Jobin et al., 2005	10.1016/j.femsle.2005.08.044	sly	Hemolysin	SS2	31533	ST1	Pig	BMEC	Attenuated arachidonic acid release (precursos for BBB permeability)	BBB	factor	KO	Yes	No	No	AY139031.1	Accessory
Jobin et al., 2006	10.1016/j.micpath.2005.10.003	cps	CPS biosynthesis	SS2	S735	ST1	Pig	U937	Induces PGE2 and MMP-9 secretion	Blood	Factor	Pur. Protein	NA	NA	NA	NA	Accessory
Jobin et al., 2006	10.1016/j.micpath.2005.10.003	CW	Cell Wall	SS2	S735	ST1	Pig	U937	PGE2 and MMP-9 secretion unaffected	Blood	Factor	Pur. Protein	NA	NA	NA	NA	NA
Johansson et al., 2020	10.1074/jbc.RA120.014818	SadP (Pn/Po)	Adhesin: recognizes galabiose present at the terminal epitope of Gb3/CD77 receptor (cell-wall associated)	SS2	D282	ST1	Pig	Human Gb3, Human Gb4, EA.hy926	Strong binding (Pn & Po), occasional binding (Pn) no binding (Po), Rec. SadP binding and KO attenuated adhesion	Blood	Factor	KO and Rec. Protein	No	NA	Blocking	AM946016.1 (SadPn), CP008921.1 (SadPo)	Accessory
Ju et al., 2012	10.1128/JB.06231-11	atl	autolysin	SS2	HA9801	ST378	Pig	HEp2, Zebrafish	Attenuated adherence, attenuated survival	Epithelium, Blood	Factor	KO	Yes	No	Yes	EF563971	Accessory
Kong et al., 2017	10.1080/21505594.2017.1317426	Fhb	(Cell envelope assocaited) Factor H/C3b/C3d binding protein	SS2	05ZYH33	ST7	Human	hCMEC/D3	Attenuated translocation and adhesion	BBB	Factor	KO	Yes	No	Yes	CP000408.1	Accessory
Lalonde et al., 2000	10.1099/00221287-146-8-1913	cps	CPS biosynthesis	SS2	S735	ST1	Pig	A549, HeLa	Increased adherence, increased adherence	Epithelium	Factor	KO	No	NA	No	AFR00009.1	Accessory
Lebel et al., 2018	10.1016/j.micpath.2018.06.029	dppIV	Subtilisin-like protease (surface associated)	SS2	S735	ST1	Pig	BMEC	Increased IL-8 secretion	BBB	factor	Rec. Protein	NA	NA	NA	AFQ99690.1	Accessory
Li et al., 2011	10.1016/j.vetmic.2010.08.005	ciaRH	Two-component system signal transduction system	SS2	05ZYH33	ST7	Pig	HEp2	Attenuated adherence	Epithelium	Regulator	KO	Yes	Slower	No	ABP90060.1 (CiaH) and ABP90061.1 (CiaR)	ciaH Accessory ciaR Core
M. Li et al., 2017	10.1371/journal.pone.0169791	ssnA (DNAse)	Secreted Dnase, NET degradation	SS2	GD01	not found	Pig	HEp2	Attenuated adherence and invasion	Epithelium	Factor	KO	Yes	Slower	Yes	WP_012775354.1	Core
M. Li et al., 2015	10.1080/21505594.2015.1056971	HtpsC	Putative zinc binding protein. Putative contribution to complement deposition inhibition. Binds fibronectin and laminin (surface-exposed protein)	SS2	05ZYH33	ST7	Human	HEp2, Blood, Fibronectin, Laminin	Attenuated adherence, attenuated survival, dose dependent adherence, dose dependent adherence	Epithelium, Blood, ECM	Factor	KO	Yes	No	Yes	ABP90543.1	Accessory
Q. Li et al., 2017c	10.1016/j.micres.2016.11.011	EF-Tu	Elongation factor Tu	SS2	ZY05719	ST7	Pig	Factor H	Dose dependent binding, blocking adhesin decreases factor H binding	Blood	Factor	Rec. Protein	NA	NA	NA	WP_012775011.1	Core
Q. Li et al., 2017c	10.1016/j.micres.2016.11.011	eno	Enolase, surface associated protein binding to factor H, fibrinogen fibronectin and plasminogen	SS2	ZY05719	ST7	Pig	Factor H	Dose dependent binding, blocking adhesin decreases binding factor H binding	Blood	Factor	Rec. Protein	NA	NA	NA	WP_002935704.1	Core
Q. Li et al., 2017c	10.1016/j.micres.2016.11.011	FBA	fructose-bisphosphate aldolase	SS2	ZY05719	ST7	Pig	Factor H	Dose dependent binding, blocking adhesin decreases binding to factor H	Blood	Factor	Rec. Protein	NA	NA	NA	WP_029185467.1	Core
Q. Li et al., 2017c	10.1016/j.micres.2016.11.011	FBPS	Fibronectin binding protein	SS2	ZY05719	ST7	Pig	Factor H	Dose dependent binding, blocking adhesin decreases binding factor H binding	Blood	Factor	Rec. Protein	NA	NA	NA	ABB71989.1	Core
Q. Li et al., 2017c	10.1016/j.micres.2016.11.011	Fhb	(Cell envelope assocaited) Factor H/C3b/C3d binding protein	SS2	ZY05719	ST7	Pig	Factor H	Dose dependent binding	Blood	Factor	Rec. Protein	NA	NA	NA	AKG39686.1	Accessory
Q. Li et al., 2017c	10.1016/j.micres.2016.11.011	GAPDH	Surface associated protein binding to plasmin	SS2	ZY05719	ST7	Pig	Factor H	Dose dependent binding, blocking adhesin decreases binding to factor H	Blood	Factor	Rec. Protein	NA	NA	NA	AAZ78247.1	Core
Q. Li et al., 2017c	10.1016/j.micres.2016.11.011	KAR	3-ketoacyl-ACP reductase	SS2	ZY05719	ST7	Pig	Factor H	Dose dependent binding, blocking adhesin decreases binding to factor H	Blood	Factor	Rec. Protein	NA	NA	NA	WP_032511883.1	Accessory
Q. Li et al., 2017c	10.1016/j.micres.2016.11.011	MRP1	Muramidase released protein (non conserved region), binds fibrinogen and fibrinogen binding protein (cell wall-anchored)	SS2	ZY05719	ST7	Pig	Factor H	Dose dependent binding, factor H binding is unaffected by anti-MRP1 IgG	Blood	Factor	Rec. Protein	NA	NA	NA	CAA45781.1	Accessory
Q. Li et al., 2017c	10.1016/j.micres.2016.11.011	MRP2	Muramidase released protein (conserved region), binds fibrinogen and fibrinogen binding protein (cell wall-anchored)	SS2	ZY05719	ST7	Pig	Factor H	Dose dependent binding, blocking MRP2 decreases binding to factor H	Blood	Factor	Rec. Protein	NA	NA	NA	CAA45781.1	Accessory
Q. Li et al., 2017c	10.1016/j.micres.2016.11.011	PK	pyruvate kinase	SS2	ZY05719	ST7	Pig	Factor H	Dose dependent binding, blocking adhesin decreases binding to factor H	Blood	Factor	Rec. Protein	NA	NA	NA	WP_029186723.1	Accessory
Q. Li et al., 2017c	10.1016/j.micres.2016.11.011	SSu0186 (Fhbp)	Factor H binding protein	SS2	ZY05719	ST7	Pig	Factor H	Dose dependent binding	Blood	Factor	Rec. Protein	NA	NA	NA	ABP89164.1	Accessory
Q. Li et al., 2015	10.3389/fcimb.2015.00082	ABC	Amino acid ABC transporter permease	SS2	ZY05719	ST7	Pig	Laminin, HEp2	Dose dependent binding, rec. protein not affected adhesion	ECM, Epithelium	Factor	Rec. Protein	NA	NA	NA	WP_012775143.1	Core
Q. Li et al., 2015	10.3389/fcimb.2015.00082	EF-Tu	Elongation factor Tu	SS2	ZY05719	ST7	Pig	Laminin, Fibronectin, HEp2	Dose dependent binding, dose dependent binding, rec. protein decreases adhesion	ECM, Blood, Epithelium	Factor	Rec. Protein	NA	NA	NA	WP_012775011.1	Core
Q. Li et al., 2015	10.3389/fcimb.2015.00082	eno	Enolase, surface associated protein binding to factor H, fibrinogen fibronectin and plasminogen	SS2	ZY05719	ST7	Pig	Laminin, Fibronectin, HEp2	Dose dependent binding, dose dependent binding, rec. protein decreases adhesion	ECM, Blood, Epithelium	Factor	Rec. Protein	NA	NA	NA	WP_002935704.1	Core
Q. Li et al., 2015	10.3389/fcimb.2015.00082	FBA	fructose-bisphosphate aldolase	SS2	ZY05719	ST7	Pig	Laminin, Fibronectin, HEp2	Dose dependent binding, dose dependent binding, rec. protein decreases adhesion	ECM, Epithelium, Blood	Factor	Rec. Protein	NA	NA	NA	WP_029185467.1	Core
Q. Li et al., 2015	10.3389/fcimb.2015.00082	GAPDH	Surface associated protein binding to plasmin	SS2	ZY05719	ST7	Pig	Laminin	Dose dependent binding	ECM	Factor	Rec. Protein	NA	NA	NA	AAZ78247.1	Core
Q. Li et al., 2015	10.3389/fcimb.2015.00082	IMPDH	Inosine 5′-monophosphate dehydrogenase	SS2	ZY05719	ST7	Pig	Laminin, HEp2	Dose dependent binding, rec. protein not affected adhesion	ECM, epithelium	Regulator/ Factor	Rec. Protein	NA	NA	NA	WP_023371888.1	Core
Q. Li et al., 2015	10.3389/fcimb.2015.00082	KAR	3-ketoacyl-ACP reductase	SS2	ZY05719	ST7	Pig	Laminin	Dose dependent binding	ECM	Factor	Rec. Protein	NA	NA	NA	WP_032511883.1	Accessory
Q. Li et al., 2015	10.3389/fcimb.2015.00082	LDH	Lactate dehydrogenase	SS2	ZY05719	ST7	Pig	Laminin, Fibronectin, HEp2	Dose dependent binding, dose dependent binding, rec. protein decreases adhesion	ECM, Blood, Epithelium	Factor	Rec. Protein	NA	NA	NA	WP_029999076.1	Core
Q. Li et al., 2015	10.3389/fcimb.2015.00082	OppA	Surface associated oligopeptide binding protein	SS2	ZY05719	ST7	Pig	Laminin, Fibronectin, HEp2	Dose dependent binding, dose dependent binding, rec. protein not affected adhesion	ECM, Blood, Epithelium	Factor	Rec. Protein	NA	NA	NA	WP_012775331.1	Accessory
Q. Li et al., 2017b	10.1080/21505594.2017.1313373	MRP	Muramidase released protein, binds fibrinogen and fibrinogen binding protein (cell wall-anchored)	SS2	ZY05719	ST7	Pig	HEp2, Fibronectin, Factor H, Fibrinogen	Attenuated adherence, bound, bound, bound	Epithelium, ECM, Blood	Factor	KO and Rec. Protein	Yes	No	No	ABG91742.1	Accessory

Q. Li et al., 2017a	10.1016/j.vetmic.2017.07.010	FBPS	Fibornectin binding protein	SS2	ZY05719	ST7	Pig	HEp2, Fibronectin, Factor H, Fibrinogen, Laminin, IgG, Zebrafish	Attenuated adherence and not affected invasion, binds, binds, binds, binds, binds, not affected	Epithelium, Blood, ECM	Factor	KO and Rec. Protein	Yes	No	No	AKG40748.1	Core
Li et al., 2018	10.1016/j.vetmic.2018.02.013	PnuC	Nicotinamide mononucleotide transporter (membrane protein (not a surface protein)), oxidative stress	SS2	ZY05719	ST7	Pig	HEp2, BMEC, Zebrafish	Not affected adherence and invasion, Not affected adherence and invasion, attenuated survival	Epithelium, Blood, BBB	Factor	KO	Yes	No	No	AKG41181.1	Accessory
Li et al., 2013	10.1016/j.vetmic.2012.09.004	Ssa	Adhesin: fibronectin-binding protein (surface anchored)	SS2	SC-19	ST7	Pig	HEp2, Fibronectin	Attenuated adherence and invasion, attenuated binding	Epithelium, ECM, Blood	Factor	KO	No	NA	No	ABP90277.1	Accessory
Li et al., 2016	10.1074/jbc.M116.719443	Fhb	(Cell envelope assocaited) Factor H/C3b/C3d binding protein	SS2	05ZYH33	ST7	Human	Factor H, C3b, C3b/iC3b deposition, PMN	Dose dependent binding, dose dependent binding, attenuated binding, attenuated survival	Blood	factor	KO	No	NA	Yes	ABP89240.1	Accessory
Li et al., 2014	10.1016/j.micres.2014.03.002	Tran	transcriptional Regulator	SS2	ZY05719	ST7	Pig	HEp2, Zebrafish	Attenuated adherence, attenuated survival	Epithelium, Blood	Regulator	KO	Yes	Slower	Yes	AKG39844.1	Accessory
Lin et al., 2019	10.1371/journal.ppat.1007795	cps2E+F	CPS biosynthesis	SS2	SC-19	ST7	Pig	THP-1	Increased inflammasome activation	Blood	Factor	KO	No	NA	No	ARL69511.1 (CPS2E)/ ARL69512.1 (CPS2F)	Accessory
Lin et al., 2019	10.1371/journal.ppat.1007795	dlta	LTA D-alanylation	SS2	SC-19	ST7	Pig	THP-1	Inflammasome activation unaffected	Blood	factor	KO	No	NA	No	ARL69567.1	Accessory
Lin et al., 2019	10.1371/journal.ppat.1007795	sly	Hemolysin	SS2	SC-19	ST7	Pig	THP-1	Inflammasome activation attenuated	Blood	Factor	KO	No	NA	Yes	ARL70228.1	Accessory
Lin et al., 2019	10.1371/journal.ppat.1007795	sly	only w/o hemolytic activity	SS2	SC-19	ST7	Pig	THP-1	Inflammasome activation attenuated	Blood	Factor	KO	No	NA	No	ARL70228.1	Accessory
H. Liu et al., 2019	10.1016/j.vetmic.2019.07.027	prsA	Chaperone protein (regulation)	SS2	05ZYH33	ST7	Human	HEp2	Increased adherence and decreased invasion	Epithelium	Regulator	KO	Yes	Slower	Yes	ABP90204.1	Accessory
Liu, Lei, et al., 2021	10.1186/s13567-020-00887-6	eno	Enolase, surface associated protein binding to factor H, fibrinogen fibronectin and plasminogen	SS1	JZLQ021, CVCC606	not found	Pig	hCMEC/D3, HEK293T (RPSA transfected)	Induces apoptosis, Eno interacts with RPSA and induces apoptosis via RPSA binding	BBB	Factor	Rec. Protein	NA	NA	NA	not found	Core
Liu, Ye, et al., 2021	10.1016/j.micpath.2020.104607	stk	Serine/threonine protein kinase	SS2	05ZYH33	ST7	Human	HEp2	Increased adherence, attenuated invasion	Epithelium	Regulator	KO	Yes	No	No	ABP89395.1	Core
Liu, Ye, et al., 2021	10.1016/j.micpath.2020.104607	stp1	Serine/threonine protein phosphatase	SS2	05ZYH33	ST7	Human	HEp2	Increased adherence, attenuated invasion	Epithelium	Regulator	KO	Yes	Yes	No	ABP89394.1	Core
L. Liu et al., 2019		HP1717	Cell surface associated, inducer of pro-inflammatory activity	SS2	05ZY	not found	Pig	Blood	Attenuated survival	Blood	Factor	KO	Yes	Yes	No	ARL70513.1	Accessory
Liu et al., 2018	10.1093/infdis/jix523	SsPep/SSPepO	Metallo-endopeptidase (extracellular protein) binding fibronectin	SS2	SC-19	ST7	Pig	Fibronectin, BMECs	Dose dependent binding, Attenuated adherence	Blood, ECM, BBB	Factor	KO	Yes	No	Yes	ABP89124.1	Accessory
Liu et al., 2014	10.1074/jbc.RA120.014818	Ssads	Converts AMP to adenosine (cell wall-anchored protein)	SS2	05ZYH33	ST7	Human	Blood, PMNs	Attenuated survival, attenuated survival	Blood	Factor	KO	No	NA	No	ABP89966.1	Core
Lun and Willson, 2005	10.1016/j.vetmic.2004.10.017	ManN	Regulation of sly	SS2	SX332	not found	Pig	Erhythrocytes	Increased hemolysis	blood	Regulator	KO	Yes	No	Yes	AY687929.1	Accessory
Lun et al., 2003	10.1016/s0882-4010(02)00192-4	sly	Hemolysin	SS2	31533	ST1	Pig	Monocytes	Increased TNF secretion	blood	Factor	Rec. Protein	NA	NA	no	AY139031.1	Accessory
Ma et al., 2020	10.3390/pathogens9050387	endoSS	N-Glycan degradation	SS2	ZY05719	ST7	Pig	Serum, IgG	Attenuated growth, uncleaved	Blood	Factor	KO and Rec. Protein	Yes	No	Yes	WP_014636140.1	Accessory
Ma et al., 2020	10.3390/pathogens9050387	gh92	Mannosidase	SS2	ZY05719	ST7	Pig	Serum, IgG	Growth unaffected, uncleaved	Blood	Factor	KO and Rec. Protein	Yes	No	Yes	AKG41132.1	Accessory
Meijerink et al., 2012	10.1371/journal.pone.0035849	cps2E+F	CPS biosynthesis	SS2	S10 (strain 10)	ST1	Pig	Dendritic cells, HEK293 TLR2/6 reporter cell line	Increased phagocytosis, not affected intracellular survival, increased NF-kB activation	Blood	Factor	KO	No	NA	No	AAD24451.1 and AAD24452.1	Accessory
Musyoki et al., 2016	10.1073/pnas.1608406113	FBPS	Adhesin: fibronectin and fibrinogen binding	SS2	05ZYH33	ST7	Human	HEp2, Fibronectin	Attenuated adherence, binds	Epithelium, Blood, ECM	Factor	KO	Yes	No	Yes	ABP90458.1	Core
Ni et al., 2020	10.1080/21505594.2020.1792080	htpsA	Histidine triad proteins (regulator)	SS2	05ZYH33	ST7	Human	HEp2, blood	Attenuated adherence, attenuated survival	Epithelium, blood	Regulator	KO	Yes	No	No	ABP89300	Core
Norton et al., 1999	10.1111/j.1574-695X.1999.tb01369.x	sly	Hemolysin	SS14	752	not found	Pig	HEp2	Anti-sly Ab decreased Hep-2 lysis	Epithelium	Factor	blocking	NA	NA	NA	not found	Accessory
Norton et al., 1999	10.1111/j.1574-695X.1999.tb01369.x	sly	Hemolysin	SS2	B831	ST1	unknown	HEp2	Anti-sly Ab decreased Hep-2 lysis	Epithelium	Factor	blocking	NA	NA	NA	not found	Accessory
Norton et al., 1999	10.1111/j.1574-695X.1999.tb01369.x	sly	Hemolysin	SS2	P1/7	ST1	Pig	HEp2	Anti-sly Ab decreased Hep-2 lysis	Epithelium	Factor	blocking	NA	NA	NA	CAR46566.1	Accessory
Pan et al., 2009	10.1128/JB.01309-08	CovR	Orphan response regulator	SS2	05ZYH33	ST7	Human	HEp2, HUVEC, Neutrophils, Monocytes	Increased adherence, increased adherence, increased survival, increased survival	Epithelium, Blood, BBB	Regulator	KO	Yes	Slower	Yes	ABP90698.1	Core
Pian et al., 2015	10.3389/fcimb.2015.00019	Fhb	(Cell envelope assocaited) Factor H/C3b/C3d binding protein	SS2	05ZYH33	ST7	Human	Fibrinogen	Attenuated binding	ECM, Blood	Factor	KO	Yes	No	No	ABP89240.1	Accessory
Pian et al., 2015	10.3389/fcimb.2015.00019	HP1083	Putative ATP-binding casette transporter. Binds human fibrinogen.	SS2	05ZYH33	ST7	Human	Fibrinogen, PMNs, Blood	Attenuated binding, not affected survival, not affected survival	ECM, Blood	Factor	KO	No	NA	No	ABP90049.1	Core
Pian et al., 2015	10.3389/fcimb.2015.00019	HP1538	Putative nucleotidase. Binds human fibrinogen (cell wall protein)	SS2	05ZYH33	ST7	Human	Fibrinogen, PMNs, Blood	Attenuated binding, not affected survival, not affected survival	ECM, Blood	Factor	KO	No	NA	No	ABP90504.1	Core
Pian et al., 2015	10.3389/fcimb.2015.00019	MRP	Muramidase released protein, binds fibrinogen and fibrinogen binding protein (cell wall-anchored)	SS2	05ZYH33	ST7	Human	Fibrinogen, PMNs, Blood	Attenuated binding, attenuated survival , attenuated survival	ECM, Blood	Factor	KO	No	NA	No	ABP89719.1	Accessory
Pian et al., 2015	10.3389/fcimb.2015.00019	Ssads	Converts AMP to adenosine (cell wall-anchored protein)	SS2	05ZYH33	ST7	Human	Fibrinogen	Attenuated binding	ECM, Blood	Factor	KO	No	NA	No	ABP89966.1	Core
Pian et al., 2012	10.1128/IAI.06294-11	Fhb	(Cell envelope assocaited) Factor H/C3b/C3d binding protein	SS2	05ZYH33	ST7	Human	Blood, PMNs, C3b/iC3b, Factor H	Attenuated survival, attenuated survival, attenuated deposition, attenuated deposition	Blood	Factor	KO	Yes	No	Yes	ABP89240.1	Accessory
Pian et al., 2016	10.1038/srep26966	MRP	Muramidase released protein, binds fibrinogen and fibrinogen binding protein (cell wall-anchored)	SS2	05ZYH33	ST7	Human	Blood, PMNs, Fibrinogen, plasma, serum, Fibrinogen	Attenuated survival, Attenuated survival, attenuated survival, attenuated survival,survival not affected, dose dependent binding	Blood, ECM	Factor	KO	No	NA	Yes	ABP89719.1	Accessory
Qian et al., 2018	10.1016/j.micpath.2018.06.008	Sbp1	Putative ancillary pilus subunit. Contributes to adherence.	SS2	ZY05719	ST7	Pig	HEp2, zebrafish	Attenuated adherence not affected invasion, not affected survival	Epithelium, Blood	Factor	KO	Yes	No	No	WP_012027976.1	Accessory
Roy et al., 2016	10.3390/pathogens503004	cps2F	CPS biosynthesis	SS2	P1/7	ST1	Pig	Factor H, C3b, THP-1	Attenuated, not affected cleavage, increased phagocytosis	Blood	Factor	KO	Yes	No	No	CAR45153.1	Accessory
Roy et al., 2016	10.3390/pathogens503004	Fhb	(Cell envelope assocaited) Factor H/C3b/C3d binding protein	SS2	P1/7	ST1	Pig	A549, BMEC, macrophages, Factor H, C3b cleavage	Not affected adherence, not affected adherence, not affected phagocytosis, not affected deposition, not affected cleavage	Epithelium, Blood, BBB	Factor	KO	Yes	No	No	CAR44630.1	Accessory
Roy et al., 2016	10.3390/pathogens503004	Fhb+Fhbp	Double KO of two Factor H binding proteins	SS2	P1/7	ST1	Pig	A549, BMEC, macrophages, Factor H, C3b cleavage	Attenuated adherence, attenuated adherence, attenuated survival, not affected deposition, not affected cleavage	Epithelium, Blood, BBB	Factor	KO	Yes	No	No	CAR44630.1 (fhb) and CAR44487.1 (fhbp)	Accessory and Accessory
Roy et al., 2016	10.3390/pathogens503004	Fhb+Fhbp+CPS 2F	Combined	SS2	P1/7	ST1	Pig	Factor H, C3b cleavage	Attenuated binding, not affected cleavage	Blood	Factor	KO	Yes	No	No	CAR44630.1 (fhb), CAR44487.1 (fhbp) and CAR45153.1 (CPS2F)	Accessory, Accessory and Accessory
Roy et al., 2016	10.3390/pathogens503004	Fhbp	Factor H binding protein	SS2	P1/7	ST1	Pig	A549, BMEC, macrophages, C3b cleavage	Not affected adherence, not affected adherence, not affected phagocytosis, not affected cleavage	Epithelium, Blood, BBB	Factor	KO	Yes	No	Yes	CAR44487.1 (fhbp)	Accessory
Roy et al., 2019	10.1016/j.micres.2019.04.003	ClpP	caseinolytic protease subunit	SS2	ZY05719	ST7	Pig	HEp2	Attenuated adherence, not affected invasion	Epithelium	Regulator	KO	Yes	Slower	Yes	AKG40801.1	Core
Roy et al., 2019	10.1016/j.micres.2019.04.003	ClpX	caseinolytic protease subunit	SS2	ZY05719	ST7	Pig	HEp2	Attenuated adherence, not affected invasion	Epithelium	Regulator	KO	Yes	Slower	Yes	AKG40165.1	Core
Rui et al., 2018	10.1111/cmi.12862	Stk	Serine/threonine protein kinase	SS2	ZY05719	ST7	Pig	BMEC	Attenuated adherence, invasion and crossing of monolayer and attenuated Claudin-5 expression.	BBB	Regulator	KO	Yes	Slower in stationary phase	Yes	AKG39812.1	Core
Salasia et al., 1995	10.1016/0378-1135(95)00036-a	cps	CPS biosynthesis	SS2	89-1591	ST25	Pig	Buccal Mucosa Epithelial cells, HeLa , PMNs	Increased adherence, not affected adherence, increased phagocytosis	Epithelium, Blood	Factor	KO	No	NA	No	not found	Accessory
Schwerk et al., 2012	10.1371/journal.pone.0030069	cps2E+F	CPS biosynthesis	SS2	S10 (strain 10)	ST1	Pig	HIBCPP	Trend in attenuated polar invasion	BBB	Factor	KO	No	NA	No	AAD24451.1 and AAD24452.1	Accessory
Seele et al., 2013	10.1128/JB.01875-12	IdeS/Mac-1	Secreted porcine IgM cleaving enzyme	SS2	S10 (strain 10)	ST1	Pig	IgG, IgM	Not cleaved/affected, not cleaved/affected	Blood	factor	KO and Rec. Protein	No	NA	Yes	CAR45112.1	Accessory
Seitz et al., 2013	10.1016/j.vetmic.2013.09.010	sly	hemolysin	SS2	S10 (strain 10)	ST1	Pig	HEp2	Attenuated invasion, unaffected adherence	Epithelium	Factor	KO	No	NA	Rec. protein	not found	Accessory
Shao et al., 2014	10.1007/s00284-014-0642-4	Sbp2	Truncated major pilin subunit	SS2	P1/7	ST1	Pig	HEp2, Zebrafish	Attenuated adherence, attenuated survival	Epithelium, Blood	Factor	KO	No	NA	Yes	PSEUDO:CAR47578.1 (SSU1886)	Accessory

Si et al., 2009	10.1016/j.vetmic.2009.04.024	GlnA	Glutamine synthetase (nitrogen metabolism)	SS2	SC-19	ST7	Pig	HEp2	Attenuated adherence, not affected invasion	Epithelium	Factor	KO	Yes	No	Yes	ABP89130.1	Core
Spoerry et al., 2016	10.1074/jbc.M115.711440	IgdE	IgG degrading enzyme (Cysteine protease)	SS2	S10 (strain 10)	ST1	Pig	IgG	Not cleaved/affected	Blood	Factor	KO	No	NA	Rec. protein	YP_003029385.1	Accessory
Tan et al., 2011	10.1016/j.micpath.2011.07.008	SsPep/SSPepO	Metallo-endopeptidase (extracellular protein) binding fibronectin	SS2	SC-19	ST7	Pig	HEp2	Attenuated adherence	Epithelium	Factor	KO	No	NA	Yes	ABP89124.1	Accessory
Tan et al., 2017	10.1002/mbo3.433	MsmK	ATPase of ABC transporters.	SS2	SC-19	ST7	Pig	HEp2	Increased adhesion	Epithelium	Regulator	KO	No	NA	Yes	CAZ52500.1 in sc84	Core
Vadeboncoeur et al., 2003	0.1111/j.1574-695X.2003.tb00648.x	cps	CPS biosynthesis	SS2	S735	ST1	Pig	BMEC	Increased IL-6, IL-8 and decreased MCP-1	BBB	Factor	KO	No	NA	No	not found	Accessory
Wang et al., 2009	10.1007/s00203-008-0425-z	srtA	sortase A, covalent linkage of surface proteins to the peptidoglycan, such as MRP and Sao	SS2	05ZYH33	ST7	Human	HEp2, HUVEC	Attenuated adherence, attenuated adherence	Epithelium, Blood, BBB	Factor	KO	Yes	No	No	ABP90040.1	Core
Wang et al., 2015	10.3389/fmicb.2015.01001	MRP	Muramidase released protein, binds fibrinogen and fibrinogen binding protein (cell wall-anchored)	SS2	05ZYH33	ST7	Human	hCMEC/D3 cells with Fibrinogen	Attenuated adhesion, attenuated transversion, attenuated permeability and attenuated p120-catenin degradation (all in presence of fibrinogen)	BBB	factor	KO	No	NA	No	ABP89719.1	Accessory
Wang et al., 2017	10.1111/cmi.12724	SP1	PPIase molecule, interacts with peptidoglycan recognition protein (PGLYRP-1)	SS2	05ZYH33	ST7	Human	PGLYRP-2	Attenuated survival	Blood	Factor	KO	Yes	No	Rec. protein	ABP89908.1	Accessory
Wang et al., 2017	10.1111/cmi.12724	SP1	PPIase molecule, interacts with peptidoglycan recognition protein (PGLYRP-1)	SS2	BM407	ST1	Human	PGLYRP-1	Attenuated survival	Blood	Factor	KO	Yes	No	Rec. protein	YP_003028706.1	Accessory
Wang et al., 2014	10.1007/s00284-013-0509-0	AI-2	Quorum sensing (LuxS based)	SS2	HA9801	ST378	Pig	HEp2	AI-2 dependent effect on adhesion	Epithelium	Regulator	KO	No	NA	Yes	WP_004194159.1	ND
Wang et al., 2011	10.1016/j.vetmic.2011.04.029	LuxS	Involved in AI-2 quorum sensing	SS2	HA9801	ST378	Pig	HEp2, Zebrafish	Attenuated adherence, not affected invasion	Epithelium, Blood	Regulator	KO	No	NA	No	WP_004194159.1	Core
Willenborg et al., 2016	10.3390/pathogens5030051	FlpS	Oxygen-sensing activator of the arginine deiminase system, involved in arcABC operon expression	SS2	S10 (strain 10)	ST1	Pig	HEp2	Attenuated intracellular survival	Epithelium	Regulator	KO	Yes	Slower	Yes	KCJCKLJI_00603	Core
Wu et al., 2009	10.1111/j.1574-6968.2008.01486.x.	RevSC21	transcriptional regulator	SS2	SC21	ST7	Human	HEp2	Attenuated	Epithelium	Regulator	KO	Yes	No	Yes	not found	NA
Wu et al., 2014	10.1261/rna.041822.113	rss03, rss04, rss05, rss06 and rss11	Transriptional regulator	SS2	P1/7	ST1	Pig	Zebrafish	Attenuated survival for al rss	Blood	Regulator	KO	No	NA	No	NA	NA
Xiao et al., 2017	10.1007/s00284-016-1160-3	IdeS/Mac-1	Secreted porcine IgM cleaving enzyme	SS2	P1/7	ST1	Pig	Zebrafish	Not affected survival	Blood	Factor	KO	No	NA	Yes	CAR45112.1	Accessory
Xie et al., 2019	10.1074/jbc.RA119.009441	abpb/ApdS	Surface-associated arginine peptidase/Cysteine protease	SS2	05ZYH33	ST7	Human	Neutrophils, LL-37, Blood, THP-1, NETs	Attenuated chemotaxis and survival, increased susceptability to LL37, attenuated survival, attenuated survival, attenuated survival	Blood	Factor	KO	Yes	No	Yes	ABP90353.1	Accessory
Xu et al., 2014	10.1016/j.micres.2013.11.002	NisK+NisR	Two-component system	SS2	SC-19	ST7	Pig	HeLa	Attenuated adhesion and invasion	Epithelium	Regulator	KO	Yes	No	No	CAZ51589.1 (NisR) and CAZ51588.1 (NisK)	Accessory and Accessory
Yi et al., 2020	10.1016/j.vetmic.2020.108925	otc	catalyzes ornithine and carbamoyl phosphate in citrulline	SS2	ZY05719	ST7	Pig	HEp2	Attenuated adherence	Epithelium	Factor	KO	Yes	No	Yes	WP_000195397.1	Core
Yu et al., 2015	10.1074/jbc.M115.643338	SsTGase (IgdE)	Secreted transglutaminase	SS2	05ZYH33	ST7	Human	Blood, PMNs	Attenuated survival, Attenuated survival	Blood	Factor	KO	No	NA	Yes	ABP90781.1	Accessory
Yu et al., 2016	10.1039/c6mb00059b	pepT	Peptidase	SS2	ZY05719	ST7	Pig	Zebrafish	Attenuated survival	Blood	Factor	KO	Yes	No	No	AKG40425.1	Core
Yu et al., 2016	10.1039/c6mb00059b	rfeA	Pore-forming toxin	SS2	ZY05719	ST7	Pig	Zebrafish	KO has increased lethality	Blood	Factor	KO	Yes	No	No	AKG39612.1	Accessory
Yu et al., 2016	10.1039/c6mb00059b	Sbp2	putative pilus protein	SS2	ZY05719	ST7	Pig	HEp2	Rec. protein adheres to Hep-2 cells and decreases ZY05719 binding	Epithelium	Factor	Rec. Protein	NA	NA	NA	ABP91068.1	Accessory
Yu et al., 2018	10.1016/j.jprot.2017.12.001	adhE	acetaldehyde-CoA/alcohol dehydrogenase (binds to Hsp60)	SS2	ZY05719	ST7	Pig	Zebrafish, Caco-2, HEp2	Attenuated, radhE binds to Caco-2 and HEp2- cells, attenuated adherence to Caco-2	Epithelium, Blood	Factor	KO	Yes	NA	Rec. protein	AKG39693.1	Core
Yuan et al., 2017	10.1016/j.micpath.2016.12.026	1910HK+RR	Two-component system signal transduction system	SS2	05ZYH33	ST7	Human	HEp2, Blood	Attenuated adherence and invasion, attenuated survival	Epithelium, Blood	Regulator	KO	Yes	No	Yes	ABP90877.1 (1910HK) and ABP90876.1 (1910HR)	Accessory
Yuan et al., 2013	10.1074/jbc.M112.388686	HP0197 (Fhbp)	Glycosaminoglycan binding protein	SS2	SC-19	ST7	Pig	HEp2	Rec. protein attenuates adherence to Hep-2	Epithelium, Blood	Factor	Rec. Protein	NA	NA	NA	WP_011922585.1	Accessory
Zaccaria et al., 2016	10.1371/journal.pone.0151623	ciaRH	Two-component system signal transduction system	SS2	S10 (strain 10)	ST1	Pig	Zebrafish larvae	Attenuated survival	Blood	Regulator	KO	Yes	Slower	No	ABP90060.1 (ciaH) and ABP90061.1 (ciaR)	ciaH Accessory ciaR Core
Zaccaria et al., 2016	10.1371/journal.pone.0151623	cps2E+F	CPS biosynthesis	SS2	S10 (strain 10)	ST1	Pig	Zebrafish larvae	Attenuated survival	Blood	Factor	KO	No	NA	NA	AAD24451.1 and AAD24452.1	Accessory
Zhang et al., 2015	10.1002/mbo3.307	AgaR2	Regulator of the GalN/GalNAc catabolism pathway	SS2	05ZYH33	ST7	Human	HEp2	Increased adherence	Epithelium, Blood	Regulator	KO	Yes	No	Yes	ABP89413.1	Accessory
Zhang et al., 2010	10.1007/s00284-010-9643-0	trag	potential Type IV secretion system	SS2	HA9801	ST378	Pig	Zebrafish larvae	Attenuated survival	Blood	Factor	KO	No	NA	No	ABS32277.1	Accessory
H. Zhang et al., 2014	10.1007/s12275-014-4311-x	EF-G	translation elongation factor G	SS2	HA9801	ST378	Pig	Collagen, Fibronectin	Dose dependent binding, dose dependent binding	ECM, Blood	Factor	Rec. Protein	NA	NA	NA	ZP_03625772.1	Core
H. Zhang et al., 2014	10.1007/s12275-014-4311-x	GroEL	Chaperonin	SS2	HA9801	ST378	Pig	Collagen, Fibronectin	No binding, dose dependent binding	ECM, Blood	Regulator	Rec. Protein	NA	NA	NA	WP_012774912.1	Core
H. Zhang et al., 2014	10.1007/s12275-014-4311-x	OppA	Surface associated oligopeptide binding protein	SS2	HA9801	ST378	Pig	Collagen, Fibronectin	Dose dependent binding, dose dependent binding	ECM, Blood	Factor	Rec. Protein	NA	NA	NA	WP_012775331.1	Accessory
H. Zhang et al., 2014	10.1007/s12275-014-4311-x	PDH	pyruvate dehydrogenase E1 component, alpha subunit	SS2	HA9801	ST378	Pig	Collagen, Fibronectin	No binding, dose dependent binding	ECM, Blood	Factor	Rec. Protein	NA	NA	NA	WP_012775325.1	Core
H. Zhang et al., 2014	10.1007/s12275-014-4311-x	PGK	phosphoglycerate kinase	SS2	HA9801	ST378	Pig	Collagen, Fibronectin	No binding, dose dependent binding	ECM, Blood	Factor	Rec. Protein	NA	NA	NA	WP_011921748.1	Core
H. Zhang et al., 2014	10.1007/s12275-014-4311-x	PGM	phosphoglycerate mutase	SS2	HA9801	ST378	Pig	Collagen, Fibronectin	Dose dependent binding, dose dependent binding	ECM, Blood	Factor	Rec. Protein	NA	NA	NA	WP_012027561.1	Core
Zhang et al., 2013	10.1007/s00284-010-9643-0	cbp40	Collagen binding protein	SS2	ZY05719	ST7	Pig	HEp2, zebrafish	Attenuated adherence, attenuated survival	Epithelium, Blood	Factor	KO	No	NA	Yes	AKG41298.1	Accessory
S. Zhang et al., 2016	10.1038/srep36787	sly	Hemolysin	SS2	05ZYH33	ST7	Human	Blood	Attenuated p-selectin depending platelet-neutrophil complex (PNC) formation in culture supernatant, casued by Ca2+ inlfux in platelets by Sly	blood	Factor	Rec. Protein	NA	NA	no	ABP90369.1	Accessory
Zhang et al., 2011	10.1371/journal.pone.0021234	ABC	Amino acid ABC transporter permease	SS2	HA9801	ST378	Pig	HEp2	Rec. protein binds	Epithelium	Factor	Rec. Protein	NA	NA	NA	WP_012027948.1	Accessory
Zhang et al., 2011	10.1371/journal.pone.0021234	MRP	Muramidase released protein, binds fibrinogen and fibrinogen binding protein (cell wall-anchored)	SS2	HA9801	ST378	Pig	HEp2	Rec. protein binds	Epithelium	Factor	Rec. Protein	NA	NA	NA	WP_012775059.1	Accessory
X. Zhang et al., 2014	10.4014/jmb.1408.08085	DnaJ	Chaperone protein/Surface associated adhesing protein	SS2	HA9801	ST378	Pig	HEp2	Attenuated adherence	Epithelium	Regulator/ Factor	Partial KO	Yes_NA	No	NA	CAR44688.1	Core
X. Zhang et al., 2014	10.4014/jmb.1408.08085	DnaK	Chaperone protein/Surface associated adhesing protein	SS2	HA9801	ST378	Pig	HEp2	Attenuated adherence	Epithelium	Regulator/ Factor	Partial KO	Yes_NA	No	NA	CAR44686.1	Core
X. Zhang et al., 2014	10.4014/jmb.1408.08085	GrpE	Chaperone protein (regulation)	SS2	HA9801	ST378	Pig	HEp2	Not affected adherence	Epithelium	Regulator	Partial KO	Yes_NA	No	NA	CAR44684.1	Core
X. Zhang et al., 2014	10.4014/jmb.1408.08085	HrcA	Chaperone protein (regulation)	SS2	HA9801	ST378	Pig	HEp2	Not affected adherence	Epithelium	Regulator	Partial KO	Yes_NA	No	NA	CAR44681.1	Core
Y. Zhang et al., 2016	10.1016/j.micres.2016.02.002	cps2E	CPS biosynthesis	SS2	SC-19	ST7	Pig	HEp2	Increased adherence and invasion	Epithelium	Factor	KO	No	NA	No	ABP89534.1	Accessory
Y. Zhang et al., 2016	10.1016/j.micres.2016.02.002	cps2G	CPS biosynthesis	SS2	SC-19	ST7	Pig	HEp2	Increased adherence and invasion	Epithelium	Factor	KO	No	NA	No	ABP89536.1	Accessory
Y. Zhang et al., 2016	10.1016/j.micres.2016.02.002	cps2J	CPS biosynthesis	SS2	SC-19	ST7	Pig	HEp2	Increased adherence and invasion	Epithelium	Factor	KO	No	NA	No	ABP89539.1	Accessory
Y. Zhang et al., 2016	10.1016/j.micres.2016.02.002	cps2L	CPS biosynthesis	SS2	SC-19	ST7	Pig	HEp2	Increased adherence and invasion	Epithelium	Factor	KO	No	NA	No	ABP89541.1	Accessory
Zhang et al., 2018	10.1128/AEM.01385-18	SssP1	Forms fimbria-like structures that extend outward from the bacterial surface	SS2	CZ130302	not found	Pig	HEp2, BMEC	Attenuated adherence, attenuated adherence	Epithelium, BBB	Factor	KO	Yes	No	No	AWD32147.1	Not found
Zhang et al., 2019	10.1016/j.vetmic.2019.05.020	AtlAss	Autolysin	SS2	CZ130302	not found	Pig	Fibrinogen, Fibronectin, BMEC	Attenuated adhesion, attenuated adhesion, attenuated adherence	Blood, ECM, BBB	Factor	KO	Yes	No	Yes	ATZ02642.1	Accessory

Zhao et al., 2020	10.1080/21505594.2020.1797352	Ssads	Converts AMP to adenosine (cell wall-anchored protein)	SS2	05ZYH33	ST7	Human	hCMEC/D3, hCMEC/D3 A1 AR KO	Not affected adhesion and invasion but attenuated translocation and monolayer permeability, translocation Ssads KO not affected but WT attenuated translocation in A1 AR KO cells compared to WT	BBB	Factor	KO	Yes	No	Chemical complementation	ABP89966.1	Core
Zheng et al., 2011	10.1128/IAI.00193-10	Rgg (TR)	Trancriptional regulator	SS2	05ZYH33	ST7	Human	HEp2, Erythrocytes	Increased adherence, increased hemolysis	Epithelium, Blood	Regulator	KO	Yes	Slower	Yes	ABP90963.1	Accessory
Zheng et al., 2018	10.1016/j.micpath.2018.03.061	OppA	Surface associated oligopeptide binding protein	SS2	05ZYH33	ST7	Human	HEp2	Attenuated afherence	Epithelium	Factor	KO	Yes	Slower	No	AIC32936.1	Accessory
Zheng et al., 2013	10.1371/journal.pone.0074674	Hhly3	Hemolysin	SS2	ZY05719	ST7	Pig	Zebrafish	Attenuated survival	Blood	Factor	KO	No	NA	Yes	AKG40249.1	Accessory
Zhou et al., 2014	10.1007/s00284-014-0527-6	IMPDH	Inosine 5′-monophosphate dehydrogenase	SS2	ZY05719	ST7	Pig	HEp2	Attenuated adherence	Epithelium	Regulator/ Factor	KO	Yes	No	No	DQ097893.1	Core
Zhou et al., 2018	10.1093/femsle/fny091	GntR (P1/7)	Transcriptional regulator	SS2	P1/7	ST1	Pig	Blood	Not affected survival	Blood	Regulator	KO	Yes	No	Yes	CAR45142.1	Core
Zhou et al., 2018	10.1093/femsle/fny091	GntR (SC070731)	Transcriptional regulator	SS2	P1/7	ST1	Pig	Blood	Increased survival	Blood	Regulator	KO	Yes	No	Yes	AGG64002.1	Core
Zhu et al., 2011	10.1016/j.micpath.2011.08.002	Stp	Serine/threonine protein phosphatase	SS9	GZ0565	ST243	Pig	HEp2	Attenuated adherence	Epithelium	Regulator	KO	Yes	No	Yes	GU073258.1	Core
Zhu et al., 2014	10.1371/journal.pone.0091971	Stk	Serine/threonine protein kinase	SS2	SS2-1	ST7	Pig	HEp2	Attenuated adherence	Epithelium	Regulator	KO	Yes	No	Yes	AUW21374.1	Core
Zhu et al., 2018	10.3389/fcimb.2018.00317	Rex	Transcriptional regulator	SS2	SS2-1	ST7	Pig	HEp2	Not affected adherence and invasion	Epithelium	Regulator	KO	No	NA	Yes	AUW21934.1	Core
Zhu et al., 2019	10.1016/j.micres.2019.04.001	codY	Transcriptional regulator	SS2	SC-19	ST7	Pig	HEp2	Attenuated adherence and invasion	Epithelium	Regulator	KO	Yes	Slower	No	ABP89317.1	Core
Zhu et al., 2019	10.1016/j.micres.2019.04.001	RelA+relQ	Central mediator of stress response	SS2	SC-19	ST7	Pig	HEp2	Attenuated adherence and invasion, attenuated colonization	Epithelium	Regulator	KO	Yes	Slower	No	ABP91060.1 (rela) ABP90026.1 (relq) (based on 05ZYH33 genome)	Core and Core
Zhu et al., 2019	10.1016/j.micres.2019.04.001	RelA+relQ+codY	Central mediator of stress response and DNA binding repressor	SS2	SC-19	ST7	Pig	HEp2	Attenuated adherence and invasion	Epithelium	Regulator	KO	Yes	Slower	No	ABP91060.1 (rela) ABP90026.1 (relq) (codY) (based on 05ZYH33 genome)	Core, Core and Core
Zhu et al., 2016	10.1016/j.micres.2016.05.007	RelA+relQ	Central mediator of stress response	SS2	SC-19	ST7	Pig	HEp2, THP-1	Attenuated adherence and invasion, increased phagocytosis	Epithelium, Blood	Regulator	KO	Yes	Slower	No	ABP91060.1 (rela) ABP90026.1 (relq) (based on 05ZYH33 genome)	Core and Core

References

Auger, J.P., Christodoulides, M., Segura, M., Xu, J., Gottschalk, M., 2015. Interactions of Streptococcus suis serotype 2 with human meningeal cells and astrocytes. BMC Res Notes 8, 607. https://doi.org/10.1186/s13104-015-1581-2
Auger, J.P., Chuzeville, S., Roy, D., Mathieu-Denoncourt, A., Xu, J., Grenier, D., Gottschalk, M., 2017. The bias of experimental design, including strain background, in the determination of critical Streptococcus suis serotype 2 virulence factors. PLoS One 12, e0181920. https://doi.org/10.1371/journal.pone.0181920
Bai, Q., Ma, J., Zhang, Z., Zhong, X., Pan, Z., Zhu, Y., Zhang, Y., Wu, Z., Liu, G., Yao, H., 2020. YSIRK-G/S-directed translocation is required for Streptococcus suis to deliver diverse cell wall anchoring effectors contributing to bacterial pathogenicity. Virulence 11, 1539–1556. https://doi.org/10.1080/21505594.2020.1838740
Benga, L., Goethe, R., Rohde, M., Valentin-Weigand, P., 2004. Non-encapsulated strains reveal novel insights in invasion and survival of Streptococcus suis in epithelial cells. Cell Microbiol 6, 867–881. https://doi.org/10.1111/j.1462-5822.2004.00409.x
Bonifait, L., de la Cruz Dominguez-Punaro, M., Vaillancourt, K., Bart, C., Slater, J., Frenette, M., Gottschalk, M., Grenier, D., 2010. The cell envelope subtilisin-like proteinase is a virulence determinant for Streptococcus suis. BMC Microbiol 10, 42. https://doi.org/10.1186/1471-2180-10-42
Bonifait, L., Grenier, D., 2011. The SspA subtilisin-like protease of Streptococcus suis triggers a pro-inflammatory response in macrophages through a non-proteolytic mechanism. BMC Microbiol 11, 47. https://doi.org/10.1186/1471-2180-11-47
Bonifait, L., Vaillancourt, K., Gottschalk, M., Frenette, M., Grenier, D., 2011. Purification and characterization of the subtilisin-like protease of Streptococcus suis that contributes to its virulence. Vet Microbiol 148, 333–340. https://doi.org/10.1016/j.vetmic.2010.09.024
Cao, M., Feng, Y., Wang, C., Zheng, F., Li, M., Liao, H., Mao, Y., Pan, X., Wang, J., Hu, D., Hu, F., Tang, J., 2011. Functional definition of LuxS, an autoinducer-2 (AI-2) synthase and its role in full virulence of Streptococcus suis serotype 2. J Microbiol 49, 1000–1011. https://doi.org/10.1007/s12275-011-1523-1
Chang, P., Li, W., Shi, G., Li, H., Yang, X., Xia, Z., Ren, Y., Li, Z., Chen, H., Bei, W., Velikova, N., 2018. The VraSR regulatory system contributes to virulence in Streptococcus suis via resistance to innate immune defenses. Virulence 9, 771–782. https://doi.org/10.1080/21505594.2018.1428519
Charland, N., Nizet, V., Rubens, C.E., Kim, K.S., Lacouture, S., Gottschalk, M., 2000. Streptococcus suis serotype 2 interactions with human brain microvascular endothelial cells. Infect Immun 68, 637–643. https://doi.org/10.1128/iai.68.2.637-643.2000
Chen, B., Zhang, A., Xu, Z., Li, R., Chen, H., Jin, M., 2011. Large-scale identification of bacteria-host crosstalk by affinity chromatography: capturing the interactions of Streptococcus suis proteins with host cells. J Proteome Res 10, 5163–5174. https://doi.org/10.1021/pr200758q
Chen, S., Xie, W., Wu, K., Li, P., Ren, Z., Li, L., Yuan, Y., Zhang, C., Zheng, Y., Lv, Q., Jiang, H., Jiang, Y., 2016. Suilysin Stimulates the Release of Heparin Binding Protein from Neutrophils and Increases Vascular Permeability in Mice. Front Microbiol 7, 1338. https://doi.org/10.3389/fmicb.2016.01338
de Buhr, N., Neumann, A., Jerjomiceva, N., von Kockritz-Blickwede, M., Baums, C.G., 2014. Streptococcus suis DNase SsnA contributes to degradation of neutrophil extracellular traps (NETs) and evasion of NET-mediated antimicrobial activity. Microbiology 160, 385–395. https://doi.org/10.1099/mic.0.072199-0
de Buhr, N., Stehr, M., Neumann, A., Naim, H.Y., Valentin-Weigand, P., von Kockritz-Blickwede, M., Baums, C.G., 2015. Identification of a novel DNase of Streptococcus suis (EndAsuis) important for neutrophil extracellular trap degradation during exponential growth. Microbiology 161, 838–850. https://doi.org/10.1099/mic.0.000040
Deng, S., Xu, T., Fang, Q., Yu, L., Zhu, J., Chen, L., Liu, J., Zhou, R., 2018. The Surface-Exposed Protein SntA Contributes to Complement Evasion in Zoonotic Streptococcus suis. Front Immunol 9, 1063. https://doi.org/10.3389/fimmu.2018.01063
Du, B., Ji, W., An, H., Shi, Y., Huang, Q., Cheng, Y., Fu, Q., Wang, H., Yan, Y., Sun, J., 2014. Functional analysis of c-di-AMP phosphodiesterase, GdpP, in Streptococcus suis serotype 2. Microbiol Res 169, 749–758. https://doi.org/10.1016/j.micres.2014.01.002
Dumesnil, A., Auger, J.P., Roy, D., Votsch, D., Willenborg, M., Valentin-Weigand, P., Park, P.W., Grenier, D., Fittipaldi, N., Harel, J., Gottschalk, M., 2018. Characterization of the zinc metalloprotease of Streptococcus suis serotype 2. Vet Res 49, 109. https://doi.org/10.1186/s13567-018-0606-y
Esgleas, M., Lacouture, S., Gottschalk, M., 2005. Streptococcus suis serotype 2 binding to extracellular matrix proteins. FEMS Microbiol Lett 244, 33–40. https://doi.org/10.1016/j.femsle.2005.01.017
Fang, L., Zhou, J., Fan, P., Yang, Y., Shen, H., Fang, W., 2017. A serine/threonine phosphatase 1 of Streptococcus suis type 2 is an important virulence factor. J Vet Sci 18, 439–447. https://doi.org/10.4142/jvs.2017.18.4.439
Feng, L., Zhu, J., Chang, H., Gao, X., Gao, C., Wei, X., Yuan, F., Bei, W., 2016. The CodY regulator is essential for virulence in Streptococcus suis serotype 2. Sci Rep 6, 21241. https://doi.org/10.1038/srep21241
Feng, Y., Cao, M., Shi, J., Zhang, H., Hu, D., Zhu, J., Zhang, X., Geng, M., Zheng, F., Pan, X., Li, X., Hu, F., Tang, J., Wang, C., 2012. Attenuation of Streptococcus suis virulence by the alteration of bacterial surface architecture. Sci Rep 2, 710. https://doi.org/10.1038/srep00710
Feng, Y., Pan, X., Sun, W., Wang, C., Zhang, H., Li, X., Ma, Y., Shao, Z., Ge, J., Zheng, F., Gao, G.F., Tang, J., 2009. Streptococcus suis enolase functions as a protective antigen displayed on the bacterial cell surface. J Infect Dis 200, 1583–1592. https://doi.org/10.1086/644602
Ferrando, M.L., de Greeff, A., van Rooijen, W.J., Stockhofe-Zurwieden, N., Nielsen, J., Wichgers Schreur, P.J., Pannekoek, Y., Heuvelink, A., van der Ende, A., Smith, H., Schultsz, C., 2015. Host-pathogen Interaction at the Intestinal Mucosa Correlates With Zoonotic Potential of Streptococcus suis. J Infect Dis 212, 95–105. https://doi.org/10.1093/infdis/jiu813
Ferrando, M.L., Willemse, N., Zaccaria, E., Pannekoek, Y., van der Ende, A., Schultsz, C., 2017. Streptococcal Adhesin P (SadP) contributes to Streptococcus suis adhesion to the human intestinal epithelium. PLoS One 12, e0175639. https://doi.org/10.1371/journal.pone.0175639
Fulde, M., Willenborg, J., De Greeff, A., Benga, L., Smith, H.E., Valentin-Weigand, P., Goethe, R., 2011. ArgR is an essential local transcriptional regulator of the arcABC operon in Streptococcus suis and is crucial for biological fitness in an acidic environment. Microbiology 157, 572–582. https://doi.org/10.1099/mic.0.043067-0
Fulde, M., Willenborg, J., Huber, C., Hitzmann, A., Willms, D., Seitz, M., Eisenreich, W., Valentin-Weigand, P., Goethe, R., 2014. The arginine-ornithine antiporter ArcD contributes to biological fitness of Streptococcus suis. Front Cell Infect Microbiol 4, 107. https://doi.org/10.3389/fcimb.2014.00107
Gao, T., Tan, M., Liu, W., Zhang, C., Zhang, T., Zheng, L., Zhu, J., Li, L., Zhou, R., 2016. GidA, a tRNA Modification Enzyme, Contributes to the Growth, and Virulence of Streptococcus suis Serotype 2. Front Cell Infect Microbiol 6, 44. https://doi.org/10.3389/fcimb.2016.00044
Gao, T., Yuan, F., Liu, Z., Liu, W., Zhou, D., Yang, K., Duan, Z., Guo, R., Liang, W., Hu, Q., Tian, Y., Zhou, R., 2019. MnmE, a central tRNA-modifying GTPase, is essential for the growth, pathogenicity, and arginine metabolism of streptococcus suis serotype 2. Front. Cell. Infect. Microbiol. 9. https://doi.org/10.3389/fcimb.2019.00173
Ge, J., Feng, Y., Ji, H., Zhang, H., Zheng, F., Wang, C., Yin, Z., Pan, X., Tang, J., 2009. Inactivation of dipeptidyl peptidase IV attenuates the virulence of Streptococcus suis serotype 2 that causes streptococcal toxic shock syndrome. Curr Microbiol 59, 248–255. https://doi.org/10.1007/s00284-009-9425-8
Gong, X., Zhuge, Y., Ding, C., Zheng, F., Guo, X., Zhang, Q., Ye, F., Wang, C., Deng, X., 2019. A novel small RNA contributes to restrain cellular chain length and anti-phagocytic ability in Streptococcus suis 2. Microb. Pathog. 137, 103730. https://doi.org/10.1016/j.micpath.2019.103730
Graveline, R., Segura, M., Radzioch, D., Gottschalk, M., 2007. TLR2-dependent recognition of Streptococcus suis is modulated by the presence of capsular polysaccharide which modifies macrophage responsiveness. Int Immunol 19, 375–389. https://doi.org/10.1093/intimm/dxm003
Grenier, D., Bodet, C., 2008. Streptococcus suis stimulates ICAM-1 shedding from microvascular endothelial cells. FEMS Immunol Med Microbiol 54, 271–276. https://doi.org/10.1111/j.1574-695X.2008.00476.x
Haas, B., Bonifait, L., Vaillancourt, K., Charette, S.J., Gottschalk, M., Grenier, D., 2014. Characterization of DNase activity and gene in Streptococcus suis and evidence for a role as virulence factor. BMC Res. Notes 7. https://doi.org/10.1186/1756-0500-7-424
Haas, B., Vaillancourt, K., Bonifait, L., Gottschalk, M., Grenier, D., 2015. Hyaluronate lyase activity of Streptococcus suis serotype 2 and modulatory effects of hyaluronic acid on the bacterium's virulence properties. BMC Res Notes 8, 722. https://doi.org/10.1186/s13104-015-1692-9
Han, H., Liu, C., Wang, Q., Xuan, C., Zheng, B., Tang, J., Yan, J., Zhang, J., Li, M., Cheng, H., Lu, G., Gao, G.F., 2012. The two-component system Ihk/Irr contributes to the virulence of Streptococcus suis serotype 2 strain 05ZYH33 through alteration of the bacterial cell metabolism. Microbiology 158, 1852–1866. https://doi.org/10.1099/mic.0.057448-0
Hu, D., Zhang, F., Zhang, H., Hao, L., Gong, X., Geng, M., Cao, M., Zheng, F., Zhu, J., Pan, X., Tang, J., Feng, Y., Wang, C., 2014. The beta-galactosidase (BgaC) of the zoonotic pathogen Streptococcus suis is a surface protein without the involvement of bacterial virulence. Sci Rep 4, 4140. https://doi.org/10.1038/srep04140
Huang, K., Yuan, Z., Li, J., Zhang, Q., Xu, Z., Yan, S., Zhang, A., Jin, M., 2015. Identification and characterisation a surface-associated arginine peptidase in Streptococcus suis serotype 2. Microbiol Res 170, 168–176. https://doi.org/10.1016/j.micres.2014.08.001
Huang, W., Chen, Y., Li, Q., Jiang, H., Lv, Q., Zheng, Y., Han, X., Kong, D., Liu, P., Jiang, Y., 2021. LytR plays a role in normal septum formation and contributes to full virulence in Streptococcus suis. Vet. Microbiol. 254. https://doi.org/10.1016/j.vetmic.2021.109003
Jiang, H., Wu, T., Liu, J., Yu, X., Liu, H., Bao, C., Liu, M., Ji, Y., Feng, X., Gu, J., Li, N., Lei, L., 2021. Caveolae/rafts protect human cerebral microvascular endothelial cells from Streptococcus suis serotype 2 α-enolase-mediated injury. Vet. Microbiol. 254. https://doi.org/10.1016/j.vetmic.2021.108981
Jiang, X., Yang, Y., Zhou, J., Zhu, L., Gu, Y., Zhang, X., Li, X., Fang, W., 2016. Roles of the Putative Type IV-like Secretion System Key Component VirD4 and PrsA in Pathogenesis of Streptococcus suis Type 2. Front Cell Infect Microbiol 6, 172. https://doi.org/10.3389/fcimb.2016.00172
Jobin, M.C., Brassard, J., Quessy, S., Gottschalk, M., Grenier, D., 2004. Acquisition of host plasmin activity by the Swine pathogen Streptococcus suis serotype 2. Infect Immun 72, 606–610. https://doi.org/10.1128/iai.72.1.606-610.2004
Jobin, M.C., Fortin, J., Willson, P.J., Gottschalk, M., Grenier, D., 2005. Acquisition of plasmin activity and induction of arachidonic acid release by Streptococcus suis in contact with human brain microvascular endothelial cells. FEMS Microbiol Lett 252, 105–111. https://doi.org/10.1016/j.femsle.2005.08.044
Jobin, M.C., Gottschalk, M., Grenier, D., 2006. Upregulation of prostaglandin E2 and matrix metalloproteinase 9 production by human macrophage-like cells: synergistic effect of capsular material and cell wall from Streptococcus suis. Microb Pathog 40, 29–34. https://doi.org/10.1016/j.micpath.2005.10.003
Ju, C.X., Gu, H.W., Lu, C.P., 2012. Characterization and functional analysis of atl, a novel gene encoding autolysin in Streptococcus suis. J Bacteriol 194, 1464–1473. https://doi.org/10.1128/JB.06231-11
Kong, D., Chen, Z., Wang, J., Lv, Q., Jiang, H., Zheng, Y., Xu, M., Zhou, X., Hao, H., Jiang, Y., 2017. Interaction of factor H-binding protein of Streptococcus suis with globotriaosylceramide promotes the development of meningitis. Virulence 8, 1290–1302. https://doi.org/10.1080/21505594.2017.1317426
Lalonde, M., Segura, M., Lacouture, S., Gottschalk, M., 2000. Interactions between Streptococcus suis serotype 2 and different epithelial cell lines. Microbiology-Sgm 146, 1913–1921. https://doi.org/Doi 10.1099/00221287-146-8-1913
LeBel, G., Vaillancourt, K., Yi, L., Gottschalk, M., Grenier, D., 2018. Dipeptidylpeptidase IV of Streptococcus suis degrades the porcine antimicrobial peptide PR-39 and neutralizes its biological properties. Microb Pathog 122, 200–206. https://doi.org/10.1016/j.micpath.2018.06.029
Li, J., Tan, C., Zhou, Y., Fu, S., Hu, L., Hu, J., Chen, H., Bei, W., 2011. The two-component regulatory system CiaRH contributes to the virulence of Streptococcus suis 2. Vet Microbiol 148, 99–104. https://doi.org/10.1016/j.vetmic.2010.08.005
Li, M., Cai, R.J., Li, C.L., Song, S., Li, Y., Jiang, Z.Y., Yang, D.X., 2017. Deletion of ssnA Attenuates the Pathogenicity of Streptococcus suis and Confers Protection against Serovar 2 Strain Challenge. PLoS One 12, e0169791. https://doi.org/10.1371/journal.pone.0169791
Li, M., Shao, Z.Q., Guo, Y., Wang, L., Hou, T., Hu, D., Zheng, F., Tang, J., Wang, C., Feng, Y., Gao, J., Pan, X., 2015. The type II histidine triad protein HtpsC is a novel adhesion with the involvement of Streptococcus suis virulence. Virulence 6, 631–641. https://doi.org/10.1080/21505594.2015.1056971
Li, Q., Fu, Y., He, Y., Zhang, Y., Qian, Y., Yu, Y., Yao, H., Lu, C., Zhang, W., 2017a. Fibronectin-/fibrinogen-binding protein (FBPS) is not a critical virulence factor for the Streptococcus suis serotype 2 strain ZY05719. Vet Microbiol 208, 38–46. https://doi.org/10.1016/j.vetmic.2017.07.010
Li, Q., Fu, Y., Ma, C., He, Y., Yu, Y., Du, D., Yao, H., Lu, C., Zhang, W., 2017b. The non-conserved region of MRP is involved in the virulence of Streptococcus suis serotype 2. Virulence 8, 1274–1289. https://doi.org/10.1080/21505594.2017.1313373
Li, Q., Liu, H., Du, D., Yu, Y., Ma, C., Jiao, F., Yao, H., Lu, C., Zhang, W., 2015. Identification of Novel Laminin- and Fibronectin-binding Proteins by Far-Western Blot: Capturing the Adhesins of Streptococcus suis Type 2. Front Cell Infect Microbiol 5, 82. https://doi.org/10.3389/fcimb.2015.00082
Li, Q., Ma, C., Fu, Y., He, Y., Yu, Y., Du, D., Yao, H., Lu, C., Zhang, W., 2017c. Factor H specifically capture novel Factor H-binding proteins of Streptococcus suis and contribute to the virulence of the bacteria. Microbiol Res 196, 17–25. https://doi.org/10.1016/j.micres.2016.11.011
Li, Q., Zhang, Y., Dechao, D., Yanfei, Y., Zhang, W., 2018. Characterization and functional analysis of PnuC that is involved in the oxidative stress tolerance and virulence of Streptococcus suis serotype 2. Vet Microbiol 216, 198–206. https://doi.org/10.1016/j.vetmic.2018.02.013
Li, W., Wan, Y., Tao, Z., Chen, H., Zhou, R., 2013. A novel fibronectin-binding protein of Streptococcus suis serotype 2 contributes to epithelial cell invasion and in vivo dissemination. Vet Microbiol 162, 186–194. https://doi.org/10.1016/j.vetmic.2012.09.004
Li, X., Liu, P., Gan, S., Zhang, C., Zheng, Y., Jiang, Y., Yuan, Y., 2016. Mechanisms of Host-Pathogen Protein Complex Formation and Bacterial Immune Evasion of Streptococcus suis Protein Fhb. J Biol Chem 291, 17122–17132. https://doi.org/10.1074/jbc.M116.719443
Li, Y., Zheng, J.X., Zhang, H., Fan, H.J., Lu, C.P., 2014. Effect of Tran on virulence through regulating metabolism and stress tolerance of Streptococcus suis serotype 2. Microbiol Res 169, 666–674. https://doi.org/10.1016/j.micres.2014.03.002
Lin, L., Xu, L., Lv, W., Han, L., Xiang, Y., Fu, L., Jin, M., Zhou, R., Chen, H., Zhang, A., 2019. An NLRP3 inflammasome-triggered cytokine storm contributes to streptococcal toxic shock-like syndrome (STSLS). PLoS Pathog. 15. https://doi.org/10.1371/journal.ppat.1007795
Liu, F., Li, J., Yan, K., Li, H., Sun, C., Zhang, S., Yuan, F., Wang, X., Tan, C., Chen, H., Bei, W., 2018. Binding of Fibronectin to SsPepO Facilitates the Development of Streptococcus suis Meningitis. J Infect Dis 217, 973–982. https://doi.org/10.1093/infdis/jix523
Liu, H., Fu, H., Jiang, X., Liao, X., Yue, M., Li, X., Fang, W., 2019. PrsA contributes to Streptococcus suis serotype 2 pathogenicity by modulating secretion of selected virulence factors. Vet. Microbiol. 236. https://doi.org/10.1016/j.vetmic.2019.07.027
Liu, H., Lei, S., Jia, L., Xia, X., Sun, Y., Jiang, H., Zhu, R., Li, S., Qu, G., Gu, J., Langford, P.R., Lei, L., 2021a. Streptococcus suis serotype 2 enolase interaction with host brain microvascular endothelial cells and RPSA-induced apoptosis lead to loss of BBB integrity. Vet. Res. 52. https://doi.org/10.1186/s13567-020-00887-6
Liu, H., Ye, C., Fu, H., Yue, M., Li, X., Fang, W., 2021b. Stk and Stp1 participate in Streptococcus suis serotype 2 pathogenesis by regulating capsule thickness and translocation of certain virulence factors. Microb. Pathog. 152. https://doi.org/10.1016/j.micpath.2020.104607
Liu, L., Zhang, Q., Xu, Z., Huang, J., Zhu, W., Zhang, A., Sun, X., Jin, M., 2019. HP1717 contributes to streptococcus suis virulence by inducing an excessive inflammatory response and influencing the biosynthesis of the capsule. Microorganisms 7. https://doi.org/10.3390/microorganisms7110522
Liu, P., Pian, Y., Li, X., Liu, R., Xie, W., Zhang, C., Zheng, Y., Jiang, Y., Yuan, Y., 2014. Streptococcus suis adenosine synthase functions as an effector in evasion of PMN-mediated innate immunit. J Infect Dis 210, 35–45. https://doi.org/10.1093/infdis/jiu050
Lun, S., Perez-Casal, J., Connor, W., Willson, P.J., 2003. Role of suilysin in pathogenesis of Streptococcus suis capsular serotype 2. Microb Pathog 34, 27–37.
Lun, S., Willson, P.J., 2005. Putative mannose-specific phosphotransferase system component IID represses expression of suilysin in serotype 2 Streptococcus suis. Vet Microbiol 105, 169–180. https://doi.org/10.1016/j.vetmic.2004.10.017
Ma, J., Zhang, Z., Pan, Z., Bai, Q., Zhong, X., Zhu, Y., Zhang, Y., Wu, Z., Liu, G., Yao, H., 2020. Streptococcus suis uptakes carbohydrate source from host glycoproteins by n-glycans degradation system for optimal survival and full virulence during infection. Pathogens 9. https://doi.org/10.3390/pathogens9050387
Madar Johansson, M., Bélurier, E., Papageorgiou, A.C., Sundin, A.P., Rahkila, J., Kallonen, T., Nilsson, U.J., Maatsola, S., Nyholm, T.K.M., Käpylä, J., Corander, J., Leino, R., Finne, J., Teneberg, S., Haataja, S., 2020. The binding mechanism of the virulence factor Streptococcus suis adhesin P subtype to globotetraosylceramide is associated with systemic disease. J. Biol. Chem. 295, 14305–14324. https://doi.org/10.1074/jbc.RA120.014818
Meijerink, M., Ferrando, M.L., Lammers, G., Taverne, N., Smith, H.E., Wells, J.M., 2012. Immunomodulatory effects of Streptococcus suis capsule type on human dendritic cell responses, phagocytosis and intracellular survival. PLoS One 7, e35849. https://doi.org/10.1371/journal.pone.0035849
Musyoki, A.M., Shi, Z., Xuan, C., Lu, G., Qi, J., Gao, F., Zheng, B., Zhang, Q., Li, Y., Haywood, J., Liu, C., Yan, J., Shi, Y., Gao, G.F., 2016. Structural and functional analysis of an anchorless fibronectin-binding protein FBPS from Gram-positive bacterium Streptococcus suis. Proc Natl Acad Sci U S A 113, 13869–13874. https://doi.org/10.1073/pnas.1608406113
Ni, H., Li, M., Wang, Q., Wang, J., Liu, X., Zheng, F., Hu, D., Yu, X., Han, Y., Zhang, Q., Shao, Z.-Q., Pan, X., 2020. Inactivation of the htpsA gene affects capsule development and pathogenicity of Streptococcus suis. Virulence 11, 927–940. https://doi.org/10.1080/21505594.2020.1792080
Norton, P.M., Rolph, C., Ward, P.N., Bentley, R.W., Leigh, J.A., 1999. Epithelial invasion and cell lysis by virulent strains of Streptococcus suis is enhanced by the presence of suilysin. FEMS Immunol Med Microbiol 26, 25–35. https://doi.org/10.1111/j.1574-695X.1999.tb01369.x
Pan, X., Ge, J., Li, M., Wu, B., Wang, C., Wang, J., Feng, Y., Yin, Z., Zheng, F., Cheng, G., Sun, W., Ji, H., Hu, D., Shi, P., Feng, X., Hao, X., Dong, R., Hu, F., Tang, J., 2009. The orphan response regulator CovR: a globally negative modulator of virulence in Streptococcus suis serotype 2. J Bacteriol 191, 2601–2612. https://doi.org/10.1128/JB.01309-08
Pian, Y., Gan, S., Wang, S., Guo, J., Wang, P., Zheng, Y., Cai, X., Jiang, Y., Yuan, Y., 2012. Fhb, a novel factor H-binding surface protein, contributes to the antiphagocytic ability and virulence of Streptococcus suis. Infect Immun 80, 2402–2413. https://doi.org/10.1128/IAI.06294-11
Pian, Y., Li, X., Zheng, Y., Wu, X., Yuan, Y., Jiang, Y., 2016. Binding of Human Fibrinogen to MRP Enhances Streptococcus suis Survival in Host Blood in a alphaXbeta2 Integrin-dependent Manner. Sci Rep 6, 26966. https://doi.org/10.1038/srep26966
Pian, Y., Wang, P., Liu, P., Zheng, Y., Zhu, L., Wang, H., Xu, B., Yuan, Y., Jiang, Y., 2015. Proteomics identification of novel fibrinogen-binding proteins of Streptococcus suis contributing to antiphagocytosis. Front Cell Infect Microbiol 5, 19. https://doi.org/10.3389/fcimb.2015.00019
Qian, Y., Zhang, Y., Yu, Y., Li, Q., Guo, G., Fu, Y., Yao, H., Lu, C., Zhang, W., 2018. SBP1 is an adhesion-associated factor without the involvement of virulence in Streptococcus suis serotype 2. Microb Pathog 122, 90–97. https://doi.org/10.1016/j.micpath.2018.06.008
Roy, D., Grenier, D., Segura, M., Mathieu-Denoncourt, A., Gottschalk, M., 2016. Recruitment of Factor H to the Streptococcus suis Cell Surface is Multifactorial. Pathogens 5. https://doi.org/10.3390/pathogens5030047

Roy, S., Zhu, Y., Ma, J., Roy, A.C., Zhang, Y., Zhong, X., Pan, Z., Yao, H., 2019. Role of ClpX and ClpP in Streptococcus suis serotype 2 stress tolerance and virulence. Microbiol. Res. 223–225, 99–109. https://doi.org/10.1016/j.micres.2019.04.003
Rui, L., Weiyi, L., Yu, M., Hong, Z., Jiao, Y., Zhe, M., Hongjie, F., 2018. The serine/threonine protein kinase of Streptococcus suis serotype 2 affects the ability of the pathogen to penetrate the blood-brain barrier. Cell Microbiol 20, e12862. https://doi.org/10.1111/cmi.12862
Salasia, S.I., Lammler, C., Herrmann, G., 1995. Properties of a Streptococcus suis isolate of serotype 2 and two capsular mutants. Vet Microbiol 45, 151–156.
Schwerk, C., Papandreou, T., Schuhmann, D., Nickol, L., Borkowski, J., Steinmann, U., Quednau, N., Stump, C., Weiss, C., Berger, J., Wolburg, H., Claus, H., Vogel, U., Ishikawa, H., Tenenbaum, T., Schroten, H., 2012. Polar invasion and translocation of Neisseria meningitidis and Streptococcus suis in a novel human model of the blood-cerebrospinal fluid barrier. PLoS One 7, e30069. https://doi.org/10.1371/journal.pone.0030069
Seele, J., Singpiel, A., Spoerry, C., von Pawel-Rammingen, U., Valentin-Weigand, P., Baums, C.G., 2013. Identification of a novel host-specific IgM protease in Streptococcus suis. J Bacteriol 195, 930–940. https://doi.org/10.1128/JB.01875-12
Seitz, M., Baums, C.G., Neis, C., Benga, L., Fulde, M., Rohde, M., Goethe, R., Valentin-Weigand, P., 2013. Subcytolytic effects of suilysin on interaction of Streptococcus suis with epithelial cells. Vet Microbiol 167, 584–591. https://doi.org/10.1016/j.vetmic.2013.09.010
Shao, J., Zhang, W., Wu, Z., Lu, C., 2014. The truncated major pilin subunit Sbp2 of the srtBCD pilus cluster still contributes to Streptococcus suis pathogenesis in the absence of pilus shaft. Curr Microbiol 69, 703–707. https://doi.org/10.1007/s00284-014-0642-4
Si, Y., Yuan, F., Chang, H., Liu, X., Li, H., Cai, K., Xu, Z., Huang, Q., Bei, W., Chen, H., 2009. Contribution of glutamine synthetase to the virulence of Streptococcus suis serotype 2. Vet Microbiol 139, 80–88. https://doi.org/10.1016/j.vetmic.2009.04.024
Spoerry, C., Seele, J., Valentin-Weigand, P., Baums, C.G., von Pawel-Rammingen, U., 2016. Identification and Characterization of IgdE, a Novel IgG-degrading Protease of Streptococcus suis with Unique Specificity for Porcine IgG. J Biol Chem 291, 7915–7925. https://doi.org/10.1074/jbc.M115.711440
Tan, C., Liu, M., Li, J., Jin, M., Bei, W., Chen, H., 2011. SsPep contributes to the virulence of Streptococcus suis. Microb. Pathog. 51, 319–324. https://doi.org/10.1016/j.micpath.2011.07.008
Tan, M.F., Liu, W.Q., Zhang, C.Y., Gao, T., Zheng, L.L., Qiu, D.X., Li, L., Zhou, R., 2017. The involvement of MsmK in pathogenesis of the Streptococcus suis serotype 2. Microbiologyopen 6. https://doi.org/10.1002/mbo3.433
Vadeboncoeur, N., Segura, M., Al-Numani, D., Vanier, G., Gottschalk, M., 2003. Pro-inflammatory cytokine and chemokine release by human brain microvascular endothelial cells stimulated by Streptococcus suis serotype 2. FEMS Immunol Med Microbiol 35, 49–58. https://doi.org/10.1111/j.1574-695X.2003.tb00648.x
Wang, C., Li, M., Feng, Y., Zheng, F., Dong, Y., Pan, X., Cheng, G., Dong, R., Hu, D., Feng, X., Ge, J., Liu, D., Wang, J., Cao, M., Hu, F., Tang, J., 2009. The involvement of sortase A in high virulence of STSS-causing Streptococcus suis serotype 2. Arch Microbiol 191, 23–33. https://doi.org/10.1007/s00203-008-0425-z
Wang, J., Feng, Y., Wang, C., Srinivas, S., Chen, C., Liao, H., He, E., Jiang, S., Tang, J., 2017. Pathogenic Streptococcus strains employ novel escape strategy to inhibit bacteriostatic effect mediated by mammalian peptidoglycan recognition protein. Cell Microbiol 19. https://doi.org/10.1111/cmi.12724
Wang, J., Kong, D., Zhang, S., Jiang, H., Zheng, Y., Zang, Y., Hao, H., Jiang, Y., 2015. Interaction of fibrinogen and muramidase-released protein promotes the development of Streptococcus suis meningitis. Front Microbiol 6, 1001. https://doi.org/10.3389/fmicb.2015.01001
Wang, Y., Yi, L., Zhang, Z., Fan, H., Cheng, X., Lu, C., 2014. Biofilm formation, host-cell adherence, and virulence genes regulation of Streptococcus suis in response to autoinducer-2 signaling. Curr Microbiol 68, 575–580. https://doi.org/10.1007/s00284-013-0509-0
Wang, Y., Zhang, W., Wu, Z., Zhu, X., Lu, C., 2011. Functional analysis of luxS in Streptococcus suis reveals a key role in biofilm formation and virulence. Vet Microbiol 152, 151–160. https://doi.org/10.1016/j.vetmic.2011.04.029
Willenborg, J., Koczula, A., Fulde, M., de Greeff, A., Beineke, A., Eisenreich, W., Huber, C., Seitz, M., Valentin-Weigand, P., Goethe, R., 2016. FlpS, the FNR-Like Protein of Streptococcus suis Is an Essential, Oxygen-Sensing Activator of the Arginine Deiminase System. Pathogens 5. https://doi.org/10.3390/pathogens5030051
Wu, T., Chang, H., Tan, C., Bei, W., Chen, H., 2009. The orphan response regulator RevSC21 controls the attachment of Streptococcus suis serotype-2 to human laryngeal epithelial cells and the expression of virulence genes. FEMS Microbiol Lett 292, 170–181. https://doi.org/10.1111/j.1574-6968.2008.01486.x
Wu, Z., Wu, C., Shao, J., Zhu, Z., Wang, W., Zhang, W., Tang, M., Pei, N., Fan, H., Li, J., Yao, H., Gu, H., Xu, X., Lu, C., 2014. The Streptococcus suis transcriptional landscape reveals adaptation mechanisms in pig blood and cerebrospinal fluid. RNA 20, 882–898. https://doi.org/10.1261/rna.041822.113
Xiao, G., Wu, Z., Zhang, S., Tang, H., Wang, F., Lu, C., 2017. Mac Protein is not an Essential Virulence Factor for the Virulent Reference Strain Streptococcus suis P1/7. Curr Microbiol 74, 90–96. https://doi.org/10.1007/s00284-016-1160-3
Xie, F., Zan, Y., Zhang, Y., Zheng, N., Yan, Q., Zhang, W., Zhang, H., Jin, M., Chen, F., Zhang, X., Zhang, X., Liu, S., 2019. The cysteine protease ApdS from Streptococcus suis promotes evasion of innate immune defenses by cleaving the antimicrobial peptide cathelicidin LL-37. J. Biol. Chem. 294, 17962–17977. https://doi.org/10.1074/jbc.RA119.009441
Xu, J., Fu, S., Liu, M., Xu, Q., Bei, W., Chen, H., Tan, C., 2014. The two-component system NisK/NisR contributes to the virulence of Streptococcus suis serotype 2. Microbiol Res 169, 541–546. https://doi.org/10.1016/j.micres.2013.11.002
Yi, L., Li, J., Fan, Q., Mao, C., Jin, M., Liu, Y., Sun, L., Grenier, D., Wang, Y., 2020. The otc gene of Streptococcus suis plays an important role in biofilm formation, adhesion, and virulence in a murine model. Vet. Microbiol. 251. https://doi.org/10.1016/j.vetmic.2020.108925
Yu, J., Pian, Y., Ge, J., Guo, J., Zheng, Y., Jiang, H., Hao, H., Yuan, Y., Jiang, Y., Yang, M., 2015. Functional and Structural Characterization of the Antiphagocytic Properties of a Novel Transglutaminase from Streptococcus suis. J Biol Chem 290, 19081–19092. https://doi.org/10.1074/jbc.M115.643338
Yu, Y., Qian, Y., Du, D., Li, Q., Xu, C., Liu, H., Chen, M., Yao, H., Lu, C., Zhang, W., 2018. Infection and adaption-based proteomic changes of Streptococcus suis serotype 2 in a pig model. J Proteomics 180, 41–52. https://doi.org/10.1016/j.jprot.2017.12.001
Yu, Y., Qian, Y., Du, D., Xu, C., Dai, C., Li, Q., Liu, H., Shao, J., Wu, Z., Zhang, W., 2016. SBP2 plays an important role in the virulence changes of different artificial mutants of Streptococcus suis. Mol Biosyst 12, 1948–1962. https://doi.org/10.1039/c6mb00059b
Yuan, F., Tan, C., Liu, Z., Yang, K., Zhou, D., Liu, W., Duan, Z., Guo, R., Chen, H., Tian, Y., Bei, W., 2017. The 1910HK/RR two-component system is essential for the virulence of Streptococcus suis serotype 2. Microb Pathog 104, 137–145. https://doi.org/10.1016/j.micpath.2016.12.026
Yuan, Z.Z., Yan, X.J., Zhang, A.D., Chen, B., Shen, Y.Q., Jin, M.L., 2013. Molecular mechanism by which surface antigen HP0197 mediates host cell attachment in the pathogenic bacteria Streptococcus suis. J Biol Chem 288, 956–963. https://doi.org/10.1074/jbc.M112.388686
Zaccaria, E., Cao, R., Wells, J.M., van Baarlen, P., 2016. A Zebrafish Larval Model to Assess Virulence of Porcine Streptococcus suis Strains. PLoS One 11, e0151623. https://doi.org/10.1371/journal.pone.0151623
Zhang, H., Fan, H., Lu, C., 2010. Identification of a novel virulence-related gene in Streptococcus suis type 2 strains. Curr Microbiol 61, 494–499. https://doi.org/10.1007/s00284-010-9643-0
Zhang, H., Ma, Z., Li, Y., Zheng, J., Yi, L., Fan, H., Lu, C., 2013. Identification of a novel collagen type I{cyrillic, ukrainian}-binding protein from Streptococcus suis serotype 2. Vet. J. 197, 406–414. https://doi.org/10.1016/j.tvjl.2013.01.030
Zhang, H., Ravcheev, D.A., Hu, D., Zhang, F., Gong, X., Hao, L., Cao, M., Rodionov, D.A., Wang, C., Feng, Y., 2015. Two novel regulators of N-acetyl-galactosamine utilization pathway and distinct roles in bacterial infections. Microbiologyopen 4, 983–1000. https://doi.org/10.1002/mbo3.307
Zhang, H., Zheng, J., Yi, L., Li, Y., Ma, Z., Fan, H., Lu, C., 2014. The identification of six novel proteins with fibronectin or collagen type I binding activity from Streptococcus suis serotype 2. J Microbiol 52, 963–969. https://doi.org/10.1007/s12275-014-4311-x
Zhang, S., Zheng, Y., Chen, S., Huang, S., Liu, K., Lv, Q., Jiang, Y., Yuan, Y., 2016. Suilysin-induced Platelet-Neutrophil Complexes Formation is Triggered by Pore Formation-dependent Calcium Influx. Sci Rep 6, 36787. https://doi.org/10.1038/srep36787
Zhang, W., Liu, G., Tang, F., Shao, J., Lu, Y., Bao, Y., Yao, H., Lu, C., 2011. Pre-absorbed immunoproteomics: a novel method for the detection of Streptococcus suis surface proteins. PLoS One 6, e21234. https://doi.org/10.1371/journal.pone.0021234
Zhang, X., Jiang, X., Yang, L., Fang, L., Shen, H., Lu, X., Fang, W., 2014. Dnaj of streptococcus suis type 2 contributes to cell adhesion and thermotolerance. J. Microbiol. Biotechnol. 25, 771–781. https://doi.org/10.4014/jmb.1408.08085
Zhang, Y., Ding, D., Liu, M., Yang, X., Zong, B., Wang, X., Chen, H., Bei, W., Tan, C., 2016. Effect of the glycosyltransferases on the capsular polysaccharide synthesis of Streptococcus suis serotype 2. Microbiol Res 185, 45–54. https://doi.org/10.1016/j.micres.2016.02.002
Zhang, Y., Lu, P., Pan, Z., Zhu, Y., Ma, J., Zhong, X., Dong, W., Lu, C., Yao, H., 2018. SssP1, a Streptococcus suis Fimbria-Like Protein Transported by the SecY2/A2 System, Contributes to Bacterial Virulence. Appl Env. Microbiol 84. https://doi.org/10.1128/AEM.01385-18
Zhang, Y., Zhong, X., Lu, P., Zhu, Y., Dong, W., Roy, S., Hejair, H.M.A., Pan, Z., Ma, J., Yao, H., 2019. A novel autolysin AtlA<inf>SS</inf> mediates bacterial cell separation during cell division and contributes to full virulence in Streptococcus suis. Vet. Microbiol. 234, 92–100. https://doi.org/10.1016/j.vetmic.2019.05.020
Zhao, Z., Shang, X., Chen, Y., Zheng, Y., Huang, W., Jiang, H., Lv, Q., Kong, D., Jiang, Y., Liu, P., 2020. Bacteria elevate extracellular adenosine to exploit host signaling for blood-brain barrier disruption. Virulence 11, 980–994. https://doi.org/10.1080/21505594.2020.1797352
Zheng, F., Ji, H., Cao, M., Wang, C., Feng, Y., Li, M., Pan, X., Wang, J., Qin, Y., Hu, F., Tang, J., 2011. Contribution of the Rgg transcription regulator to metabolism and virulence of Streptococcus suis serotype 2. Infect Immun 79, 1319–1328. https://doi.org/10.1128/IAI.00193-10
Zheng, F., Shao, Z.Q., Hao, X., Wu, Q., Li, C., Hou, H., Hu, D., Wang, C., Pan, X., 2018. Identification of oligopeptide-binding protein (OppA) and its role in the virulence of Streptococcus suis serotype 2. Microb Pathog 118, 322–329. https://doi.org/10.1016/j.micpath.2018.03.061
Zheng, J.X., Li, Y., Zhang, H., Fan, H.J., Lu, C.P., 2013. Identification and characterization of a novel hemolysis-related gene in Streptococcus suis serotype 2. PLoS One 8, e74674. https://doi.org/10.1371/journal.pone.0074674
Zhou, J., Zhang, X., He, K., Wang, W., Ni, Y., Zhu, H., Yu, Z., Mao, A., Lv, L., 2014. Characterization and proteome analysis of inosine 5-monophosphate dehydrogenase in epidemic Streptococcus suis serotype 2. Curr Microbiol 68, 663–669. https://doi.org/10.1007/s00284-014-0527-6
Zhou, Y., Nie, R., Liu, X., Kong, J., Wang, X., Li, J., 2018. GntR is involved in the expression of virulence in strain Streptococcus suis P1/7. FEMS Microbiol Lett 365. https://doi.org/10.1093/femsle/fny091
Zhu, H., Huang, D., Zhang, W., Wu, Z., Lu, Y., Jia, H., Wang, M., Lu, C., 2011. The novel virulence-related gene stp of Streptococcus suis serotype 9 strain contributes to a significant reduction in mouse mortality. Microb Pathog 51, 442–453. https://doi.org/10.1016/j.micpath.2011.08.002
Zhu, H., Wang, Y., Ni, Y., Zhou, J., Han, L., Yu, Z., Mao, A., Wang, D., Fan, H., He, K., 2018. The Redox-Sensing Regulator Rex Contributes to the Virulence and Oxidative Stress Response of Streptococcus suis Serotype 2. Front Cell Infect Microbiol 8, 317. https://doi.org/10.3389/fcimb.2018.00317
Zhu, H., Zhou, J., Ni, Y., Yu, Z., Mao, A., Hu, Y., Wang, W., Zhang, X., Wen, L., Li, B., Wang, X., Yu, Y., Lv, L., Guo, R., Lu, C., He, K., 2014. Contribution of eukaryotic-type serine/threonine kinase to stress response and virulence of Streptococcus suis. PLoS One 9, e91971. https://doi.org/10.1371/journal.pone.0091971
Zhu, J., Zhang, T., Su, Z., Feng, L., Liu, H., Xu, Z., Wu, Y., Gao, T., Shao, H., Zhou, R., 2019. Co-regulation of CodY and (p)ppGpp synthetases on morphology and pathogenesis of Streptococcus suis. Microbiol. Res. 223–225, 88–98. https://doi.org/10.1016/j.micres.2019.04.001
Zhu, J., Zhang, T., Su, Z., Li, L., Wang, D., Xiao, R., Teng, M., Tan, M., Zhou, R., 2016. (p)ppGpp synthetases regulate the pathogenesis of zoonotic Streptococcus suis. Microbiol Res 191, 1–11. https://doi.org/10.1016/j.micres.2016.05.007
