# Supplementary material for: Identification of Streptococcus suis putative zoonotic virulence factors: A systematic review and genomic meta-analysis
Source: Virulence. 2021 Nov 25;12(1):2787–97. doi: 10.1080/21505594.2021.1985760 (PMC8632099; doi:10.1080/21505594.2021.1985760)
Supplement: Supplemental Material [file KVIR_A_1985760_SM7428.zip › supplementary/2021.09.22_Appendix3_includedBiosamples.pdf]

Name	Reference	Bioproject	Host	Serotype	Sequence Type	Clonal Complex	Country of Isolation
SAMD00009254	1	PRJDB1485	PH	2	28	2	Japan
SAMD00009255	1	PRJDB1485	H	2	28	2	Thailand
SAMD00009256	1	PRJDB1485	PD	6	55	25	Denmark
SAMD00009257	1	PRJDB1485	PD	5	53	8	Denmark
SAMD00009259	1	PRJDB1485	PD	1	13	9	Netherlands
SAMD00009260	1	PRJDB1485	PH	19	76	34	Canada
SAMD00009262	1	PRJDB1485	PD	27	72	31	Canada
SAMD00009263	1	PRJDB1485	PD	3	35	2	Denmark
SAMD00009264	1	PRJDB1485	PD	9	82	39	Denmark
SAMD00009265	1	PRJDB1485	PD	2	1	1	Japan
SAMD00009266	1	PRJDB1485	PD	23	483	0	Canada
SAMD00009267	1	PRJDB1485	PD	Unknown	108	0	Japan
SAMD00009268	1	PRJDB1485	PD	24	68	28	Canada
SAMD00009270	1	PRJDB1485	PD	2	28	2	Japan
SAMD00009271	1	PRJDB1485	PD	4	54	8	Denmark
SAMD00009272	1	PRJDB1485	PD	8	87	3	Denmark
SAMD00009273	1	PRJDB1485	PH	17	76	34	Canada
SAMD00009275	1	PRJDB1485	PH	2	28	2	Japan
SAMD00009276	1	PRJDB1485	PD	29	92	41	Canada
SAMD00009277	1	PRJDB1485	PD	16	73	32	Denmark
SAMD00009278	1	PRJDB1485	PH	21	481	81	Canada
SAMD00009280	1	PRJDB1485	PD	10	78	36	Denmark
SAMD00009281	1	PRJDB1485	PH	18	79	37	Canada
SAMD00009282	1	PRJDB1485	H	2	101	5	Thailand
SAMD00009284	1	PRJDB1485	PD	2	56	27	Netherlands
SAMD00009285	1	PRJDB1485	PD	15	81	38	Netherlands
SAMD00009286	1	PRJDB1485	H	2	102	2	Thailand
SAMD00009287	1	PRJDB1485	H	1	6	1	Netherlands
SAMD00009288	1	PRJDB1485	PD	13	71	30	Denmark
SAMD00009289	1	PRJDB1485	H	2	104	5	Thailand
SAMD00009291	1	PRJDB1485	H	2	25	2	Thailand
SAMD00009292	1	PRJDB1485	H	1	11	1	Thailand
SAMD00009293	1	PRJDB1485	PH	4	94	0	Japan
SAMD00009294	1	PRJDB1485	PD	11	91	40	Denmark
SAMD00009295	1	PRJDB1485	PD	12	775	95	Denmark
SAMD00009296	1	PRJDB1485	PD	25	69	29	Canada
SAMD00009297	1	PRJDB1485	H	2	1	1	Japan
SAMD00009298	1	PRJDB1485	PD	7	29	2	Denmark
SAMD00009299	1	PRJDB1485	PD	28	75	33	Canada
SAMEA104027775	2	PRJEB20548	PD	9	16	4	Netherlands
SAMEA104027776	2	PRJEB20548	PD	9	16	4	Netherlands
SAMEA104027777	2	PRJEB20548	PD	9	16	4	Netherlands
SAMEA104027778	2	PRJEB20548	PD	9	16	4	Netherlands
SAMEA104027779	2	PRJEB20548	PD	9	819	6	Netherlands
SAMEA104027780	2	PRJEB20548	PD	9	819	6	Netherlands
SAMEA104027781	2	PRJEB20548	PD	9	16	4	Netherlands
SAMEA104027782	2	PRJEB20548	PD	9	16	4	Netherlands
SAMEA104027783	2	PRJEB20548	PD	9	16	4	Netherlands
SAMEA104027784	2	PRJEB20548	PD	9	804	4	Netherlands
SAMEA104027785	2	PRJEB20548	PD	9	16	4	Netherlands
SAMEA104027786	2	PRJEB20548	PD	9	16	4	Netherlands
SAMEA104027787	2	PRJEB20548	PH	9	820	108	France
SAMEA104027788	2	PRJEB20548	PD	9	16	4	Netherlands
SAMEA104027789	2	PRJEB20548	PD	9	16	4	Netherlands
SAMEA104027790	2	PRJEB20548	PD	9	16	4	Netherlands
SAMEA104027791	2	PRJEB20548	PD	9	16	4	Netherlands
SAMEA104027792	2	PRJEB20548	PH	9	802	100	Netherlands
SAMEA104027793	2	PRJEB20548	PH	9	802	100	Netherlands
SAMEA104027794	2	PRJEB20548	PH	9	808	102	Netherlands
SAMEA104027795	2	PRJEB20548	PH	9	808	102	Netherlands
SAMEA104027796	2	PRJEB20548	PH	9	802	100	Netherlands
SAMEA104027797	2	PRJEB20548	PH	9	808	102	Netherlands
SAMEA104027798	2	PRJEB20548	PH	9	808	102	Netherlands
SAMEA104027799	2	PRJEB20548	PH	9	16	4	Netherlands
SAMEA104027800	2	PRJEB20548	PH	9	808	102	Netherlands
SAMEA104027801	2	PRJEB20548	PH	9	16	4	Netherlands
SAMEA104027802	2	PRJEB20548	PH	9	16	4	Netherlands
SAMEA104027803	2	PRJEB20548	PH	9	16	4	Netherlands
SAMEA104027804	2	PRJEB20548	PH	9	807	101	Netherlands
SAMEA104027805	2	PRJEB20548	PH	9	807	101	Netherlands
SAMEA104027806	2	PRJEB20548	PH	9	807	101	Netherlands
SAMEA104027807	2	PRJEB20548	PH	9	807	101	Netherlands
SAMEA104027808	2	PRJEB20548	PH	9	807	101	Netherlands
SAMEA104027809	2	PRJEB20548	PH	9	807	101	Netherlands
SAMEA104027810	2	PRJEB20548	PH	9	807	101	Netherlands
SAMEA104027811	2	PRJEB20548	PH	9	817	106	Netherlands
SAMEA104027812	2	PRJEB20548	PH	9	817	106	Netherlands
SAMEA104027813	2	PRJEB20548	PH	9	811	105	Netherlands
SAMEA104027814	2	PRJEB20548	PH	9	811	105	Netherlands
SAMEA104027815	2	PRJEB20548	PH	9	811	105	Netherlands
SAMEA104027816	2	PRJEB20548	PH	9	810	104	Netherlands
SAMEA104027817	2	PRJEB20548	PH	9	810	104	Netherlands
SAMEA104027818	2	PRJEB20548	PH	9	810	104	Netherlands
SAMEA104027819	2	PRJEB20548	PH	9	810	104	Netherlands
SAMEA104027820	2	PRJEB20548	PH	9	810	104	Netherlands
SAMEA104027821	2	PRJEB20548	PH	9	810	104	Netherlands
SAMEA104027822	2	PRJEB20548	PH	9	810	104	Netherlands
SAMEA104027823	2	PRJEB20548	PH	9	810	104	Netherlands
SAMEA104027824	2	PRJEB20548	PH	9	810	104	Netherlands
SAMEA104027825	2	PRJEB20548	PH	9	809	103	Netherlands
SAMEA104027826	2	PRJEB20548	PH	9	818	107	Netherlands
SAMEA104027827	2	PRJEB20548	PH	9	809	103	Netherlands
SAMEA104027828	2	PRJEB20548	PH	9	809	103	Netherlands
SAMEA104027829	2	PRJEB20548	PH	9	818	107	Netherlands
SAMEA104027830	2	PRJEB20548	PH	9	809	103	Netherlands
SAMEA104027831	2	PRJEB20548	PH	9	Unknown	Unknown	Netherlands
SAMEA104027832	2	PRJEB20548	PH	9	809	103	Netherlands
SAMEA104027833	2	PRJEB20548	PH	9	818	107	Netherlands
SAMEA104027834	2	PRJEB20548	PH	9	48	26	Netherlands
SAMEA104027835	2	PRJEB20548	PH	9	48	26	Netherlands
SAMEA104027836	2	PRJEB20548	PH	9	48	26	Netherlands
SAMEA104027837	2	PRJEB20548	PD	9	16	4	Netherlands
SAMEA104027838	2	PRJEB20548	PD	9	136	4	Netherlands
SAMEA104027839	2	PRJEB20548	PD	9	16	4	Netherlands
SAMEA104027840	2	PRJEB20548	PD	9	136	4	Netherlands
SAMEA104027841	2	PRJEB20548	PD	9	16	4	Netherlands
SAMEA104027842	2	PRJEB20548	PD	9	136	4	Netherlands
SAMEA104027843	2	PRJEB20548	PD	9	147	6	Netherlands
SAMEA104027844	2	PRJEB20548	PD	9	16	4	Netherlands
SAMEA104027845	2	PRJEB20548	PD	9	136	4	Netherlands
SAMEA104027846	2	PRJEB20548	PD	9	16	4	Netherlands
SAMEA104027847	2	PRJEB20548	PD	9	136	4	Netherlands
SAMEA104027848	2	PRJEB20548	PD	9	16	4	Netherlands
SAMEA104027849	2	PRJEB20548	PD	9	16	4	Netherlands
SAMEA104027850	2	PRJEB20548	PD	9	16	4	Netherlands
SAMEA104027851	2	PRJEB20548	PD	9	16	4	Netherlands
SAMEA104027852	2	PRJEB20548	PD	9	16	4	Netherlands

SAMEA104027853	2	PRJEB20548	PD	9	16	4	Netherlands
SAMEA104027854	2	PRJEB20548	PD	9	803	4	Netherlands
SAMEA104027855	2	PRJEB20548	PD	9	16	4	Netherlands
SAMEA104027856	2	PRJEB20548	PD	9	16	4	Netherlands
SAMEA104027857	2	PRJEB20548	PD	9	16	4	Netherlands
SAMEA104027858	2	PRJEB20548	PD	9	16	4	Netherlands
SAMEA104027859	2	PRJEB20548	PD	9	16	4	Netherlands
SAMEA104027860	2	PRJEB20548	PD	9	136	4	Netherlands
SAMEA104027861	2	PRJEB20548	PD	9	16	4	Netherlands
SAMEA104027862	2	PRJEB20548	PD	9	803	4	Netherlands
SAMEA104027863	2	PRJEB20548	PD	9	136	4	Netherlands
SAMEA104027864	2	PRJEB20548	PD	9	16	4	Netherlands
SAMEA104027865	2	PRJEB20548	PD	9	16	4	Netherlands
SAMEA104027866	2	PRJEB20548	PD	9	16	4	Netherlands
SAMEA104027867	2	PRJEB20548	PD	9	16	4	Netherlands
SAMEA104027868	2	PRJEB20548	PD	9	16	4	Netherlands
SAMEA104027869	2	PRJEB20548	PD	9	805	4	Netherlands
SAMEA104027870	2	PRJEB20548	PD	9	811	105	Netherlands
SAMN07176407	3	PRJNA197478	PH	Unknown	444	76	China
SAMN07176409	3	PRJNA197476	PH	Unknown	453	78	China
SAMN07176411	3	PRJNA197474	PH	Unknown	408	71	China
SAMN07176412	3	PRJNA197473	PH	Unknown	404	70	China
SAMN07176414	3	PRJNA197471	PH	Unknown	453	78	China
SAMN07176416	3	PRJNA197469	PH	Unknown	436	75	China
SAMN07176418	3	PRJNA197467	PH	Unknown	434	74	China
SAMN07176420	3	PRJNA197464	PH	Unknown	431	13	China
SAMN07176421	3	PRJNA197463	PH	Unknown	399	13	China
SAMEA3233752	4	PRJEB8392	PD	2	1	1	Vietnam
SAMEA3233753	4	PRJEB8392	PD	2	1	1	Vietnam
SAMEA3233754	4	PRJEB8392	PD	2	1	1	Vietnam
SAMEA3233755	4	PRJEB8392	PD	2	1	1	Vietnam
SAMEA3233756	4	PRJEB8392	H	2	1	1	Vietnam
SAMEA3233757	4	PRJEB8392	H	2	1	1	Vietnam
SAMEA3233758	4	PRJEB8392	H	2	1	1	Vietnam
SAMEA3233759	4	PRJEB8392	H	2	1	1	Vietnam
SAMEA3233760	4	PRJEB8392	H	2	1	1	Vietnam
SAMEA3233761	4	PRJEB8392	H	2	1	1	Vietnam
SAMEA3233765	4	PRJEB8392	H	2	1	1	Vietnam
SAMEA3233766	4	PRJEB8392	H	2	1	1	Vietnam
SAMEA3233767	4	PRJEB8392	H	2	1	1	Vietnam
SAMEA3233768	4	PRJEB8392	H	2	1	1	Vietnam
SAMEA3233770	4	PRJEB8392	H	2	1	1	Vietnam
SAMEA3233771	4	PRJEB8392	H	2	1	1	Vietnam
SAMEA3233772	4	PRJEB8392	H	2	1	1	Vietnam
SAMEA3233773	4	PRJEB8392	H	2	1	1	Vietnam
SAMEA3233774	4	PRJEB8392	H	2	1	1	Vietnam
SAMEA3233775	4	PRJEB8392	H	2	1	1	Vietnam
SAMEA3233776	4	PRJEB8392	H	2	1	1	Vietnam
SAMEA3233777	4	PRJEB8392	H	2	1	1	Vietnam
SAMEA3233778	4	PRJEB8392	H	2	1	1	Vietnam
SAMEA3233779	4	PRJEB8392	H	2	1	1	Vietnam
SAMEA3233780	4	PRJEB8392	H	2	1	1	Vietnam
SAMEA3233781	4	PRJEB8392	H	2	1	1	Vietnam
SAMEA3233782	4	PRJEB8392	H	2	1	1	Vietnam
SAMEA3233783	4	PRJEB8392	H	2	1	1	Vietnam
SAMEA3233784	4	PRJEB8392	H	2	1	1	Vietnam
SAMEA3233785	4	PRJEB8392	H	2	1	1	Vietnam
SAMEA3233786	4	PRJEB8392	H	2	1	1	Vietnam
SAMEA3233787	4	PRJEB8392	H	2	1	1	Vietnam
SAMEA3233788	4	PRJEB8392	H	2	1	1	Vietnam
SAMEA3233789	4	PRJEB8392	H	2	1	1	Vietnam
SAMEA3233790	4	PRJEB8392	H	2	1	1	Vietnam
SAMEA3233791	4	PRJEB8392	H	2	1	1	Vietnam
SAMEA3233792	4	PRJEB8392	H	2	1	1	Vietnam
SAMEA3233793	4	PRJEB8392	H	2	1	1	Vietnam
SAMEA3233794	4	PRJEB8392	H	2	1	1	Vietnam
SAMEA3233795	4	PRJEB8392	H	2	1	1	Vietnam
SAMEA3233796	4	PRJEB8392	H	2	1	1	Vietnam
SAMEA3233797	4	PRJEB8392	H	2	1	1	Vietnam
SAMEA3233798	4	PRJEB8392	H	2	1	1	Vietnam
SAMEA3233799	4	PRJEB8392	H	2	1	1	Vietnam
SAMEA3233800	4	PRJEB8392	H	2	1	1	Vietnam
SAMEA3233801	4	PRJEB8392	H	2	1	1	Vietnam
SAMEA3233802	4	PRJEB8392	H	2	1	1	Vietnam
SAMEA3233803	4	PRJEB8392	PD	2	1	1	Vietnam
SAMEA3233804	4	PRJEB8392	PD	2	1	1	Vietnam
SAMEA3233805	4	PRJEB8392	H	2	1	1	Vietnam
SAMEA3233806	4	PRJEB8392	H	2	1	1	Vietnam
SAMEA3233807	4	PRJEB8392	H	2	1	1	Vietnam
SAMEA3233808	4	PRJEB8392	H	2	1	1	Vietnam
SAMEA3233809	4	PRJEB8392	H	2	1	1	Vietnam
SAMEA3233810	4	PRJEB8392	H	2	1	1	Vietnam
SAMEA3233811	4	PRJEB8392	H	2	1	1	Vietnam
SAMEA3233812	4	PRJEB8392	H	2	1	1	Vietnam
SAMEA3233813	4	PRJEB8392	H	2	1	1	Vietnam
SAMEA3233814	4	PRJEB8392	H	2	1	1	Vietnam
SAMEA3233815	4	PRJEB8392	H	2	1	1	Vietnam
SAMEA3233816	4	PRJEB8392	H	2	1	1	Vietnam
SAMEA3233817	4	PRJEB8392	H	2	1	1	Vietnam
SAMEA3233818	4	PRJEB8392	H	1	105	1	Vietnam
SAMEA3233819	4	PRJEB8392	H	2	1	1	Vietnam
SAMEA3233820	4	PRJEB8392	H	2	1	1	Vietnam
SAMEA3233821	4	PRJEB8392	H	2	1	1	Vietnam
SAMEA3233822	4	PRJEB8392	H	2	1	1	Vietnam
SAMEA3233823	4	PRJEB8392	H	2	1	1	Vietnam
SAMEA3233824	4	PRJEB8392	H	2	1	1	Vietnam
SAMEA3233825	4	PRJEB8392	H	2	1	1	Vietnam
SAMEA3233826	4	PRJEB8392	H	2	1	1	Vietnam
SAMEA3233827	4	PRJEB8392	H	2	1	1	Vietnam
SAMEA3233828	4	PRJEB8392	H	1	105	1	Vietnam
SAMEA3233829	4	PRJEB8392	H	2	1	1	Vietnam
SAMEA3233831	4	PRJEB8392	H	2	1	1	Vietnam
SAMEA3233832	4	PRJEB8392	H	2	1	1	Vietnam
SAMEA3233833	4	PRJEB8392	H	2	1	1	Vietnam
SAMEA3233834	4	PRJEB8392	H	2	1	1	Vietnam
SAMEA3233835	4	PRJEB8392	H	2	1	1	Vietnam
SAMEA3233836	4	PRJEB8392	H	2	1	1	Vietnam
SAMEA3233837	4	PRJEB8392	H	2	1	1	Vietnam
SAMEA3233839	4	PRJEB8392	PH	2	1	1	Vietnam
SAMEA3233840	4	PRJEB8392	PH	2	1	1	Vietnam
SAMEA3233841	4	PRJEB8392	PH	2	1	1	Vietnam
SAMEA3233842	4	PRJEB8392	PH	2	1	1	Vietnam
SAMEA3233843	4	PRJEB8392	PH	2	1	1	Vietnam
SAMEA3233845	4	PRJEB8392	PH	2	1	1	Vietnam
SAMEA3233846	4	PRJEB8392	PH	2	1	1	Vietnam
SAMEA3233847	4	PRJEB8392	PH	2	1	1	Vietnam
SAMEA3233848	4	PRJEB8392	PH	2	1	1	Vietnam
SAMEA3233849	4	PRJEB8392	PH	2	1	1	Vietnam
SAMEA3233850	4	PRJEB8392	PH	2	1	1	Vietnam

SAMEA3233851	4	PRJEB8392	PH	2	1	1	Vietnam
SAMEA3233853	4	PRJEB8392	PH	2	1	1	Vietnam
SAMEA3233854	4	PRJEB8392	PH	2	1	1	Vietnam
SAMEA3233855	4	PRJEB8392	PH	2	1	1	Vietnam
SAMEA3233856	4	PRJEB8392	PH	2	1	1	Vietnam
SAMEA3233857	4	PRJEB8392	PH	2	1	1	Vietnam
SAMEA3233858	4	PRJEB8392	PH	2	1	1	Vietnam
SAMEA3233859	4	PRJEB8392	PH	2	1	1	Vietnam
SAMEA3233860	4	PRJEB8392	PH	2	1	1	Vietnam
SAMEA3233861	4	PRJEB8392	PH	2	1	1	Vietnam
SAMEA3233862	4	PRJEB8392	PH	2	1	1	Vietnam
SAMEA3233863	4	PRJEB8392	PH	2	1	1	Vietnam
SAMEA3233864	4	PRJEB8392	PH	2	1	1	Vietnam
SAMEA3233865	4	PRJEB8392	PH	2	1	1	Vietnam
SAMEA3233866	4	PRJEB8392	PH	2	1	1	Vietnam
SAMEA3233867	4	PRJEB8392	PH	2	1	1	Vietnam
SAMEA3233868	4	PRJEB8392	PH	2	1	1	Vietnam
SAMEA3233869	4	PRJEB8392	PH	2	1	1	Vietnam
SAMEA3233870	4	PRJEB8392	PH	2	1	1	Vietnam
SAMEA3233871	4	PRJEB8392	PD	2	873	1	United Kingdom
SAMEA3233872	4	PRJEB8392	PD	1	1	1	United Kingdom
SAMEA3233873	4	PRJEB8392	PD	1	1	1	United Kingdom
SAMEA3233874	4	PRJEB8392	PD	3	27	2	United Kingdom
SAMEA3233875	4	PRJEB8392	PD	3	855	3	United Kingdom
SAMEA3233876	4	PRJEB8392	PD	4	856	2	United Kingdom
SAMEA3233877	4	PRJEB8392	PD	7	29	2	United Kingdom
SAMEA3233878	4	PRJEB8392	PD	7	29	2	United Kingdom
SAMEA3233879	4	PRJEB8392	PD	1	1	1	United Kingdom
SAMEA3233880	4	PRJEB8392	PD	1	1	1	United Kingdom
SAMEA3233881	4	PRJEB8392	PH	31	947	163	United Kingdom
SAMEA3233884	4	PRJEB8392	PH	31	858	110	United Kingdom
SAMEA3233885	4	PRJEB8392	PH	6	948	22	United Kingdom
SAMEA3233886	4	PRJEB8392	PH	31	859	111	United Kingdom
SAMEA3233887	4	PRJEB8392	PH	4	23	3	United Kingdom
SAMEA3233888	4	PRJEB8392	PH	9	Unknown	Unknown	United Kingdom
SAMEA3233890	4	PRJEB8392	PH	31	860	112	United Kingdom
SAMEA3233891	4	PRJEB8392	PH	15	875	119	United Kingdom
SAMEA3233892	4	PRJEB8392	PH	31	876	120	United Kingdom
SAMEA3233893	4	PRJEB8392	PH	2	28	2	United Kingdom
SAMEA3233894	4	PRJEB8392	PH	21	792	99	United Kingdom
SAMEA3233895	4	PRJEB8392	PH	6	949	22	United Kingdom
SAMEA3233897	4	PRJEB8392	PH	2	28	2	United Kingdom
SAMEA3233899	4	PRJEB8392	PH	24	950	164	United Kingdom
SAMEA3233900	4	PRJEB8392	PH	16	877	121	United Kingdom
SAMEA3233901	4	PRJEB8392	PD	25	861	113	United Kingdom
SAMEA3233902	4	PRJEB8392	PH	2	1	1	United Kingdom
SAMEA3233903	4	PRJEB8392	PH	16	878	122	United Kingdom
SAMEA3233904	4	PRJEB8392	PH	9	46	16	United Kingdom
SAMEA3233906	4	PRJEB8392	PH	2	28	2	United Kingdom
SAMEA3233910	4	PRJEB8392	PH	7	29	2	United Kingdom
SAMEA3233911	4	PRJEB8392	PD	16	879	123	United Kingdom
SAMEA3233912	4	PRJEB8392	PH	2	1	1	United Kingdom
SAMEA3233913	4	PRJEB8392	PH	Unknown	880	124	United Kingdom
SAMEA3233915	4	PRJEB8392	PH	21	792	99	United Kingdom
SAMEA3233916	4	PRJEB8392	PH	8	87	3	United Kingdom
SAMEA3233917	4	PRJEB8392	PH	7	29	2	United Kingdom
SAMEA3233918	4	PRJEB8392	PD	1	1	1	United Kingdom
SAMEA3233919	4	PRJEB8392	PH	21	881	17	United Kingdom
SAMEA3233920	4	PRJEB8392	PH	15	882	125	United Kingdom
SAMEA3233921	4	PRJEB8392	PH	4	862	3	United Kingdom
SAMEA3233924	4	PRJEB8392	PH	Unknown	901	137	United Kingdom
SAMEA3233926	4	PRJEB8392	PH	7	29	2	United Kingdom
SAMEA3233927	4	PRJEB8392	PH	10	902	138	United Kingdom
SAMEA3233928	4	PRJEB8392	PH	11	905	140	United Kingdom
SAMEA3233929	4	PRJEB8392	PH	2	28	2	United Kingdom
SAMEA3233930	4	PRJEB8392	PH	31	883	126	United Kingdom
SAMEA3233931	4	PRJEB8392	PH	9	863	114	United Kingdom
SAMEA3233933	4	PRJEB8392	PH	2	28	2	United Kingdom
SAMEA3233935	4	PRJEB8392	PH	11	905	140	United Kingdom
SAMEA3233937	4	PRJEB8392	PH	8	87	3	United Kingdom
SAMEA3233939	4	PRJEB8392	PH	4	884	127	United Kingdom
SAMEA3233940	4	PRJEB8392	PH	24	885	128	United Kingdom
SAMEA3233942	4	PRJEB8392	PH	8	87	3	United Kingdom
SAMEA3233943	4	PRJEB8392	PH	16	864	115	United Kingdom
SAMEA3233945	4	PRJEB8392	PH	10	893	18	United Kingdom
SAMEA3233946	4	PRJEB8392	PH	2	28	2	United Kingdom
SAMEA3233947	4	PRJEB8392	PH	31	886	129	United Kingdom
SAMEA3233948	4	PRJEB8392	PH	Unknown	897	134	United Kingdom
SAMEA3233949	4	PRJEB8392	PH	Unknown	887	17	United Kingdom
SAMEA3233950	4	PRJEB8392	PH	16	864	115	United Kingdom
SAMEA3233951	4	PRJEB8392	PH	8	87	3	United Kingdom
SAMEA3233952	4	PRJEB8392	PH	31	898	135	United Kingdom
SAMEA3233955	4	PRJEB8392	PH	12	904	139	United Kingdom
SAMEA3233956	4	PRJEB8392	PH	10	888	130	United Kingdom
SAMEA3233957	4	PRJEB8392	PH	31	889	131	United Kingdom
SAMEA3233958	4	PRJEB8392	PH	8	87	3	United Kingdom
SAMEA3233959	4	PRJEB8392	PH	31	890	132	United Kingdom
SAMEA3233960	4	PRJEB8392	PH	Unknown	899	136	United Kingdom
SAMEA3233963	4	PRJEB8392	PH	15	875	119	United Kingdom
SAMEA3233965	4	PRJEB8392	PH	2	28	2	United Kingdom
SAMEA3233967	4	PRJEB8392	PH	2	28	2	United Kingdom
SAMEA3233969	4	PRJEB8392	PH	10	866	116	United Kingdom
SAMEA3233971	4	PRJEB8392	PD	2	891	1	United Kingdom
SAMEA3233972	4	PRJEB8392	PD	3	31	2	United Kingdom
SAMEA3233974	4	PRJEB8392	PD	7	29	2	United Kingdom
SAMEA3233975	4	PRJEB8392	PD	2	873	1	United Kingdom
SAMEA3233976	4	PRJEB8392	PD	2	28	2	United Kingdom
SAMEA3233977	4	PRJEB8392	PD	7	29	2	United Kingdom
SAMEA3233978	4	PRJEB8392	PD	2	1	1	United Kingdom
SAMEA3233979	4	PRJEB8392	PD	2	1	1	United Kingdom
SAMEA3233980	4	PRJEB8392	PD	2	1	1	United Kingdom
SAMEA3233981	4	PRJEB8392	PD	1	1	1	United Kingdom
SAMEA3233982	4	PRJEB8392	PD	2	1	1	United Kingdom
SAMEA3233983	4	PRJEB8392	PD	1	910	142	United Kingdom
SAMEA3233984	4	PRJEB8392	PD	3	27	2	United Kingdom
SAMEA3233985	4	PRJEB8392	PD	3	27	2	United Kingdom
SAMEA3233986	4	PRJEB8392	PD	8	87	3	United Kingdom
SAMEA3233987	4	PRJEB8392	PD	2	1	1	United Kingdom
SAMEA3233988	4	PRJEB8392	PD	7	29	2	United Kingdom
SAMEA3233989	4	PRJEB8392	H	2	1	1	Vietnam
SAMEA3233990	4	PRJEB8392	PD	3	27	2	United Kingdom
SAMEA3233991	4	PRJEB8392	PD	7	907	5	United Kingdom
SAMEA3233992	4	PRJEB8392	PD	2	1	1	United Kingdom
SAMEA3233993	4	PRJEB8392	PD	2	1	1	United Kingdom
SAMEA3233994	4	PRJEB8392	PD	2	1	1	United Kingdom
SAMEA3233995	4	PRJEB8392	PD	2	1	1	United Kingdom
SAMEA3233996	4	PRJEB8392	PD	1	1	1	United Kingdom
SAMEA3233997	4	PRJEB8392	PD	4	908	141	United Kingdom
SAMEA3233998	4	PRJEB8392	PD	1	1	1	United Kingdom

SAMEA3233999	4	PRJEB8392	H	2	144	1	Vietnam
SAMEA3234001	4	PRJEB8392	PD	2	1	1	United Kingdom
SAMEA3234002	4	PRJEB8392	PD	1	1	1	United Kingdom
SAMEA3234003	4	PRJEB8392	PD	2	28	2	United Kingdom
SAMEA3234004	4	PRJEB8392	PD	8	867	2	United Kingdom
SAMEA3234005	4	PRJEB8392	PD	2	1	1	United Kingdom
SAMEA3234006	4	PRJEB8392	PD	8	87	3	United Kingdom
SAMEA3234007	4	PRJEB8392	PD	1	1	1	United Kingdom
SAMEA3234008	4	PRJEB8392	PD	1	1	1	United Kingdom
SAMEA3234009	4	PRJEB8392	PD	4	911	0	United Kingdom
SAMEA3234010	4	PRJEB8392	PD	4	911	0	United Kingdom
SAMEA3234011	4	PRJEB8392	PD	4	911	0	United Kingdom
SAMEA3234012	4	PRJEB8392	PD	2	28	2	United Kingdom
SAMEA3234013	4	PRJEB8392	PD	4	23	3	United Kingdom
SAMEA3234014	4	PRJEB8392	PD	7	29	2	United Kingdom
SAMEA3234015	4	PRJEB8392	PD	3	868	3	United Kingdom
SAMEA3234016	4	PRJEB8392	PD	2	25	2	United Kingdom
SAMEA3234017	4	PRJEB8392	PD	2	1	1	United Kingdom
SAMEA3234018	4	PRJEB8392	PD	2	1	1	United Kingdom
SAMEA3234019	4	PRJEB8392	PD	8	908	141	United Kingdom
SAMEA3234020	4	PRJEB8392	PD	3	31	2	United Kingdom
SAMEA3234022	4	PRJEB8392	PD	15	912	143	United Kingdom
SAMEA3234023	4	PRJEB8392	PD	2	1	1	United Kingdom
SAMEA3234024	4	PRJEB8392	PD	2	25	2	United Kingdom
SAMEA3234025	4	PRJEB8392	PD	2	1	1	United Kingdom
SAMEA3234026	4	PRJEB8392	PD	2	1	1	United Kingdom
SAMEA3234027	4	PRJEB8392	PD	16	871	117	United Kingdom
SAMEA3234028	4	PRJEB8392	PD	8	87	3	United Kingdom
SAMEA3234029	4	PRJEB8392	PD	2	1	1	United Kingdom
SAMEA3234030	4	PRJEB8392	PD	2	909	1	United Kingdom
SAMEA3234031	4	PRJEB8392	PD	2	1	1	United Kingdom
SAMEA3234032	4	PRJEB8392	H	2	1	1	Vietnam
SAMEA3234033	4	PRJEB8392	H	2	1	1	Vietnam
SAMEA3234034	4	PRJEB8392	H	2	1	1	Vietnam
SAMEA3234035	4	PRJEB8392	H	2	1	1	Vietnam
SAMEA3234037	4	PRJEB8392	H	2	1	1	Vietnam
SAMEA3234038	4	PRJEB8392	H	2	1	1	Vietnam
SAMEA3234039	4	PRJEB8392	H	2	1	1	Vietnam
SAMEA3234040	4	PRJEB8392	H	2	1	1	Vietnam
SAMEA3234041	4	PRJEB8392	H	2	1	1	Vietnam
SAMEA3234042	4	PRJEB8392	H	2	1	1	Vietnam
SAMEA3234043	4	PRJEB8392	H	2	1	1	Vietnam
SAMEA3234044	4	PRJEB8392	H	2	1	1	Vietnam
SAMEA3234045	4	PRJEB8392	H	2	1	1	Vietnam
SAMEA3234046	4	PRJEB8392	H	2	1	1	Vietnam
SAMEA3234047	4	PRJEB8392	H	2	1	1	Vietnam
SAMEA3234048	4	PRJEB8392	H	2	1	1	Vietnam
SAMEA3234050	4	PRJEB8392	H	2	1	1	Vietnam
SAMEA3234051	4	PRJEB8392	H	2	1	1	Vietnam
SAMEA3234052	4	PRJEB8392	H	2	1	1	Vietnam
SAMEA3234053	4	PRJEB8392	H	2	1	1	Vietnam
SAMEA3234054	4	PRJEB8392	H	2	1	1	Vietnam
SAMEA3234055	4	PRJEB8392	H	2	1	1	Vietnam
SAMEA3234057	4	PRJEB8392	H	2	1	1	Vietnam
SAMEA3234058	4	PRJEB8392	H	2	1	1	Vietnam
SAMEA3234059	4	PRJEB8392	H	2	1	1	Vietnam
SAMEA3234060	4	PRJEB8392	H	2	1	1	Vietnam
SAMEA3234061	4	PRJEB8392	H	2	1	1	Vietnam
SAMEA3234062	4	PRJEB8392	H	2	107	1	Vietnam
SAMEA3234063	4	PRJEB8392	H	2	1	1	Vietnam
SAMEA3234064	4	PRJEB8392	H	2	1	1	Vietnam
SAMEA3234065	4	PRJEB8392	H	2	869	1	Vietnam
SAMEA3234066	4	PRJEB8392	H	2	1	1	Vietnam
SAMEA3234067	4	PRJEB8392	H	2	1	1	Vietnam
SAMEA3234068	4	PRJEB8392	H	2	1	1	Vietnam
SAMEA3234069	4	PRJEB8392	H	2	1	1	Vietnam
SAMEA3234070	4	PRJEB8392	H	2	1	1	Vietnam
SAMEA3234072	4	PRJEB8392	H	2	1	1	Vietnam
SAMEA3234073	4	PRJEB8392	H	2	144	1	Vietnam
SAMEA3234074	4	PRJEB8392	H	2	1	1	Vietnam
SAMEA3234075	4	PRJEB8392	PD	2	1	1	United Kingdom
SAMEA3234076	4	PRJEB8392	PD	9	790	98	United Kingdom
SAMEA3234077	4	PRJEB8392	PD	2	1	1	United Kingdom
SAMEA3234078	4	PRJEB8392	PD	2	1	1	United Kingdom
SAMEA3234079	4	PRJEB8392	PD	1	1	1	United Kingdom
SAMEA3234080	4	PRJEB8392	PD	2	1	1	United Kingdom
SAMEA3234081	4	PRJEB8392	PD	2	1	1	United Kingdom
SAMEA3234082	4	PRJEB8392	PD	1	1	1	United Kingdom
SAMEA3234083	4	PRJEB8392	PH	Unknown	915	146	United Kingdom
SAMEA3234084	4	PRJEB8392	PD	2	28	2	United Kingdom
SAMEA3234085	4	PRJEB8392	PD	2	1	1	United Kingdom
SAMEA3234086	4	PRJEB8392	PD	23	913	144	United Kingdom
SAMEA3234088	4	PRJEB8392	PD	1	1	1	United Kingdom
SAMEA3234089	4	PRJEB8392	PD	2	1	1	United Kingdom
SAMEA3234090	4	PRJEB8392	PD	12	914	145	United Kingdom
SAMEA3234091	4	PRJEB8392	PD	2	28	2	United Kingdom
SAMEA3234093	4	PRJEB8392	PD	1	1	1	United Kingdom
SAMEA3234094	4	PRJEB8392	PD	8	916	18	United Kingdom
SAMEA3234095	4	PRJEB8392	PD	2	1	1	United Kingdom
SAMEA3234096	4	PRJEB8392	PD	2	1	1	United Kingdom
SAMEA3234097	4	PRJEB8392	PD	1	1	1	United Kingdom
SAMEA3234098	4	PRJEB8392	PD	2	28	2	United Kingdom
SAMEA3234099	4	PRJEB8392	PD	3	108	0	United Kingdom
SAMEA3234100	4	PRJEB8392	H	2	1	1	Vietnam
SAMEA3234101	4	PRJEB8392	H	2	144	1	Vietnam
SAMEA3234102	4	PRJEB8392	H	2	1	1	Vietnam
SAMEA3234103	4	PRJEB8392	H	2	1	1	Vietnam
SAMEA3234104	4	PRJEB8392	H	2	1	1	Vietnam
SAMEA3234105	4	PRJEB8392	H	2	1	1	Vietnam
SAMEA3234106	4	PRJEB8392	H	2	1	1	Vietnam
SAMEA3234107	4	PRJEB8392	H	2	951	1	Vietnam
SAMEA3234108	4	PRJEB8392	H	2	1	1	Vietnam
SAMEA3234109	4	PRJEB8392	H	2	1	1	Vietnam
SAMEA3234111	4	PRJEB8392	H	2	1	1	Vietnam
SAMEA3234112	4	PRJEB8392	H	2	1	1	Vietnam
SAMEA3234113	4	PRJEB8392	H	2	1	1	Vietnam
SAMEA3234114	4	PRJEB8392	H	2	1	1	Vietnam
SAMEA3234115	4	PRJEB8392	H	2	1	1	Vietnam
SAMEA3234116	4	PRJEB8392	H	2	1	1	Vietnam
SAMEA3234117	4	PRJEB8392	H	2	1	1	Vietnam
SAMEA3234118	4	PRJEB8392	H	2	1	1	Vietnam
SAMEA3234119	4	PRJEB8392	H	2	1	1	Vietnam
SAMEA3234120	4	PRJEB8392	H	2	1	1	Vietnam
SAMEA3234121	4	PRJEB8392	H	2	1	1	Vietnam
SAMEA3234122	4	PRJEB8392	H	2	1	1	Vietnam
SAMEA3234123	4	PRJEB8392	H	2	1	1	Vietnam
SAMEA3234124	4	PRJEB8392	H	2	1	1	Vietnam
SAMEA3234125	4	PRJEB8392	H	2	1	1	Vietnam
SAMEA3234126	4	PRJEB8392	H	2	1	1	Vietnam

SAMEA1530134	5	PRJEB2712	H	Unknown	Unknown	Unknown	United Kingdom
SAMEA2272312	6	PRJEA32239	H	2	7	1	China
SAMEA2272697	6	PRJEA32237	H	2	1	1	Vietnam
SAMEA3138299	6	PRJNA352	PD	2	1	1	United Kingdom
SAMN02441756	7	PRJNA12417	PD	2	Unknown	Unknown	Canada
SAMN02469350	8	PRJNA17157	PH	2	28	2	China
SAMN02469503	9	PRJNA171440	PD	2	1	1	Netherlands
SAMN02469504	9	PRJNA171441	PH	8	308	3	China
SAMN02469506	9	PRJNA171447	PD	1	13	9	Denmark
SAMN02469507	9	PRJNA171448	PH	12	306	53	China
SAMN02469508	9	PRJNA171449	PH	7	17	6	China
SAMN02469509	9	PRJNA171451	PH	31	309	54	China
SAMN02469510	9	PRJNA171456	PH	Unknown	328	65	China
SAMN02469511	9	PRJNA171457	PH	10	311	56	China
SAMN02469512	9	PRJNA171458	PH	27	312	57	China
SAMN02469513	9	PRJNA171460	PH	Unknown	314	58	China
SAMN02469514	9	PRJNA171461	PH	Unknown	315	59	China
SAMN02469515	9	PRJNA171465	PH	11	318	11	China
SAMN02469516	9	PRJNA171468	PH	2	28	2	China
SAMN02469517	9	PRJNA171477	PH	11	321	63	China
SAMN02469518	9	PRJNA171478	PH	2	1	1	China
SAMN02469519	9	PRJNA171469	PH	29	330	12	China
SAMN02469520	9	PRJNA171482	PH	29	334	68	China
SAMN02469521	9	PRJNA171419	PD	6	55	25	Netherlands
SAMN02469522	9	PRJNA171420	PD	2	56	27	Netherlands
SAMN02469523	9	PRJNA171429	PD	12	775	95	Netherlands
SAMN02469524	9	PRJNA171430	PD	2	25	2	Canada
SAMN02469525	9	PRJNA171435	PD	29	92	41	Canada
SAMN02469526	9	PRJNA171445	PD	24	68	28	Canada
SAMN02469527	9	PRJNA171462	PH	12	316	60	China
SAMN02469529	9	PRJNA171432	PD	25	69	29	Canada
SAMN02469530	9	PRJNA171454	PH	2	1	1	China
SAMN02469531	9	PRJNA171474	PH	12	313	11	China
SAMN02469532	9	PRJNA171480	PH	Unknown	332	67	China
SAMN02469533	9	PRJNA171483	PH	Unknown	305	52	China
SAMN02469534	9	PRJNA171422	PD	10	78	36	Denmark
SAMN02469535	9	PRJNA171424	PD	4	54	8	Netherlands
SAMN02469536	9	PRJNA171425	PD	7	29	2	Denmark
SAMN02469538	9	PRJNA171439	PH	18	79	37	Canada
SAMN02469539	9	PRJNA171443	PH	19	76	34	Canada
SAMN02469540	9	PRJNA171450	PH	8	308	3	China
SAMN02469541	9	PRJNA171455	PH	Unknown	310	55	China
SAMN02469543	9	PRJNA171475	PH	11	345	69	China
SAMN02469544	9	PRJNA171416	PH	21	481	81	Canada
SAMN02469545	9	PRJNA171446	PD	28	75	33	Canada
SAMN02469546	9	PRJNA171464	PH	Unknown	317	61	China
SAMN02469547	9	PRJNA171466	PH	Unknown	952	165	China
SAMN02469548	9	PRJNA171473	PH	Unknown	953	166	China
SAMN02469549	9	PRJNA171481	PH	Unknown	872	118	China
SAMN02469550	9	PRJNA171421	PD	16	73	32	Netherlands
SAMN02469551	9	PRJNA171423	PD	3	35	2	Netherlands
SAMN02469552	9	PRJNA171431	PD	23	483	0	Canada
SAMN02469554	9	PRJNA171434	PD	27	72	31	Canada
SAMN02469555	9	PRJNA171436	PD	30	77	35	Canada
SAMN02469556	9	PRJNA171452	PH	31	327	64	China
SAMN02469557	9	PRJNA171453	PH	2	28	2	China
SAMN02469558	9	PRJNA171470	PH	11	318	11	China
SAMN02469559	9	PRJNA171471	PH	29	319	62	China
SAMN02469560	9	PRJNA171476	PH	7	32	5	China
SAMN02469562	9	PRJNA171438	PH	17	76	34	Canada
SAMN02469564	9	PRJNA171459	PH	12	313	11	China
SAMN02469565	9	PRJNA171463	PH	11	304	51	China
SAMN02469566	9	PRJNA171467	PH	Unknown	329	12	China
SAMN02470631	9	PRJNA171414	H	1	6	1	Netherlands
SAMN02470632	9	PRJNA171418	PD	9	82	39	Denmark
SAMN02470634	9	PRJNA171411	PD	5	53	8	Netherlands
SAMN02470635	9	PRJNA171415	PD	8	87	3	Netherlands
SAMN02470636	9	PRJNA171412	H	2	1	1	Germany
SAMN02470638	9	PRJNA171413	PD	11	91	40	Denmark
SAMN02470639	9	PRJNA171409	PD	2	7	1	China
SAMN07176385	9	PRJNA197484	PH	Unknown	432	73	China
SAMN07176400	9	PRJNA197466	PH	Unknown	432	73	China
SAMN07176413	9	PRJNA197472	PH	Unknown	453	78	China
SAMN07176419	9	PRJNA197465	PH	Unknown	451	77	China
SAMN02469902	10	PRJNA63545	PD	Unknown	776	14	China
SAMN02603318	10	PRJNA63483	PD	2	7	1	China
SAMN02603120	11	PRJNA171404	PH	16	664	87	China
SAMN02603121	12	PRJNA213888	PH	3	35	2	China
SAMN02603319	13	PRJNA65245	PD	3	35	2	China
SAMN02603326	14	PRJNA61797	PD	1	7	1	China
SAMN02603632	15	PRJNA178071	PD	2	7	1	China
SAMN02603633	16	PRJNA206218	PH	2	19	3	Netherlands
SAMN02603644	17	PRJNA18737	H	2	1	1	China
SAMN02905150	18	PRJNA254762	PD	4	54	8	Denmark
SAMN03485884	19	PRJNA279995	PD	2	Unknown	Unknown	USA
SAMN03485885	19	PRJNA279995	PD	2	25	2	USA
SAMN03485886	19	PRJNA279995	PD	Unknown	28	2	USA
SAMN03485887	19	PRJNA279995	PD	2	1	1	USA
SAMN03485888	19	PRJNA279995	PD	Unknown	76	34	USA
SAMN03485890	19	PRJNA279995	PD	2	1	1	USA
SAMN03485891	19	PRJNA279995	PD	2	787	2	USA
SAMN04956488	19	PRJNA320740	PH	Unknown	785	15	USA
SAMN04956520	19	PRJNA320741	PD	19	76	34	USA
SAMN03652160	20	PRJNA283528	PD	Unknown	28	2	Canada
SAMN03652161	20	PRJNA283528	PD	2	1180	2	USA
SAMN03652162	20	PRJNA283528	PD	2	28	2	USA
SAMN03652163	20	PRJNA283528	PD	2	28	2	USA
SAMN03652164	20	PRJNA283528	PD	2	28	2	USA
SAMN03652165	20	PRJNA283528	PD	2	28	2	USA
SAMN03652166	20	PRJNA283528	PD	2	28	2	USA
SAMN03652167	20	PRJNA283528	PD	2	28	2	USA
SAMN03652168	20	PRJNA283528	PD	2	28	2	USA
SAMN03652169	20	PRJNA283528	PD	2	28	2	USA
SAMN03652170	20	PRJNA283528	PD	2	28	2	USA
SAMN03652171	20	PRJNA283528	PD	2	28	2	USA
SAMN03652172	20	PRJNA283528	PD	2	28	2	USA
SAMN03652173	20	PRJNA283528	PD	2	28	2	USA
SAMN03652174	20	PRJNA283528	PD	2	28	2	Japan
SAMN03652175	20	PRJNA283528	PD	2	28	2	Japan
SAMN03652176	20	PRJNA283528	PD	2	28	2	Japan
SAMN03652177	20	PRJNA283528	PD	2	28	2	Japan
SAMN03652178	20	PRJNA283528	PD	2	28	2	Japan
SAMN03652179	20	PRJNA283528	PD	2	28	2	Japan
SAMN03652180	20	PRJNA283528	PD	2	28	2	Japan
SAMN03652181	20	PRJNA283528	PD	2	28	2	Japan
SAMN03652182	20	PRJNA283528	PD	2	28	2	Japan
SAMN03652183	20	PRJNA283528	PD	2	28	2	Japan
SAMN03652184	20	PRJNA283528	PD	2	28	2	Japan
SAMN03652185	20	PRJNA283528	PD	2	28	2	Japan

SAMN03652186	20	PRJNA283528	PD	2	28	2	Japan
SAMN03652187	20	PRJNA283528	PD	2	28	2	Japan
SAMN03652188	20	PRJNA283528	PD	2	28	2	Japan
SAMN03652189	20	PRJNA283528	H	2	28	2	Thailand
SAMN03652190	20	PRJNA283528	PD	2	28	2	Canada
SAMN03652191	20	PRJNA283528	PD	2	28	2	Canada
SAMN03652192	20	PRJNA283528	PD	2	28	2	Canada
SAMN03652193	20	PRJNA283528	PD	2	28	2	Canada
SAMN03652194	20	PRJNA283528	PD	2	28	2	Canada
SAMN03652195	20	PRJNA283528	PD	2	28	2	Canada
SAMN03652196	20	PRJNA283528	PD	2	28	2	Canada
SAMN03652197	20	PRJNA283528	PD	2	28	2	Canada
SAMN03652198	20	PRJNA283528	PD	2	28	2	Canada
SAMN03652199	20	PRJNA283528	PD	Unknown	28	2	USA
SAMN03652200	20	PRJNA283528	PD	Unknown	28	2	USA
SAMN03652201	20	PRJNA283528	PD	2	620	2	Canada
SAMN03652202	20	PRJNA283528	PD	2	28	2	Canada
SAMN03652203	20	PRJNA283528	PD	2	28	2	Canada
SAMN03652204	20	PRJNA283528	PD	2	28	2	Canada
SAMN03652205	20	PRJNA283528	PD	2	28	2	Canada
SAMN03652206	20	PRJNA283528	PD	2	28	2	Canada
SAMN03652207	20	PRJNA283528	PD	2	1180	2	Canada
SAMN03652208	20	PRJNA283528	PD	Unknown	28	2	Canada
SAMN03652209	20	PRJNA283528	PD	2	28	2	Canada
SAMN03652210	20	PRJNA283528	PD	Unknown	28	2	Canada
SAMN03652427	20	PRJNA283547	PD	2	28	2	Canada
SAMN04044079	21	PRJNA295196	PH	2	28	2	Canada
SAMN04156190	22	PRJNA301006	PD	2	25	2	Canada
SAMN04231414	22	PRJNA301006	H	Unknown	25	2	Canada
SAMN04231434	22	PRJNA301006	PD	2	25	2	USA
SAMN04231435	22	PRJNA301006	PD	2	25	2	USA
SAMN04231436	22	PRJNA301006	H	2	25	2	Thailand
SAMN04231437	22	PRJNA301006	H	2	25	2	Thailand
SAMN04231438	22	PRJNA301006	H	2	25	2	Thailand
SAMN04231439	22	PRJNA301006	H	2	25	2	Thailand
SAMN04231440	22	PRJNA301006	H	2	25	2	Thailand
SAMN04231441	22	PRJNA301006	H	2	25	2	Thailand
SAMN04231442	22	PRJNA301006	H	2	25	2	Thailand
SAMN04231443	22	PRJNA301006	PD	2	25	2	Canada
SAMN04231444	22	PRJNA301006	PD	2	25	2	Canada
SAMN04231445	22	PRJNA301006	PD	2	25	2	Canada
SAMN04231446	22	PRJNA301006	PD	2	25	2	Canada
SAMN04231447	22	PRJNA301006	PD	2	25	2	Canada
SAMN04231448	22	PRJNA301006	PD	2	25	2	Canada
SAMN04231449	22	PRJNA301006	PD	2	25	2	Canada
SAMN04231450	22	PRJNA301006	PD	2	Unknown	Unknown	Canada
SAMN04231451	22	PRJNA301006	PD	2	25	2	Canada
SAMN04231452	22	PRJNA301006	PD	2	25	2	Canada
SAMN04231453	22	PRJNA301006	PD	2	25	2	Canada
SAMN04231454	22	PRJNA301006	PD	2	25	2	Canada
SAMN04231455	22	PRJNA301006	PD	2	25	2	Canada
SAMN04231456	22	PRJNA301006	PD	2	25	2	Canada
SAMN04231457	22	PRJNA301006	PD	2	25	2	Canada
SAMN04231458	22	PRJNA301006	PD	2	25	2	Canada
SAMN04231459	22	PRJNA301006	PD	2	25	2	Canada
SAMN04231460	22	PRJNA301006	PD	2	25	2	Canada
SAMN04231461	22	PRJNA301006	PD	2	25	2	Canada
SAMN04231462	22	PRJNA301006	PD	2	25	2	Canada
SAMN04231463	22	PRJNA301006	PD	2	25	2	Canada
SAMN04231464	22	PRJNA301006	PD	2	25	2	Canada
SAMN04231465	22	PRJNA301006	PD	2	25	2	Canada
SAMN04231466	22	PRJNA301006	PD	2	25	2	Canada
SAMN04231467	22	PRJNA301006	PD	2	25	2	Canada
SAMN04231468	22	PRJNA301006	PD	2	25	2	Canada
SAMN04231469	22	PRJNA301006	PD	2	25	2	Canada
SAMN04231470	22	PRJNA301006	PD	2	25	2	Canada
SAMN04231471	22	PRJNA301006	H	2	25	2	USA
SAMN04231472	22	PRJNA301006	PD	2	25	2	Canada
SAMN04231473	22	PRJNA301006	PD	2	25	2	Canada
SAMN04231474	22	PRJNA301006	H	2	25	2	Thailand
SAMN04231475	22	PRJNA301006	PD	2	25	2	Canada
SAMN04231476	22	PRJNA301006	PD	2	25	2	Canada
SAMN04231477	22	PRJNA301006	PD	2	25	2	Canada
SAMN04231478	22	PRJNA301006	PD	2	25	2	Canada
SAMN04231479	22	PRJNA301006	PD	2	25	2	Canada
SAMN04231480	22	PRJNA301006	PD	2	25	2	Canada
SAMN04231481	22	PRJNA301006	PD	2	25	2	Canada
SAMN04231482	22	PRJNA301006	PD	2	25	2	Canada
SAMN04376513	23	PRJNA307190	PD	1	1	1	Canada
SAMN04376514	23	PRJNA307190	PD	1	1	1	Canada
SAMN04376515	23	PRJNA307190	PD	1	1	1	Canada
SAMN04376516	23	PRJNA307190	PD	1	13	9	Canada
SAMN04376517	23	PRJNA307190	PD	1	1	1	Canada
SAMN04376518	23	PRJNA307190	PD	1	1	1	Canada
SAMN04376519	23	PRJNA307190	PD	1	1	1	Canada
SAMN04376520	23	PRJNA307190	PD	2	28	2	Canada
SAMN04376521	23	PRJNA307190	PD	2	28	2	Canada
SAMN04376522	23	PRJNA307190	PD	2	28	2	Canada
SAMN04376523	23	PRJNA307190	PD	2	28	2	Canada
SAMN04376524	23	PRJNA307190	PD	2	28	2	Canada
SAMN04376525	23	PRJNA307190	PD	2	28	2	Canada
SAMN04376526	23	PRJNA307190	PD	2	28	2	Canada
SAMN04376527	23	PRJNA307190	PD	2	25	2	Canada
SAMN04376528	23	PRJNA307190	PD	2	25	2	Canada
SAMN04376529	23	PRJNA307190	PD	2	28	2	Canada
SAMN04376530	23	PRJNA307190	PD	2	28	2	Canada
SAMN04376531	23	PRJNA307190	PD	2	25	2	Canada
SAMN04376532	23	PRJNA307190	PD	2	28	2	Canada
SAMN04376533	23	PRJNA307190	PD	2	25	2	Canada
SAMN04376534	23	PRJNA307190	PD	3	108	0	Canada
SAMN04376535	23	PRJNA307190	PD	3	1187	0	Canada
SAMN04376536	23	PRJNA307190	PD	3	117	2	Canada
SAMN04376537	23	PRJNA307190	PD	3	27	2	Canada
SAMN04376538	23	PRJNA307190	PD	3	27	2	Canada
SAMN04376539	23	PRJNA307190	PD	4	94	0	Canada
SAMN04376540	23	PRJNA307190	PD	4	1175	0	Canada
SAMN04376541	23	PRJNA307190	PD	4	94	0	Canada
SAMN04376542	23	PRJNA307190	PD	4	977	0	Canada
SAMN04376543	23	PRJNA307190	PD	4	94	0	Canada
SAMN04376544	23	PRJNA307190	PD	4	977	0	Canada
SAMN04376545	23	PRJNA307190	PD	4	94	0	Canada
SAMN04376546	23	PRJNA307190	PD	5	94	0	Canada
SAMN04376547	23	PRJNA307190	PD	5	89	3	Canada
SAMN04376548	23	PRJNA307190	PD	5	53	8	Canada
SAMN04376549	23	PRJNA307190	PD	5	235	47	Canada
SAMN04376550	23	PRJNA307190	PD	5	Unknown	Unknown	Canada
SAMN04376551	23	PRJNA307190	PD	5	Unknown	Unknown	Canada
SAMN04376552	23	PRJNA307190	PD	5	1175	0	Canada
SAMN04376553	23	PRJNA307190	PD	6	55	25	Canada

SAMN04376554	23	PRJNA307190	PD	7	89	3	Canada
SAMN04376555	23	PRJNA307190	PD	7	225	5	Canada
SAMN04376556	23	PRJNA307190	PD	7	89	3	Canada
SAMN04376557	23	PRJNA307190	PD	7	94	0	Canada
SAMN04376558	23	PRJNA307190	PD	7	94	0	Canada
SAMN04376559	23	PRJNA307190	PD	7	1175	0	Canada
SAMN04376560	23	PRJNA307190	PD	7	32	5	Canada
SAMN04376561	23	PRJNA307190	PD	8	87	3	Canada
SAMN04376562	23	PRJNA307190	PD	8	87	3	Canada
SAMN04376563	23	PRJNA307190	PD	8	Unknown	Unknown	Canada
SAMN04376565	23	PRJNA307190	PD	8	87	3	Canada
SAMN04376566	23	PRJNA307190	PD	8	87	3	Canada
SAMN04376567	23	PRJNA307190	PD	9	Unknown	Unknown	Canada
SAMN04376568	23	PRJNA307190	PD	9	Unknown	Unknown	Canada
SAMN04376569	23	PRJNA307190	PD	10	Unknown	Unknown	Canada
SAMN04376570	23	PRJNA307190	PD	9	16	4	Canada
SAMN04376571	23	PRJNA307190	PD	9	16	4	Canada
SAMN04376572	23	PRJNA307190	PD	9	16	4	Canada
SAMN04376573	23	PRJNA307190	PD	9	16	4	Canada
SAMN04376574	23	PRJNA307190	PD	25	Unknown	Unknown	Canada
SAMN04376575	23	PRJNA307190	PD	10	Unknown	Unknown	Canada
SAMN04376577	23	PRJNA307190	PD	13	Unknown	Unknown	Canada
SAMN04376578	23	PRJNA307190	PD	1	1	1	Canada
SAMN04376579	23	PRJNA307190	PD	1	1	1	Canada
SAMN04376580	23	PRJNA307190	PD	1	Unknown	Unknown	Canada
SAMN04376581	23	PRJNA307190	PD	1	1	1	Canada
SAMN04376582	23	PRJNA307190	PD	1	1	1	Canada
SAMN04376583	23	PRJNA307190	PD	1	1	1	Canada
SAMN04376584	23	PRJNA307190	PD	1	1	1	Canada
SAMN04376585	23	PRJNA307190	PD	16	Unknown	Unknown	Canada
SAMN04376586	23	PRJNA307190	PD	16	Unknown	Unknown	Canada
SAMN04376587	23	PRJNA307190	PD	16	Unknown	Unknown	Canada
SAMN04376588	23	PRJNA307190	PD	16	Unknown	Unknown	Canada
SAMN04376589	23	PRJNA307190	PD	16	Unknown	Unknown	Canada
SAMN04376590	23	PRJNA307190	PD	16	Unknown	Unknown	Canada
SAMN04376591	23	PRJNA307190	PD	16	73	32	Canada
SAMN04376592	23	PRJNA307190	PD	17	76	34	Canada
SAMN04376593	23	PRJNA307190	PD	17	76	34	Canada
SAMN04376594	23	PRJNA307190	PD	18	778	96	Canada
SAMN04376595	23	PRJNA307190	PD	18	76	34	Canada
SAMN04376596	23	PRJNA307190	PD	19	76	34	Canada
SAMN04376597	23	PRJNA307190	PD	19	76	34	Canada
SAMN04376598	23	PRJNA307190	PD	19	778	96	Canada
SAMN04376600	23	PRJNA307190	PD	23	108	0	Canada
SAMN04376601	23	PRJNA307190	PD	23	483	0	Canada
SAMN04376602	23	PRJNA307190	PD	23	108	0	Canada
SAMN04376603	23	PRJNA307190	PD	23	108	0	Canada
SAMN04376604	23	PRJNA307190	PD	24	68	28	Canada
SAMN04376605	23	PRJNA307190	PD	24	221	10	Canada
SAMN04376606	23	PRJNA307190	PD	24	234	10	Canada
SAMN04376607	23	PRJNA307190	PD	24	Unknown	Unknown	Canada
SAMN04376608	23	PRJNA307190	PD	27	Unknown	Unknown	Canada
SAMN04376609	23	PRJNA307190	PD	27	Unknown	Unknown	Canada
SAMN04376610	23	PRJNA307190	PD	28	Unknown	Unknown	Canada
SAMN04376611	23	PRJNA307190	PD	28	Unknown	Unknown	Canada
SAMN04376613	23	PRJNA307190	PD	28	Unknown	Unknown	Canada
SAMN04376614	23	PRJNA307190	PD	28	Unknown	Unknown	Canada
SAMN04376615	23	PRJNA307190	PD	28	Unknown	Unknown	Canada
SAMN04376616	23	PRJNA307190	PD	21	Unknown	Unknown	Canada
SAMN04376617	23	PRJNA307190	PD	29	Unknown	Unknown	Canada
SAMN04376618	23	PRJNA307190	PD	29	Unknown	Unknown	Canada
SAMN04376619	23	PRJNA307190	PD	30	Unknown	Unknown	Canada
SAMN04376620	23	PRJNA307190	PD	30	Unknown	Unknown	Canada
SAMN04376621	23	PRJNA307190	PD	30	Unknown	Unknown	Canada
SAMN04376622	23	PRJNA307190	PD	30	Unknown	Unknown	Canada
SAMN04376623	23	PRJNA307190	PD	30	Unknown	Unknown	Canada
SAMN04376624	23	PRJNA307190	PD	30	Unknown	Unknown	Canada
SAMN04376625	23	PRJNA307190	PD	30	Unknown	Unknown	Canada
SAMN04376626	23	PRJNA307190	PD	31	Unknown	Unknown	Canada
SAMN04376627	23	PRJNA307190	PD	31	Unknown	Unknown	Canada
SAMN04376628	23	PRJNA307190	PD	31	Unknown	Unknown	Canada
SAMN04376629	23	PRJNA307190	PD	9	Unknown	Unknown	Canada
SAMN04376630	23	PRJNA307190	PD	9	Unknown	Unknown	Canada
SAMN04376631	23	PRJNA307190	PD	9	788	97	Canada
SAMN04376632	23	PRJNA307190	PD	11	91	40	Denmark
SAMN04376633	23	PRJNA307190	PD	15	81	38	Netherlands
SAMN04908324	23	PRJNA307190	PD	8	87	3	Canada
SAMN04884928	24	PRJNA319315	PD	9	243	48	China
SAMN08040402	25	PRJNA419012	PD	Unknown	383	14	China
SAMN08102920	25	PRJNA419988	PH	Unknown	475	79	China
SAMN08103192	25	PRJNA420006	PH	Unknown	264	50	China
SAMN08295836	26	PRJNA428542	PH	16	920	149	China
SAMN08295838	26	PRJNA428542	PH	16	920	149	China
SAMN08295839	26	PRJNA428542	PH	16	920	149	China
SAMN08295840	26	PRJNA428542	PH	16	920	149	China
SAMN08295841	26	PRJNA428542	PH	16	920	149	China
SAMN08295842	26	PRJNA428542	PH	16	920	149	China
SAMN08295843	26	PRJNA428542	PH	16	920	149	China
SAMN08295844	26	PRJNA428542	PH	31	Unknown	Unknown	China
SAMN08295845	26	PRJNA428542	PH	16	920	149	China
SAMN08295846	26	PRJNA428542	PH	16	920	149	China
SAMN08295847	26	PRJNA428542	PH	16	920	149	China
SAMN08295848	26	PRJNA428542	PH	31	Unknown	Unknown	China
SAMN08295849	26	PRJNA428542	PH	16	920	149	China
SAMN08295850	26	PRJNA428542	PH	16	920	149	China
SAMN08295851	26	PRJNA428542	PH	Unknown	Unknown	Unknown	China
SAMN08295852	26	PRJNA428542	PH	15	Unknown	Unknown	China
SAMN08295853	26	PRJNA428542	PH	16	920	149	China
SAMN08295854	26	PRJNA428542	PH	16	920	149	China
SAMN08295855	26	PRJNA428542	PH	16	920	149	China
SAMN08295856	26	PRJNA428542	PH	9	Unknown	Unknown	China
SAMN08295857	26	PRJNA428542	PH	Unknown	938	160	China
SAMN08295858	26	PRJNA428542	PH	3	117	2	China
SAMN08295859	26	PRJNA428542	PH	5	499	83	China
SAMN08295860	26	PRJNA428542	PH	16	250	49	China
SAMN08295861	26	PRJNA428542	PH	Unknown	938	160	China
SAMN08295862	26	PRJNA428542	PH	3	117	2	China
SAMN08295863	26	PRJNA428542	PH	3	117	2	China
SAMN08295864	26	PRJNA428542	PH	3	117	2	China
SAMN08295865	26	PRJNA428542	PH	16	250	49	China
SAMN08295866	26	PRJNA428542	PH	16	Unknown	Unknown	China
SAMN08295867	26	PRJNA428542	PH	3	117	2	China
SAMN08295868	26	PRJNA428542	PH	16	925	152	China
SAMN08295869	26	PRJNA428542	PH	16	925	152	China
SAMN08295870	26	PRJNA428542	PH	25	917	147	China
SAMN08295871	26	PRJNA428542	PH	25	917	147	China
SAMN08295872	26	PRJNA428542	PH	16	925	152	China
SAMN08295873	26	PRJNA428542	PH	16	925	152	China
SAMN08295874	26	PRJNA428542	PH	Unknown	Unknown	Unknown	China

SAMN08295875	26	PRJNA428542	PH	15	Unknown	Unknown	China
SAMN08295876	26	PRJNA428542	PH	12	Unknown	Unknown	China
SAMN08295877	26	PRJNA428542	PH	16	477	80	China
SAMN08295879	26	PRJNA428542	PH	Unknown	Unknown	Unknown	China
SAMN08295880	26	PRJNA428542	PH	Unknown	Unknown	Unknown	China
SAMN08295881	26	PRJNA428542	PH	Unknown	Unknown	Unknown	China
SAMN08295882	26	PRJNA428542	PH	Unknown	Unknown	Unknown	China
SAMN08295883	26	PRJNA428542	PH	Unknown	Unknown	Unknown	China
SAMN08295885	26	PRJNA428542	PH	15	940	162	China
SAMN08295886	26	PRJNA428542	PH	Unknown	Unknown	Unknown	China
SAMN08295887	26	PRJNA428542	PH	Unknown	933	158	China
SAMN08295888	26	PRJNA428542	PH	Unknown	Unknown	Unknown	China
SAMN08295889	26	PRJNA428542	PH	Unknown	933	158	China
SAMN08295890	26	PRJNA428542	PH	Unknown	933	158	China
SAMN08295891	26	PRJNA428542	PH	16	188	21	China
SAMN08295892	26	PRJNA428542	PH	16	Unknown	Unknown	China
SAMN08295893	26	PRJNA428542	PH	15	Unknown	Unknown	China
SAMN08295894	26	PRJNA428542	PH	Unknown	Unknown	Unknown	China
SAMN08295895	26	PRJNA428542	PH	Unknown	Unknown	Unknown	China
SAMN08295896	26	PRJNA428542	PH	Unknown	Unknown	Unknown	China
SAMN08295897	26	PRJNA428542	PH	16	188	21	China
SAMN08295898	26	PRJNA428542	PH	5	Unknown	Unknown	China
SAMN08295899	26	PRJNA428542	PH	5	Unknown	Unknown	China
SAMN08295900	26	PRJNA428542	PH	Multiple	Unknown	Unknown	China
SAMN08295901	26	PRJNA428542	PH	28	923	19	China
SAMN08295902	26	PRJNA428542	PH	28	923	19	China
SAMN08295903	26	PRJNA428542	PH	1	11	1	China
SAMN08295904	26	PRJNA428542	PH	Unknown	Unknown	Unknown	China
SAMN08295905	26	PRJNA428542	PH	1	11	1	China
SAMN08295906	26	PRJNA428542	PH	31	Unknown	Unknown	China
SAMN08295907	26	PRJNA428542	PH	15	937	159	China
SAMN08295908	26	PRJNA428542	PH	15	937	159	China
SAMN08295909	26	PRJNA428542	PH	8	198	4	China
SAMN08295910	26	PRJNA428542	PH	8	198	4	China
SAMN08295911	26	PRJNA428542	PH	8	198	4	China
SAMN08295912	26	PRJNA428542	PH	8	198	4	China
SAMN08295913	26	PRJNA428542	PH	8	198	4	China
SAMN08295914	26	PRJNA428542	PH	8	198	4	China
SAMN08295915	26	PRJNA428542	PH	5	Unknown	Unknown	China
SAMN08295916	26	PRJNA428542	PH	8	198	4	China
SAMN08295917	26	PRJNA428542	PH	8	198	4	China
SAMN08295918	26	PRJNA428542	PH	15	Unknown	Unknown	China
SAMN08295919	26	PRJNA428542	PH	15	Unknown	Unknown	China
SAMN08295920	26	PRJNA428542	PH	31	Unknown	Unknown	China
SAMN08295921	26	PRJNA428542	PH	31	Unknown	Unknown	China
SAMN08295922	26	PRJNA428542	PH	15	937	159	China
SAMN08295923	26	PRJNA428542	PH	15	937	159	China
SAMN08295924	26	PRJNA428542	PH	31	Unknown	Unknown	China
SAMN08295925	26	PRJNA428542	PH	31	Unknown	Unknown	China
SAMN08295926	26	PRJNA428542	PH	4	94	0	China
SAMN08295927	26	PRJNA428542	PH	4	94	0	China
SAMN08295928	26	PRJNA428542	PH	29	Unknown	Unknown	China
SAMN08295929	26	PRJNA428542	PH	29	Unknown	Unknown	China
SAMN08295930	26	PRJNA428542	PH	16	188	21	China
SAMN08295931	26	PRJNA428542	PH	Unknown	Unknown	Unknown	China
SAMN08295932	26	PRJNA428542	PH	29	Unknown	Unknown	China
SAMN08295933	26	PRJNA428542	PH	16	188	21	China
SAMN08295934	26	PRJNA428542	PH	16	188	21	China
SAMN08295935	26	PRJNA428542	PH	29	Unknown	Unknown	China
SAMN08295936	26	PRJNA428542	PH	Unknown	Unknown	Unknown	China
SAMN08295937	26	PRJNA428542	PH	29	Unknown	Unknown	China
SAMN08295938	26	PRJNA428542	PH	Unknown	Unknown	Unknown	China
SAMN08295939	26	PRJNA428542	PH	16	188	21	China
SAMN08295940	26	PRJNA428542	PH	2	1	1	China
SAMN08295941	26	PRJNA428542	PH	31	926	153	China
SAMN08295942	26	PRJNA428542	PH	4	94	0	China
SAMN08295943	26	PRJNA428542	PH	16	188	21	China
SAMN08295944	26	PRJNA428542	PH	29	Unknown	Unknown	China
SAMN08295945	26	PRJNA428542	PH	29	Unknown	Unknown	China
SAMN08295946	26	PRJNA428542	PH	29	Unknown	Unknown	China
SAMN08295947	26	PRJNA428542	PH	29	Unknown	Unknown	China
SAMN08295948	26	PRJNA428542	PH	31	926	153	China
SAMN08295949	26	PRJNA428542	PH	31	Unknown	Unknown	China
SAMN08295950	26	PRJNA428542	PH	31	Unknown	Unknown	China
SAMN08295951	26	PRJNA428542	PH	29	Unknown	Unknown	China
SAMN08295952	26	PRJNA428542	PH	Unknown	931	156	China
SAMN08295953	26	PRJNA428542	PH	Unknown	930	155	China
SAMN08295954	26	PRJNA428542	PH	Unknown	930	155	China
SAMN08295955	26	PRJNA428542	PH	Unknown	Unknown	Unknown	China
SAMN08295956	26	PRJNA428542	PH	Unknown	931	156	China
SAMN08295957	26	PRJNA428542	PH	Unknown	Unknown	Unknown	China
SAMN08295958	26	PRJNA428542	PH	Unknown	930	155	China
SAMN08295959	26	PRJNA428542	PH	Unknown	Unknown	Unknown	China
SAMN08295960	26	PRJNA428542	PH	Unknown	Unknown	Unknown	China
SAMN08295961	26	PRJNA428542	PH	Unknown	930	155	China
SAMN08295962	26	PRJNA428542	PH	Unknown	Unknown	Unknown	China
SAMN08295963	26	PRJNA428542	PH	Unknown	Unknown	Unknown	China
SAMN08295964	26	PRJNA428542	PH	Unknown	Unknown	Unknown	China
SAMN08295965	26	PRJNA428542	PH	15	Unknown	Unknown	China
SAMN08295966	26	PRJNA428542	PH	Unknown	Unknown	Unknown	China
SAMN08295967	26	PRJNA428542	PH	Unknown	Unknown	Unknown	China
SAMN08295968	26	PRJNA428542	PH	16	Unknown	Unknown	China
SAMN08295969	26	PRJNA428542	PH	16	Unknown	Unknown	China
SAMN08295970	26	PRJNA428542	PH	16	Unknown	Unknown	China
SAMN08295971	26	PRJNA428542	PH	Unknown	Unknown	Unknown	China
SAMN08295973	26	PRJNA428542	PH	16	477	80	China
SAMN08295974	26	PRJNA428542	PH	Unknown	Unknown	Unknown	China
SAMN08295975	26	PRJNA428542	PH	16	477	80	China
SAMN08295976	26	PRJNA428542	PH	16	477	80	China
SAMN08295977	26	PRJNA428542	PH	11	Unknown	Unknown	China
SAMN08295978	26	PRJNA428542	PH	16	477	80	China
SAMN08295979	26	PRJNA428542	PH	16	477	80	China
SAMN08295980	26	PRJNA428542	PH	16	477	80	China
SAMN08295981	26	PRJNA428542	PH	15	Unknown	Unknown	China
SAMN08295982	26	PRJNA428542	PH	Multiple	Unknown	Unknown	China
SAMN08295983	26	PRJNA428542	PH	16	925	152	China
SAMN08295984	26	PRJNA428542	PH	Unknown	Unknown	Unknown	China
SAMN08295985	26	PRJNA428542	PH	15	Unknown	Unknown	China
SAMN08295986	26	PRJNA428542	PH	16	477	80	China
SAMN08295987	26	PRJNA428542	PH	16	477	80	China
SAMN08295988	26	PRJNA428542	PH	16	477	80	China
SAMN08295989	26	PRJNA428542	PH	16	925	152	China
SAMN08295990	26	PRJNA428542	PH	11	Unknown	Unknown	China
SAMN08295991	26	PRJNA428542	PH	Unknown	423	72	China
SAMN08295992	26	PRJNA428542	PH	2	7	1	China
SAMN08295993	26	PRJNA428542	PH	Unknown	423	72	China
SAMN08295994	26	PRJNA428542	PH	4	Unknown	Unknown	China
SAMN08295995	26	PRJNA428542	PH	4	Unknown	Unknown	China
SAMN08295996	26	PRJNA428542	PH	5	499	83	China

SAMN08295997	26	PRJNA428542	PH	21	Unknown	Unknown	China
SAMN08295998	26	PRJNA428542	PH	12	939	161	China
SAMN08295999	26	PRJNA428542	PH	12	939	161	China
SAMN08296000	26	PRJNA428542	PH	12	939	161	China
SAMN08296001	26	PRJNA428542	PH	Unknown	932	157	China
SAMN08296003	26	PRJNA428542	PH	21	Unknown	Unknown	China
SAMN08296004	26	PRJNA428542	PH	Multiple	918	19	China
SAMN08296005	26	PRJNA428542	PH	16	188	21	China
SAMN08296006	26	PRJNA428542	PH	31	921	150	China
SAMN08296007	26	PRJNA428542	PH	31	921	150	China
SAMN08296009	26	PRJNA428542	PH	16	188	21	China
SAMN08296010	26	PRJNA428542	PH	16	188	21	China
SAMN08296011	26	PRJNA428542	PH	16	188	21	China
SAMN08296013	26	PRJNA428542	PH	31	926	153	China
SAMN08296014	26	PRJNA428542	PH	16	927	20	China
SAMN08296015	26	PRJNA428542	PH	28	Unknown	Unknown	China
SAMN08296016	26	PRJNA428542	PH	28	919	148	China
SAMN08296017	26	PRJNA428542	PH	16	928	20	China
SAMN08296018	26	PRJNA428542	PH	28	919	148	China
SAMN08296019	26	PRJNA428542	PH	16	928	20	China
SAMN08296020	26	PRJNA428542	PH	30	Unknown	Unknown	China
SAMN08296021	26	PRJNA428542	PH	28	919	148	China
SAMN08296022	26	PRJNA428542	PH	29	Unknown	Unknown	China
SAMN08296023	26	PRJNA428542	PH	15	Unknown	Unknown	China
SAMN08296024	26	PRJNA428542	PH	29	Unknown	Unknown	China
SAMN08296025	26	PRJNA428542	PH	21	Unknown	Unknown	China
SAMN08296026	26	PRJNA428542	PH	Unknown	Unknown	Unknown	China
SAMN08296027	26	PRJNA428542	PH	Unknown	Unknown	Unknown	China
SAMN08296029	26	PRJNA428542	PH	Unknown	Unknown	Unknown	China
SAMN08296030	26	PRJNA428542	PH	31	Unknown	Unknown	China
SAMN08296031	26	PRJNA428542	PH	16	Unknown	Unknown	China
SAMN08296034	26	PRJNA428542	PH	Unknown	Unknown	Unknown	China
SAMN08296035	26	PRJNA428542	PH	Unknown	Unknown	Unknown	China
SAMN08296037	26	PRJNA428542	PH	15	Unknown	Unknown	China
SAMN08296038	26	PRJNA428542	PH	2	7	1	China
SAMN08296039	26	PRJNA428542	PH	2	7	1	China
SAMN08296040	26	PRJNA428542	PH	16	934	21	China
SAMN08296041	26	PRJNA428542	PH	5	499	83	China
SAMN08296042	26	PRJNA428542	PH	9	Unknown	Unknown	China
SAMN08296045	26	PRJNA428542	PH	Unknown	Unknown	Unknown	China
SAMN08296047	26	PRJNA428542	PH	Unknown	929	154	China
SAMN08296048	26	PRJNA428542	PH	Unknown	Unknown	Unknown	China
SAMN08296050	26	PRJNA428542	PH	Unknown	Unknown	Unknown	China
SAMN08296051	26	PRJNA428542	PH	Unknown	Unknown	Unknown	China
SAMN08296052	26	PRJNA428542	PH	Unknown	Unknown	Unknown	China
SAMN08296053	26	PRJNA428542	PH	Unknown	Unknown	Unknown	China
SAMN08296054	26	PRJNA428542	PH	Unknown	Unknown	Unknown	China
SAMN08296057	26	PRJNA428542	PH	15	Unknown	Unknown	China
SAMN08296058	26	PRJNA428542	PH	Unknown	922	151	China
SAMN09080446	27	PRJNA464270	PD	5	498	82	China
SAMN10651136	27	PRJNA511911	PD	2	7	1	China
SAMN09459599	28	PRJNA476804	PD	Unknown	27	2	Australia
SAMN09459600	28	PRJNA476804	PD	8	Unknown	Unknown	Australia
SAMN09459601	28	PRJNA476804	PD	Unknown	27	2	Australia
SAMN09459602	28	PRJNA476804	PD	10	1056	193	Australia
SAMN09459603	28	PRJNA476804	PD	8	87	3	Australia
SAMN09459604	28	PRJNA476804	PD	2	25	2	Australia
SAMN09459605	28	PRJNA476804	PD	Unknown	27	2	Australia
SAMN09459606	28	PRJNA476804	PD	2	25	2	Australia
SAMN09459607	28	PRJNA476804	PD	3	Unknown	Unknown	Australia
SAMN09459608	28	PRJNA476804	PD	2	28	2	Australia
SAMN09459609	28	PRJNA476804	PD	11	1032	190	Australia
SAMN09459610	28	PRJNA476804	PD	3	27	2	Australia
SAMN09459612	28	PRJNA476804	PD	Unknown	28	2	Australia
SAMN09459613	28	PRJNA476804	PD	2	28	2	Australia
SAMN09459614	28	PRJNA476804	PD	Unknown	856	2	Australia
SAMN09459615	28	PRJNA476804	PD	2	28	2	Australia
SAMN09459616	28	PRJNA476804	PD	31	Unknown	Unknown	Australia
SAMN09459617	28	PRJNA476804	PD	1	Unknown	Unknown	Australia
SAMN09459618	28	PRJNA476804	PD	Unknown	1031	189	Australia
SAMN09459620	28	PRJNA476804	PD	12	1032	190	Australia
SAMN09459621	28	PRJNA476804	PD	2	25	2	Australia
SAMN09459622	28	PRJNA476804	PD	2	1	1	Australia
SAMN09459623	28	PRJNA476804	PD	2	1	1	Australia
SAMN09459624	28	PRJNA476804	PD	4	483	0	Australia
SAMN09459625	28	PRJNA476804	PD	7	Unknown	Unknown	Australia
SAMN09459626	28	PRJNA476804	PD	3	27	2	Australia
SAMN09459627	28	PRJNA476804	PD	19	76	34	Australia
SAMN09459628	28	PRJNA476804	PD	19	76	34	Australia
SAMN09459629	28	PRJNA476804	PD	21	Unknown	Unknown	Australia
SAMN09459630	28	PRJNA476804	PD	Unknown	27	2	Australia
SAMN09459633	28	PRJNA476804	PD	Unknown	27	2	Australia
SAMN09459634	28	PRJNA476804	PD	8	87	3	Australia
SAMN09459635	28	PRJNA476804	PD	3	27	2	Australia
SAMN09459636	28	PRJNA476804	PD	2	28	2	Australia
SAMN09459637	28	PRJNA476804	PD	2	28	2	Australia
SAMN09459638	28	PRJNA476804	PD	2	28	2	Australia
SAMN09459639	28	PRJNA476804	PD	3	27	2	Australia
SAMN09459640	28	PRJNA476804	PD	Unknown	27	2	Australia
SAMN09459641	28	PRJNA476804	PD	3	27	2	Australia
SAMN09459643	28	PRJNA476804	PD	4	Unknown	Unknown	Australia
SAMN09459644	28	PRJNA476804	PD	4	Unknown	Unknown	Australia
SAMN09459645	28	PRJNA476804	PD	2	25	2	Australia
SAMN09459646	28	PRJNA476804	PD	3	27	2	Australia
SAMN09459647	28	PRJNA476804	PD	2	25	2	Australia
SAMN09459648	28	PRJNA476804	PD	2	25	2	Australia
SAMN09459649	28	PRJNA476804	PD	2	28	2	Australia
SAMN09459650	28	PRJNA476804	PD	2	Unknown	Unknown	Australia
SAMN09459651	28	PRJNA476804	PD	3	27	2	Australia
SAMN09459652	28	PRJNA476804	PD	19	Unknown	Unknown	Australia
SAMN09459654	28	PRJNA476804	PD	18	Unknown	Unknown	Australia
SAMN09459655	28	PRJNA476804	PD	7	Unknown	Unknown	Australia
SAMN09459659	28	PRJNA476804	PD	Unknown	27	2	Australia
SAMN09459660	28	PRJNA476804	PD	2	1055	192	Australia
SAMN09459661	28	PRJNA476804	PD	2	28	2	Australia
SAMN09459662	28	PRJNA476804	PD	2	25	2	Australia
SAMN09459663	28	PRJNA476804	PD	2	25	2	Australia
SAMN09459664	28	PRJNA476804	PD	2	25	2	Australia
SAMN09459665	28	PRJNA476804	PD	2	25	2	Australia
SAMN09459666	28	PRJNA476804	PD	2	25	2	Australia
SAMN09459667	28	PRJNA476804	PD	2	25	2	Australia
SAMN09459668	28	PRJNA476804	PD	23	Unknown	Unknown	Australia
SAMN09459669	28	PRJNA476804	PD	19	76	34	Australia
SAMN09459670	28	PRJNA476804	PD	16	Unknown	Unknown	Australia
SAMN09459671	28	PRJNA476804	PD	3	27	2	Australia
SAMN09459673	28	PRJNA476804	PD	3	117	2	Australia
SAMN09459674	28	PRJNA476804	PD	5	483	0	Australia
SAMN09459676	28	PRJNA476804	PD	3	117	2	Australia
SAMN09459677	28	PRJNA476804	PD	3	27	2	Australia

SAMN09459678	28	PRJNA476804	PD	2	25	2	Australia
SAMN09459679	28	PRJNA476804	PD	31	Unknown	Unknown	Australia
SAMN09459680	28	PRJNA476804	PD	9	Unknown	Unknown	Australia
SAMN09459681	28	PRJNA476804	PD	Unknown	856	2	Australia
SAMN09459683	28	PRJNA476804	PD	21	1034	24	Australia
SAMN09459684	28	PRJNA476804	PD	16	Unknown	Unknown	Australia
SAMN09459685	28	PRJNA476804	PD	21	Unknown	Unknown	Australia
SAMN09459686	28	PRJNA476804	PD	3	27	2	Australia
SAMN09459687	28	PRJNA476804	PD	21	Unknown	Unknown	Australia
SAMN09459688	28	PRJNA476804	PD	2	28	2	Australia
SAMN09459690	28	PRJNA476804	PD	3	Unknown	Unknown	Australia
SAMN09459691	28	PRJNA476804	PD	16	Unknown	Unknown	Australia
SAMN09459692	28	PRJNA476804	PD	7	Unknown	Unknown	Australia
SAMN09459693	28	PRJNA476804	PD	3	Unknown	Unknown	Australia
SAMN09459694	28	PRJNA476804	PD	7	Unknown	Unknown	Australia
SAMN09459695	28	PRJNA476804	PD	12	1032	190	Australia
SAMN09459696	28	PRJNA476804	PD	16	Unknown	Unknown	Australia
SAMN09459697	28	PRJNA476804	PD	16	Unknown	Unknown	Australia
SAMN09459698	28	PRJNA476804	PD	8	87	3	Australia
SAMN09459699	28	PRJNA476804	PD	2	25	2	Australia
SAMN09459700	28	PRJNA476804	PD	2	25	2	Australia
SAMN09459701	28	PRJNA476804	PD	2	25	2	Australia
SAMN09459702	28	PRJNA476804	PD	2	25	2	Australia
SAMN09459703	28	PRJNA476804	PD	2	25	2	Australia
SAMN09459704	28	PRJNA476804	PD	Unknown	25	2	Australia
SAMN09459705	28	PRJNA476804	PD	Unknown	28	2	Australia
SAMN09459708	28	PRJNA476804	PD	5	483	0	Australia
SAMN09459709	28	PRJNA476804	PD	2	1	1	Australia
SAMN09459710	28	PRJNA476804	PD	4	94	0	Australia
SAMN09459711	28	PRJNA476804	PD	2	1	1	Australia
SAMN09459712	28	PRJNA476804	PD	5	483	0	Australia
SAMN09459713	28	PRJNA476804	PD	2	1	1	Australia
SAMN09459714	28	PRJNA476804	PD	2	1	1	Australia
SAMN09459715	28	PRJNA476804	PD	2	1	1	Australia
SAMN09459717	28	PRJNA476804	PD	16	Unknown	Unknown	Australia
SAMN09459718	28	PRJNA476804	PD	2	1	1	Australia
SAMN09459719	28	PRJNA476804	PD	3	27	2	Australia
SAMN09459720	28	PRJNA476804	PD	3	117	2	Australia
SAMN09459721	28	PRJNA476804	PD	Unknown	Unknown	Unknown	Australia
SAMN09459722	28	PRJNA476804	PD	3	Unknown	Unknown	Australia
SAMN09459723	28	PRJNA476804	PD	8	Unknown	Unknown	Australia
SAMN09459724	28	PRJNA476804	PD	3	27	2	Australia
SAMN09459726	28	PRJNA476804	PD	2	25	2	Australia
SAMN09459727	28	PRJNA476804	PD	12	Unknown	Unknown	Australia
SAMN09459728	28	PRJNA476804	PD	2	1	1	Australia
SAMN09459729	28	PRJNA476804	PD	Unknown	Unknown	Unknown	Australia
SAMN09459730	28	PRJNA476804	PD	23	Unknown	Unknown	Australia
SAMN09459731	28	PRJNA476804	PD	19	76	34	Australia
SAMN09459732	28	PRJNA476804	PD	16	Unknown	Unknown	Australia
SAMN09459733	28	PRJNA476804	PD	16	Unknown	Unknown	Australia
SAMN09459735	28	PRJNA476804	PD	Unknown	Unknown	Unknown	Australia
SAMN09459736	28	PRJNA476804	PD	8	87	3	Australia
SAMN09459737	28	PRJNA476804	PD	15	1031	189	Australia
SAMN09459738	28	PRJNA476804	PD	Unknown	117	2	Australia
SAMN09459739	28	PRJNA476804	PD	31	Unknown	Unknown	Australia
SAMN09459740	28	PRJNA476804	PD	1	1	1	Australia
SAMN09459741	28	PRJNA476804	PD	8	Unknown	Unknown	Australia
SAMN09459742	28	PRJNA476804	PD	29	Unknown	Unknown	Australia
SAMN09459743	28	PRJNA476804	PD	5	1033	191	Australia
SAMN09459744	28	PRJNA476804	PD	3	27	2	Australia
SAMN09459745	28	PRJNA476804	PD	Unknown	27	2	Australia
SAMN09459746	28	PRJNA476804	PD	3	27	2	Australia
SAMN10440286	29	PRJNA505967	PH	8	Unknown	Unknown	Germany
SAMN10440287	29	PRJNA505967	PD	Unknown	1098	195	Germany
SAMN10464066	30	PRJNA506583	PD	9	1105	196	Switzerland
SAMN10464067	30	PRJNA506583	PD	1	13	9	Switzerland
SAMN10464068	30	PRJNA506583	PD	6	1104	25	Switzerland
SAMN10464070	30	PRJNA506583	PD	2	1133	2	Switzerland
SAMN10464071	30	PRJNA506583	PD	6	1104	25	Switzerland
SAMN10464072	30	PRJNA506583	PD	2	28	2	Switzerland
SAMN10464073	30	PRJNA506583	PD	Unknown	1108	197	Switzerland
SAMN10464074	30	PRJNA506583	PD	9	1105	196	Switzerland
SAMN10464075	30	PRJNA506583	PD	1	13	9	Switzerland
SAMN10464076	30	PRJNA506583	PD	1	13	9	Switzerland
SAMN10464077	30	PRJNA506583	PD	Unknown	1238	198	Switzerland
SAMN10464078	30	PRJNA506583	PD	6	1104	25	Switzerland
SAMN10464079	30	PRJNA506583	PD	2	28	2	Switzerland
SAMN10464080	30	PRJNA506583	PD	6	1104	25	Switzerland
SAMN11431410	31	PRJNA532985	PH	Unknown	1090	194	China
SAMN11489679	32	PRJNA534412	PD	2	7	1	China
SAMD00029776	33	PRJDB3844	PH	2	28	2	Japan
SAMD00029777	33	PRJDB3844	PH	2	28	2	Japan
SAMD00029778	33	PRJDB3844	PH	2	28	2	Japan
SAMD00029779	33	PRJDB3844	PH	2	28	2	Japan
SAMD00029780	33	PRJDB3844	PH	2	28	2	Japan
SAMD00029781	33	PRJDB3844	PH	2	28	2	Japan
SAMD00029782	33	PRJDB3844	PH	2	1	1	Japan
SAMD00029783	33	PRJDB3844	PH	2	1	1	Japan
SAMD00029784	33	PRJDB3844	PH	Unknown	1	1	Japan
SAMD00029785	33	PRJDB3844	PH	2	1	1	Japan
SAMD00029786	33	PRJDB3844	PH	2	1	1	Japan
SAMD00029787	33	PRJDB3844	PH	2	1	1	Japan
SAMD00029788	33	PRJDB3844	PH	2	28	2	Japan
SAMD00029789	33	PRJDB3844	PH	2	28	2	Japan
SAMD00029790	33	PRJDB3844	PH	2	1	1	Japan
SAMD00029791	33	PRJDB3844	PH	2	1	1	Japan
SAMD00029792	33	PRJDB3844	PH	Unknown	Unknown	Unknown	Japan
SAMD00029793	33	PRJDB3844	PH	2	28	2	Japan
SAMD00029794	33	PRJDB3844	PH	2	28	2	Japan
SAMD00029795	33	PRJDB3844	PH	2	28	2	Japan
SAMD00029796	33	PRJDB3844	PH	2	28	2	Japan
SAMD00029797	33	PRJDB3844	PH	2	28	2	Japan
SAMD00029798	33	PRJDB3844	PH	2	28	2	Japan
SAMD00029799	33	PRJDB3844	PH	2	28	2	Japan
SAMD00029800	33	PRJDB3844	PH	2	28	2	Japan
SAMD00029801	33	PRJDB3844	PH	2	28	2	Japan
SAMD00029802	33	PRJDB3844	PH	2	28	2	Japan
SAMD00029803	33	PRJDB3844	PH	2	28	2	Japan
SAMD00029804	33	PRJDB3844	PH	2	28	2	Japan
SAMD00029805	33	PRJDB3844	PH	2	28	2	Japan
SAMD00029806	33	PRJDB3844	PH	2	28	2	Japan
SAMD00029807	33	PRJDB3844	PH	2	28	2	Japan
SAMD00029808	33	PRJDB3844	PH	2	28	2	Japan
SAMD00029809	33	PRJDB3844	PH	2	28	2	Japan
SAMD00029810	33	PRJDB3844	PH	2	28	2	Japan
SAMD00029811	33	PRJDB3844	PH	2	28	2	Japan
SAMD00029812	33	PRJDB3844	PH	2	1	1	Japan
SAMD00029813	33	PRJDB3844	PH	2	1	1	Japan
SAMD00029814	33	PRJDB3844	PH	Unknown	28	2	Japan

SAMD00029815	33	PRJDB3844	PH	2	28	2	Japan
SAMD00029816	33	PRJDB3844	PH	2	28	2	Japan
SAMD00029817	33	PRJDB3844	PH	2	28	2	Japan
SAMD00029818	33	PRJDB3844	PH	2	28	2	Japan
SAMD00029819	33	PRJDB3844	PH	2	28	2	Japan
SAMD00029820	33	PRJDB3844	PH	2	28	2	Japan
SAMD00029821	33	PRJDB3844	PH	2	28	2	Japan
SAMD00029822	33	PRJDB3844	PH	2	1	1	Japan
SAMD00029823	33	PRJDB3844	PH	Unknown	1	1	Japan
SAMD00029824	33	PRJDB3844	PH	2	28	2	Japan
SAMD00029825	33	PRJDB3844	PH	2	28	2	Japan
SAMD00029826	33	PRJDB3844	PH	Unknown	28	2	Japan
SAMD00029827	33	PRJDB3844	PH	Unknown	28	2	Japan
SAMD00066479	34	PRJDB5283	H	5	752	0	Japan
SAMD00113017	35	PRJDB6789	H	Unknown	28	2	Japan
SAMEA103980380	36	PRJEB20417	H	2	1	1	Netherlands
SAMEA3595173	37	PRJEB11219	PD	1	1	1	Netherlands
SAMEA3595174	37	PRJEB11219	PD	1	1	1	Netherlands
SAMEA3595175	37	PRJEB11219	PD	1	13	9	Netherlands
SAMEA3595176	37	PRJEB11219	PD	9	136	4	Netherlands
SAMEA3595177	37	PRJEB11219	H	2	20	6	Netherlands
SAMEA3595178	37	PRJEB11219	H	2	1	1	Netherlands
SAMEA3595179	37	PRJEB11219	H	2	1	1	Netherlands
SAMEA3595180	37	PRJEB11219	H	2	134	1	Netherlands
SAMEA3595181	37	PRJEB11219	H	2	1	1	Netherlands
SAMEA3595182	37	PRJEB11219	H	2	20	6	Netherlands
SAMEA3595183	37	PRJEB11219	H	2	20	6	Netherlands
SAMEA3595184	37	PRJEB11219	H	2	1	1	Netherlands
SAMEA3595185	37	PRJEB11219	H	2	20	6	Netherlands
SAMEA3595186	37	PRJEB11219	H	2	1	1	Netherlands
SAMEA3595187	37	PRJEB11219	H	2	20	6	Netherlands
SAMEA3595188	37	PRJEB11219	H	2	20	6	Netherlands
SAMEA3595189	37	PRJEB11219	H	2	1	1	Netherlands
SAMEA3595190	37	PRJEB11219	H	2	1	1	Netherlands
SAMEA3595191	37	PRJEB11219	H	2	1	1	Netherlands
SAMEA3595192	37	PRJEB11219	H	2	20	6	Netherlands
SAMEA3595193	37	PRJEB11219	H	2	1	1	Netherlands
SAMEA3595194	37	PRJEB11219	H	2	1	1	Netherlands
SAMEA3595195	37	PRJEB11219	H	2	20	6	Netherlands
SAMEA3595196	37	PRJEB11219	H	2	20	6	Netherlands
SAMEA3595197	37	PRJEB11219	H	2	20	6	Netherlands
SAMEA3595198	37	PRJEB11219	H	2	146	1	Netherlands
SAMEA3595199	37	PRJEB11219	H	2	1	1	Netherlands
SAMEA3595200	37	PRJEB11219	H	2	1	1	Netherlands
SAMEA3595201	37	PRJEB11219	PD	9	220	46	Netherlands
SAMEA3595202	37	PRJEB11219	PD	1	13	9	Netherlands
SAMEA3595203	37	PRJEB11219	PD	9	16	4	Netherlands
SAMEA3595204	37	PRJEB11219	PD	9	16	4	Netherlands
SAMEA3595205	37	PRJEB11219	PD	8	87	3	Netherlands
SAMEA3595206	37	PRJEB11219	PD	7	29	2	Netherlands
SAMEA3595207	37	PRJEB11219	PD	1	1	1	Netherlands
SAMEA3595208	37	PRJEB11219	PD	1	132	1	Netherlands
SAMEA3595209	37	PRJEB11219	PD	2	20	6	Netherlands
SAMEA3595210	37	PRJEB11219	PD	9	16	4	Netherlands
SAMEA3595211	37	PRJEB11219	PD	9	16	4	Netherlands
SAMEA3595212	37	PRJEB11219	PD	9	16	4	Netherlands
SAMEA3595213	37	PRJEB11219	PD	9	16	4	Netherlands
SAMEA3595214	37	PRJEB11219	PD	2	28	2	Netherlands
SAMEA3595215	37	PRJEB11219	PD	2	1	1	Netherlands
SAMEA3595216	37	PRJEB11219	PD	9	16	4	Netherlands
SAMEA3595217	37	PRJEB11219	PD	2	1	1	Netherlands
SAMEA3595218	37	PRJEB11219	PD	31	183	43	Netherlands
SAMEA3595219	37	PRJEB11219	PD	9	16	4	Netherlands
SAMEA3595220	37	PRJEB11219	PD	9	16	4	Netherlands
SAMEA3595221	37	PRJEB11219	PD	1	1	1	Netherlands
SAMEA3595222	37	PRJEB11219	PD	2	1	1	Netherlands
SAMEA3595223	37	PRJEB11219	PD	16	218	45	Netherlands
SAMEA3595224	37	PRJEB11219	PD	9	16	4	Netherlands
SAMEA3595225	37	PRJEB11219	PD	7	29	2	Netherlands
SAMEA3595226	37	PRJEB11219	PD	1	13	9	Netherlands
SAMEA3595227	37	PRJEB11219	PD	9	16	4	Netherlands
SAMEA3595228	37	PRJEB11219	PD	1	1	1	Netherlands
SAMEA3595229	37	PRJEB11219	PD	2	1	1	Netherlands
SAMEA3595230	37	PRJEB11219	PD	3	15	3	Netherlands
SAMEA3595231	37	PRJEB11219	PD	9	136	4	Netherlands
SAMEA3595232	37	PRJEB11219	PD	9	16	4	Netherlands
SAMEA3595233	37	PRJEB11219	PD	1	13	9	Netherlands
SAMEA3595234	37	PRJEB11219	PD	9	16	4	Netherlands
SAMEA3595235	37	PRJEB11219	PD	4	17	6	Netherlands
SAMEA3595236	37	PRJEB11219	PD	7	854	2	Netherlands
SAMEA3595237	37	PRJEB11219	PD	3	15	3	Netherlands
SAMEA3595238	37	PRJEB11219	PD	2	1	1	Netherlands
SAMEA3595239	37	PRJEB11219	PD	7	29	2	Netherlands
SAMEA3595240	37	PRJEB11219	PD	7	29	2	Netherlands
SAMEA3595241	37	PRJEB11219	PD	9	155	4	Netherlands
SAMEA3595242	37	PRJEB11219	PD	4	17	6	Netherlands
SAMEA3595243	37	PRJEB11219	PD	9	16	4	Netherlands
SAMEA3595244	37	PRJEB11219	PD	2	1	1	Netherlands
SAMEA3595245	37	PRJEB11219	PD	9	151	4	Netherlands
SAMEA3595246	37	PRJEB11219	PD	9	136	4	Netherlands
SAMEA3595247	37	PRJEB11219	PD	9	16	4	Netherlands
SAMEA3595248	37	PRJEB11219	PD	9	16	4	Netherlands
SAMEA3595249	37	PRJEB11219	PD	9	136	4	Netherlands
SAMEA3595250	37	PRJEB11219	PD	9	16	4	Netherlands
SAMEA3595251	37	PRJEB11219	PD	2	1	1	Netherlands
SAMEA3595252	37	PRJEB11219	PD	7	29	2	Netherlands
SAMEA3595253	37	PRJEB11219	PD	2	20	6	Netherlands
SAMEA3595254	37	PRJEB11219	PD	4	17	6	Netherlands
SAMEA3595255	37	PRJEB11219	PD	9	16	4	Netherlands
SAMEA3595256	37	PRJEB11219	PD	2	1	1	Netherlands
SAMEA3595257	37	PRJEB11219	PD	17	184	44	Netherlands
SAMEA3595258	37	PRJEB11219	PD	9	16	4	Netherlands
SAMEA3595259	37	PRJEB11219	PD	2	1	1	Netherlands
SAMEA3595260	37	PRJEB11219	PD	1	149	9	Netherlands
SAMEA3595261	37	PRJEB11219	PD	9	136	4	Netherlands
SAMEA3595262	37	PRJEB11219	PD	8	198	4	Netherlands
SAMEA3595263	37	PRJEB11219	PD	1	1	1	Netherlands
SAMEA3595264	37	PRJEB11219	PD	2	1	1	Netherlands
SAMEA3595265	37	PRJEB11219	PD	9	16	4	Netherlands
SAMEA3595266	37	PRJEB11219	PD	2	20	6	Netherlands
SAMEA3595267	37	PRJEB11219	PD	8	153	42	Netherlands
SAMEA3595268	37	PRJEB11219	PD	1	13	9	Netherlands
SAMEA3595269	37	PRJEB11219	PD	2	1	1	Netherlands
SAMEA3595270	37	PRJEB11219	PD	2	1	1	Netherlands
SAMEA4355190	38	PRJEB12952	H	2	956	1	Togo
SAMEA4355191	38	PRJEB12952	H	2	956	1	Togo
SAMEA4355192	38	PRJEB12952	H	2	956	1	Togo
SAMEA4355193	38	PRJEB12952	H	2	956	1	Togo
SAMEA4355194	38	PRJEB12952	H	2	956	1	Togo

SAMEA4355195	38	PRJEB12952	H	2	956	1	Togo
SAMEA4355196	38	PRJEB12952	H	2	956	1	Togo
SAMEA4355197	38	PRJEB12952	H	2	956	1	Togo
SAMEA4355198	38	PRJEB12952	H	2	956	1	Togo
SAMEA4355199	38	PRJEB12952	H	Unknown	956	1	Togo
SAMEA4355200	38	PRJEB12952	H	2	1	1	Togo
SAMEA4355201	38	PRJEB12952	H	2	956	1	Togo
SAMEA4355202	38	PRJEB12952	H	2	1	1	Togo
SAMEA4355203	38	PRJEB12952	H	2	1	1	Togo
SAMEA4355204	38	PRJEB12952	H	2	1	1	Togo
SAMEA5206224	39	PRJEB30600	PH	2	1	1	Netherlands
SAMEA6227224	40	PRJEB35407	PD	2	1	1	Netherlands
SAMEA6227225	40	PRJEB35407	H	2	20	6	Netherlands
SAMEA6227226	40	PRJEB35407	PD	2	20	6	Netherlands
SAMEA6227227	40	PRJEB35407	PD	9	16	4	Netherlands
SAMEA6227228	40	PRJEB35407	PD	9	220	46	Netherlands
SAMN04166386	41	PRJNA298752	H	2	7	1	China
SAMN04166387	41	PRJNA298752	PD	2	7	1	China
SAMN04166388	41	PRJNA298752	H	2	7	1	China
SAMN04166402	41	PRJNA298752	H	2	7	1	China
SAMN04166403	41	PRJNA298752	H	2	7	1	China
SAMN04166404	41	PRJNA298752	H	2	7	1	China
SAMN04166405	41	PRJNA298752	H	2	7	1	China
SAMN04166406	41	PRJNA298752	H	2	7	1	China
SAMN04166407	41	PRJNA298752	H	2	7	1	China
SAMN04166408	41	PRJNA298752	H	2	7	1	China
SAMN04166409	41	PRJNA298752	H	2	7	1	China
SAMN04166410	41	PRJNA298752	H	2	7	1	China
SAMN04166411	41	PRJNA298752	PD	2	7	1	China
SAMN04166412	41	PRJNA298752	H	2	7	1	China
SAMN04166413	41	PRJNA298752	PD	2	7	1	China
SAMN04166414	41	PRJNA298752	H	2	7	1	China
SAMN04166415	41	PRJNA298752	H	2	7	1	China
SAMN04166416	41	PRJNA298752	H	2	7	1	China
SAMN04166417	41	PRJNA298752	H	2	7	1	China
SAMN04166418	41	PRJNA298752	H	2	7	1	China
SAMN04166419	41	PRJNA298752	H	2	7	1	China
SAMN04166420	41	PRJNA298752	H	2	7	1	China
SAMN04166421	41	PRJNA298752	H	2	7	1	China
SAMN04166422	41	PRJNA298752	H	2	7	1	China
SAMN04166423	41	PRJNA298752	H	2	7	1	China
SAMN04166424	41	PRJNA298752	H	2	7	1	China
SAMN04166425	41	PRJNA298752	H	2	7	1	China
SAMN04166426	41	PRJNA298752	H	2	7	1	China
SAMN04166427	41	PRJNA298752	H	2	7	1	China
SAMN04166428	41	PRJNA298752	H	2	7	1	China
SAMN04166429	41	PRJNA298752	H	2	7	1	China
SAMN04166430	41	PRJNA298752	H	2	7	1	China
SAMN04166431	41	PRJNA298752	H	2	7	1	China
SAMN04166432	41	PRJNA298752	H	2	7	1	China
SAMN04166433	41	PRJNA298752	H	2	7	1	China
SAMN04166434	41	PRJNA298752	H	2	7	1	China
SAMN04166435	41	PRJNA298752	H	2	7	1	China
SAMN04166436	41	PRJNA298752	H	2	7	1	China
SAMN04166437	41	PRJNA298752	H	2	7	1	China
SAMN04166438	41	PRJNA298752	H	2	7	1	China
SAMN04166439	41	PRJNA298752	H	2	7	1	China
SAMN04166440	41	PRJNA298752	H	2	7	1	China
SAMN04166441	41	PRJNA298752	H	2	7	1	China
SAMN04166442	41	PRJNA298752	H	2	7	1	China
SAMN04166443	41	PRJNA298752	H	2	7	1	China
SAMN04166444	41	PRJNA298752	H	2	7	1	China
SAMN04166445	41	PRJNA298752	H	2	7	1	China
SAMN04166446	41	PRJNA298752	H	2	7	1	China
SAMN04166447	41	PRJNA298752	H	2	7	1	China
SAMN04166448	41	PRJNA298752	H	2	7	1	China
SAMN04166449	41	PRJNA298752	H	2	7	1	China
SAMN04166450	41	PRJNA298752	H	2	7	1	China
SAMN04166451	41	PRJNA298752	H	2	7	1	China
SAMN04166452	41	PRJNA298752	H	2	7	1	China
SAMN04166453	41	PRJNA298752	H	2	7	1	China
SAMN04166454	41	PRJNA298752	H	2	7	1	China
SAMN04166455	41	PRJNA298752	H	2	7	1	China
SAMN04166456	41	PRJNA298752	PD	2	7	1	China
SAMN04166457	41	PRJNA298752	H	2	7	1	China
SAMN04166458	41	PRJNA298752	H	2	7	1	China
SAMN04166459	41	PRJNA298752	H	2	7	1	China
SAMN04166460	41	PRJNA298752	H	2	7	1	China
SAMN04166461	41	PRJNA298752	H	2	7	1	China
SAMN04166462	41	PRJNA298752	H	2	7	1	China
SAMN04166463	41	PRJNA298752	H	2	7	1	China
SAMN04166464	41	PRJNA298752	H	2	7	1	China
SAMN04166465	41	PRJNA298752	H	2	7	1	China
SAMN04166466	41	PRJNA298752	H	2	7	1	China
SAMN04166467	41	PRJNA298752	H	2	7	1	China
SAMN04166468	41	PRJNA298752	H	2	7	1	China
SAMN04166469	41	PRJNA298752	H	2	7	1	China
SAMN04166470	41	PRJNA298752	H	2	7	1	China
SAMN04166471	41	PRJNA298752	H	2	7	1	China
SAMN04166472	41	PRJNA298752	H	2	7	1	China
SAMN04166473	41	PRJNA298752	H	2	7	1	China
SAMN04166474	41	PRJNA298752	H	2	7	1	China
SAMN04166475	41	PRJNA298752	H	2	7	1	China
SAMN04166476	41	PRJNA298752	H	2	7	1	China
SAMN04166477	41	PRJNA298752	H	2	7	1	China
SAMN04166478	41	PRJNA298752	H	2	7	1	China
SAMN04166479	41	PRJNA298752	H	2	7	1	China
SAMN04166480	41	PRJNA298752	H	2	7	1	China
SAMN04166481	41	PRJNA298752	H	2	7	1	China
SAMN04166482	41	PRJNA298752	H	2	7	1	China
SAMN04166483	41	PRJNA298752	H	2	7	1	China
SAMN04166484	41	PRJNA298752	H	2	7	1	China
SAMN04166485	41	PRJNA298752	PD	2	7	1	China
SAMN04166486	41	PRJNA298752	PD	2	7	1	China
SAMN04166487	41	PRJNA298752	PD	2	7	1	China
SAMN04166488	41	PRJNA298752	H	2	7	1	China
SAMN04166489	41	PRJNA298752	PD	2	7	1	China
SAMN04166490	41	PRJNA298752	H	2	7	1	China
SAMN04166491	41	PRJNA298752	H	2	7	1	China
SAMN04166492	41	PRJNA298752	H	2	7	1	China
SAMN05231907	42	PRJNA325429	H	2	658	1	China
SAMN05442334	43	PRJNA335427	H	2	1	1	Argentina
SAMN05442335	43	PRJNA335427	H	2	1	1	Argentina
SAMN05442336	43	PRJNA335427	H	2	1	1	Argentina
SAMN05442337	43	PRJNA335427	PD	Unknown	28	2	Argentina
SAMN05442338	43	PRJNA335427	H	2	1	1	Argentina
SAMN05442339	43	PRJNA335427	H	2	1	1	Argentina
SAMN05442340	43	PRJNA335427	H	2	1	1	Argentina
SAMN05442341	43	PRJNA335427	H	2	1	1	Argentina

SAMN05442342	43	PRJNA335427	H	2	1	1	Argentina
SAMN05442343	43	PRJNA335427	H	2	1	1	Argentina
SAMN05442344	43	PRJNA335427	H	2	1	1	China
SAMN05567566	44	PRJNA337985	PD	9	243	48	China
SAMN05919895	45	PRJNA349071	PD	9	125	0	Spain
SAMN05919896	45	PRJNA349071	PD	9	123	0	Spain
SAMN05919897	45	PRJNA349071	PD	9	125	0	Spain
SAMN05919898	45	PRJNA349071	PD	9	123	0	Spain
SAMN05919899	45	PRJNA349071	PD	9	123	0	Spain
SAMN05919900	45	PRJNA349071	PD	9	123	0	Spain
SAMN05919901	45	PRJNA349071	PD	9	791	0	Spain
SAMN05919902	45	PRJNA349071	PD	9	730	88	Brazil
SAMN05919903	45	PRJNA349071	PD	9	730	88	Brazil
SAMN05919904	45	PRJNA349071	PD	9	16	4	Brazil
SAMN05919905	45	PRJNA349071	PD	9	731	89	Canada
SAMN05919906	45	PRJNA349071	PD	9	732	90	Canada
SAMN05919907	45	PRJNA349071	PD	9	788	97	Canada
SAMN05919908	45	PRJNA349071	PD	9	788	97	Canada
SAMN05919909	45	PRJNA349071	PD	9	733	91	Canada
SAMN05919910	45	PRJNA349071	PD	9	734	23	Canada
SAMN05919911	45	PRJNA349071	PD	9	622	85	Canada
SAMN05919912	45	PRJNA349071	PD	9	788	97	Canada
SAMN05919913	45	PRJNA349071	PD	9	621	84	Canada
SAMN05919914	45	PRJNA349071	PD	9	788	97	Canada
SAMN05919915	45	PRJNA349071	PD	9	788	97	Canada
SAMN05919916	45	PRJNA349071	PD	9	Unknown	Unknown	Canada
SAMN05919918	45	PRJNA349071	PD	9	16	4	Canada
SAMN05919919	45	PRJNA349071	PD	9	623	86	Canada
SAMN05919920	45	PRJNA349071	PD	9	621	84	Canada
SAMN05919921	45	PRJNA349071	PD	9	735	92	Canada
SAMN05919922	45	PRJNA349071	PD	9	789	16	Canada
SAMN05919923	45	PRJNA349071	PD	9	622	85	Canada
SAMN05919924	45	PRJNA349071	PD	9	Unknown	Unknown	Canada
SAMN05919925	45	PRJNA349071	PD	9	790	98	Canada
SAMN05942098	46	PRJNA350579	PD	2	7	1	China
SAMN07807408	47	PRJNA414694	PH	2	28	2	China
SAMN06765615	48	PRJNA383459	PD	2	7	1	China
SAMN08435267	49	PRJNA432119	H	2	665	1	China
SAMN08435268	49	PRJNA432119	H	2	1	1	China
SAMN08435269	49	PRJNA432119	H	2	1	1	China
SAMN08435270	49	PRJNA432119	H	2	1	1	China
SAMN08799542	50	PRJNA445803	H	Unknown	1	1	Italy
SAMN11854144	51	PRJNA544735	PD	Unknown	108	0	USA
SAMN11854145	51	PRJNA544735	PD	2	961	2	USA
SAMN11854146	51	PRJNA544735	PD	2	25	2	USA
SAMN11854147	51	PRJNA544735	PD	6	949	22	USA
SAMN11854148	51	PRJNA544735	PD	2	28	2	USA
SAMN11854149	51	PRJNA544735	PD	1	13	9	USA
SAMN11854150	51	PRJNA544735	PD	Unknown	Unknown	Unknown	USA
SAMN11854151	51	PRJNA544735	PD	7	225	5	USA
SAMN11854152	51	PRJNA544735	PD	3	108	0	USA
SAMN11854153	51	PRJNA544735	PD	2	28	2	USA
SAMN11854154	51	PRJNA544735	PD	7	225	5	USA
SAMN11854155	51	PRJNA544735	PD	2	28	2	USA
SAMN11854156	51	PRJNA544735	PD	2	28	2	USA
SAMN11854157	51	PRJNA544735	PD	23	108	0	USA
SAMN11854158	51	PRJNA544735	PD	2	28	2	USA
SAMN11854159	51	PRJNA544735	PD	2	28	2	USA
SAMN11854160	51	PRJNA544735	PD	2	28	2	USA
SAMN11854161	51	PRJNA544735	PD	23	108	0	USA
SAMN11854162	51	PRJNA544735	PD	Unknown	1	1	USA
SAMN11854163	51	PRJNA544735	PD	2	Unknown	Unknown	USA
SAMN11854164	51	PRJNA544735	PD	23	108	0	USA
SAMN11854165	51	PRJNA544735	PD	23	108	0	USA
SAMN11854166	51	PRJNA544735	PD	2	28	2	USA
SAMN11854167	51	PRJNA544735	PD	Unknown	28	2	USA
SAMN11854168	51	PRJNA544735	PD	2	28	2	USA
SAMN11854169	51	PRJNA544735	PD	Unknown	94	0	USA
SAMN11854170	51	PRJNA544735	PD	2	28	2	USA
SAMN11854171	51	PRJNA544735	PD	2	28	2	USA
SAMN11854172	51	PRJNA544735	PD	2	28	2	USA
SAMN11854173	51	PRJNA544735	PD	2	961	2	USA
SAMN11854174	51	PRJNA544735	PD	2	961	2	USA
SAMN11854175	51	PRJNA544735	PD	2	28	2	USA
SAMN11854176	51	PRJNA544735	PD	Unknown	981	174	USA
SAMN11854177	51	PRJNA544735	PD	2	28	2	USA
SAMN11854178	51	PRJNA544735	PD	Unknown	1	1	USA
SAMN11854179	51	PRJNA544735	PD	Unknown	1	1	USA
SAMN11854180	51	PRJNA544735	PD	23	108	0	USA
SAMN11854181	51	PRJNA544735	PD	7	94	0	USA
SAMN11854182	51	PRJNA544735	PD	7	94	0	USA
SAMN11854183	51	PRJNA544735	PD	7	94	0	USA
SAMN11854184	51	PRJNA544735	PD	Unknown	94	0	USA
SAMN11854185	51	PRJNA544735	PD	2	28	2	USA
SAMN11854186	51	PRJNA544735	PD	Unknown	28	2	USA
SAMN11854187	51	PRJNA544735	PD	7	94	0	USA
SAMN11854188	51	PRJNA544735	PD	7	94	0	USA
SAMN11854189	51	PRJNA544735	PD	Unknown	993	182	USA
SAMN11854190	51	PRJNA544735	PD	Unknown	983	175	USA
SAMN11854191	51	PRJNA544735	PD	Unknown	977	0	USA
SAMN11854192	51	PRJNA544735	PD	7	980	0	USA
SAMN11854193	51	PRJNA544735	PD	8	87	3	USA
SAMN11854194	51	PRJNA544735	PD	2	961	2	USA
SAMN11854195	51	PRJNA544735	PD	3	27	2	USA
SAMN11854196	51	PRJNA544735	PD	Unknown	984	176	USA
SAMN11854197	51	PRJNA544735	PD	2	28	2	USA
SAMN11854198	51	PRJNA544735	PD	Unknown	992	7	USA
SAMN11854199	51	PRJNA544735	PD	Unknown	94	0	USA
SAMN11854200	51	PRJNA544735	PD	Unknown	997	185	USA
SAMN11854201	51	PRJNA544735	PD	Unknown	974	23	USA
SAMN11854202	51	PRJNA544735	PD	2	28	2	USA
SAMN11854203	51	PRJNA544735	PD	5	977	0	USA
SAMN11854204	51	PRJNA544735	PD	21	998	186	USA
SAMN11854205	51	PRJNA544735	PD	23	962	0	USA
SAMN11854206	51	PRJNA544735	PD	Unknown	996	184	USA
SAMN11854207	51	PRJNA544735	PD	2	28	2	USA
SAMN11854208	51	PRJNA544735	PD	8	87	3	USA
SAMN11854209	51	PRJNA544735	PD	12	963	167	USA
SAMN11854210	51	PRJNA544735	PD	5	977	0	USA
SAMN11854211	51	PRJNA544735	PD	2	28	2	USA
SAMN11854212	51	PRJNA544735	PD	Unknown	28	2	USA
SAMN11854213	51	PRJNA544735	PD	Unknown	13	9	USA
SAMN11854214	51	PRJNA544735	PD	2	28	2	USA
SAMN11854215	51	PRJNA544735	PD	8	87	3	USA
SAMN11854216	51	PRJNA544735	PD	2	28	2	USA
SAMN11854217	51	PRJNA544735	PD	7	973	2	USA
SAMN11854218	51	PRJNA544735	PD	23	108	0	USA
SAMN11854219	51	PRJNA544735	PD	Unknown	964	0	USA
SAMN11854220	51	PRJNA544735	PD	2	28	2	USA

SAMN11854221	51	PRJNA544735	PD	Unknown	13	9	USA
SAMN11854222	51	PRJNA544735	PD	2	28	2	USA
SAMN11854223	51	PRJNA544735	PD	2	28	2	USA
SAMN11854224	51	PRJNA544735	PD	2	28	2	USA
SAMN11854225	51	PRJNA544735	PD	2	28	2	USA
SAMN11854226	51	PRJNA544735	PD	Unknown	17	6	USA
SAMN11854227	51	PRJNA544735	PD	2	28	2	USA
SAMN11854228	51	PRJNA544735	PD	2	28	2	USA
SAMN11854229	51	PRJNA544735	PD	2	982	0	USA
SAMN11854230	51	PRJNA544735	PD	3	119	0	USA
SAMN11854231	51	PRJNA544735	PD	2	28	2	Mexico
SAMN11854232	51	PRJNA544735	PD	7	94	0	USA
SAMN11854233	51	PRJNA544735	PD	1	1	1	USA
SAMN11854234	51	PRJNA544735	PD	7	373	0	USA
SAMN11854235	51	PRJNA544735	PD	5	977	0	USA
SAMN11854236	51	PRJNA544735	PD	2	28	2	USA
SAMN11854237	51	PRJNA544735	PD	7	94	0	USA
SAMN11854238	51	PRJNA544735	PD	2	28	2	USA
SAMN11854239	51	PRJNA544735	PD	7	225	5	USA
SAMN11854240	51	PRJNA544735	PD	2	28	2	USA
SAMN11854241	51	PRJNA544735	PD	1	1	1	USA
SAMN11854242	51	PRJNA544735	PD	9	373	0	USA
SAMN11854243	51	PRJNA544735	PD	Unknown	13	9	USA
SAMN11854244	51	PRJNA544735	PD	2	28	2	USA
SAMN11854245	51	PRJNA544735	PD	2	25	2	USA
SAMN11854246	51	PRJNA544735	PD	5	977	0	USA
SAMN11854247	51	PRJNA544735	PD	7	94	0	USA
SAMN11854248	51	PRJNA544735	PD	7	94	0	USA
SAMN11854249	51	PRJNA544735	PD	2	28	2	USA
SAMN11854250	51	PRJNA544735	PD	2	1	1	USA
SAMN11854251	51	PRJNA544735	PD	2	1	1	USA
SAMN11854252	51	PRJNA544735	PD	5	977	0	USA
SAMN11854253	51	PRJNA544735	PD	Multiple	Unknown	Unknown	USA
SAMN11854254	51	PRJNA544735	PD	3	117	2	USA
SAMN11854255	51	PRJNA544735	PD	2	28	2	USA
SAMN11854256	51	PRJNA544735	PD	Unknown	28	2	USA
SAMN11854257	51	PRJNA544735	PD	7	979	173	USA
SAMN11854258	51	PRJNA544735	PD	Unknown	13	9	USA
SAMN11854259	51	PRJNA544735	PD	7	373	0	USA
SAMN11854260	51	PRJNA544735	PD	2	1	1	USA
SAMN11854261	51	PRJNA544735	PD	9	373	0	USA
SAMN11854262	51	PRJNA544735	PD	5	119	0	USA
SAMN11854263	51	PRJNA544735	PD	5	94	0	USA
SAMN11854264	51	PRJNA544735	PD	2	28	2	USA
SAMN11854265	51	PRJNA544735	PD	3	108	0	USA
SAMN11854266	51	PRJNA544735	PD	9	965	168	USA
SAMN11854267	51	PRJNA544735	PD	5	94	0	USA
SAMN11854268	51	PRJNA544735	PD	8	87	3	USA
SAMN11854269	51	PRJNA544735	PD	2	Unknown	Unknown	USA
SAMN11854270	51	PRJNA544735	PD	1	1	1	USA
SAMN11854271	51	PRJNA544735	PD	7	29	2	USA
SAMN11854272	51	PRJNA544735	PD	7	29	2	USA
SAMN11854273	51	PRJNA544735	PH	Unknown	966	0	USA
SAMN11854274	51	PRJNA544735	PH	31	975	170	USA
SAMN11854275	51	PRJNA544735	PH	9	994	183	USA
SAMN11854276	51	PRJNA544735	PH	12	1001	187	Canada
SAMN11854277	51	PRJNA544735	PH	5	Unknown	Unknown	Canada
SAMN11854278	51	PRJNA544735	PH	7	971	169	Canada
SAMN11854279	51	PRJNA544735	PH	29	972	7	Canada
SAMN11854280	51	PRJNA544735	PD	2	28	2	USA
SAMN11854281	51	PRJNA544735	PD	Unknown	23	3	USA
SAMN11854282	51	PRJNA544735	PD	2	28	2	USA
SAMN11854283	51	PRJNA544735	PD	2	28	2	USA
SAMN11854284	51	PRJNA544735	PD	23	108	0	USA
SAMN11854285	51	PRJNA544735	PD	2	28	2	USA
SAMN11854286	51	PRJNA544735	PD	Unknown	108	0	USA
SAMN11854287	51	PRJNA544735	PD	Unknown	1	1	USA
SAMN11854288	51	PRJNA544735	PD	Unknown	1	1	USA
SAMN11854289	51	PRJNA544735	PD	2	961	2	USA
SAMN11854290	51	PRJNA544735	PD	2	961	2	USA
SAMN11854291	51	PRJNA544735	PD	3	108	0	USA
SAMN11854292	51	PRJNA544735	PD	2	961	2	USA
SAMN11854293	51	PRJNA544735	PD	5	977	0	USA
SAMN11854294	51	PRJNA544735	PD	2	961	2	USA
SAMN11854295	51	PRJNA544735	PD	1	Unknown	Unknown	USA
SAMN11854296	51	PRJNA544735	PD	Unknown	117	2	USA
SAMN11854297	51	PRJNA544735	PD	Unknown	Unknown	Unknown	USA
SAMN11854298	51	PRJNA544735	PD	8	87	3	USA
SAMN11854299	51	PRJNA544735	PD	9	976	171	USA
SAMN11854300	51	PRJNA544735	PD	Unknown	1	1	USA
SAMN11854301	51	PRJNA544735	PD	7	94	0	USA
SAMN11854302	51	PRJNA544735	PH	21	821	109	USA
SAMN11854305	51	PRJNA544735	PD	1	1	1	USA
SAMN11854309	51	PRJNA544735	PD	Unknown	108	0	USA
SAMN11854310	51	PRJNA544735	PD	5	978	172	USA
SAMN11854311	51	PRJNA544735	PD	10	986	177	USA
SAMN11854312	51	PRJNA544735	PD	2	28	2	USA
SAMN11854313	51	PRJNA544735	PD	8	87	3	USA
SAMN11854314	51	PRJNA544735	PD	3	839	0	USA
SAMN11854316	51	PRJNA544735	PD	Unknown	961	2	USA
SAMN11854318	51	PRJNA544735	PD	Unknown	961	2	USA
SAMN11854319	51	PRJNA544735	PD	7	94	0	USA
SAMN11854321	51	PRJNA544735	PD	5	977	0	USA
SAMN11854322	51	PRJNA544735	PD	8	87	3	USA
SAMN11854323	51	PRJNA544735	PD	2	28	2	USA
SAMN11854324	51	PRJNA544735	PD	2	28	2	USA
SAMN11854325	51	PRJNA544735	PD	2	28	2	USA
SAMN11854326	51	PRJNA544735	PH	Unknown	108	0	USA
SAMN11854327	51	PRJNA544735	PH	21	821	109	USA
SAMN11854328	51	PRJNA544735	PH	31	988	179	USA
SAMN11854329	51	PRJNA544735	PH	Unknown	750	7	USA
SAMN11854330	51	PRJNA544735	PH	Unknown	750	7	USA
SAMN11854331	51	PRJNA544735	PH	6	948	22	USA
SAMN11854332	51	PRJNA544735	PH	9	987	178	USA
SAMN11854333	51	PRJNA544735	PH	Unknown	94	0	USA
SAMN11854334	51	PRJNA544735	PH	28	968	24	USA
SAMN11854335	51	PRJNA544735	PH	21	821	109	USA
SAMN11854336	51	PRJNA544735	PH	21	821	109	USA
SAMN11854337	51	PRJNA544735	PH	8	87	3	USA
SAMN11854338	51	PRJNA544735	PD	2	1	1	USA
SAMN11854339	51	PRJNA544735	PD	2	28	2	USA
SAMN11854340	51	PRJNA544735	PD	7	373	0	USA
SAMN11854341	51	PRJNA544735	PD	Unknown	991	181	USA
SAMN11854342	51	PRJNA544735	PD	Unknown	1	1	USA
SAMN11854344	51	PRJNA544735	PD	31	989	180	USA
SAMN11854345	51	PRJNA544735	PD	Unknown	28	2	USA
SAMN11854346	51	PRJNA544735	PD	5	87	3	USA
SAMN11854347	51	PRJNA544735	PD	2	28	2	USA
SAMN11854348	51	PRJNA544735	PH	Unknown	750	7	USA

SAMN11854349	51	PRJNA544735	PH	Unknown	750	7	USA
SAMN11854350	51	PRJNA544735	PD	Unknown	969	0	USA
SAMN11854351	51	PRJNA544735	PD	3	969	0	USA
SAMN02691834	Unknown	PRJNA242223	PD	2	7	1	China
SAMN05912964	Unknown	PRJNA348621	PH	31	Unknown	Unknown	China
SAMN05912965	Unknown	PRJNA348622	PH	31	Unknown	Unknown	China
SAMN06232410	Unknown	PRJNA361367	PD	Unknown	Unknown	Unknown	Canada
SAMN06232411	Unknown	PRJNA361367	PD	Unknown	736	15	Canada
SAMN06232412	Unknown	PRJNA361367	PD	Unknown	741	93	Canada
SAMN06232413	Unknown	PRJNA361367	PD	Unknown	Unknown	Unknown	Canada
SAMN06232414	Unknown	PRJNA361367	PD	Unknown	741	93	Canada
SAMN06232420	Unknown	PRJNA361367	PD	Unknown	Unknown	Unknown	Canada
SAMN06232422	Unknown	PRJNA361367	PD	2	1103	1	Canada
SAMN06232423	Unknown	PRJNA361367	PD	Unknown	751	94	Canada
SAMN06232425	Unknown	PRJNA361367	PD	Unknown	Unknown	Unknown	Canada
SAMN06232427	Unknown	PRJNA361367	PD	29	Unknown	Unknown	Canada
SAMN06232429	Unknown	PRJNA361367	PD	27	Unknown	Unknown	Canada
SAMN06232431	Unknown	PRJNA361367	PD	5	Unknown	Unknown	Canada
SAMN06232432	Unknown	PRJNA361367	PD	Unknown	Unknown	Unknown	Canada
SAMN06232433	Unknown	PRJNA361367	PD	11	Unknown	Unknown	Canada
SAMN06232434	Unknown	PRJNA361367	PD	11	Unknown	Unknown	Canada
SAMN06232435	Unknown	PRJNA361367	PD	15	Unknown	Unknown	Canada
SAMN06232436	Unknown	PRJNA361367	PD	15	Unknown	Unknown	Canada
SAMN06232437	Unknown	PRJNA361367	PD	15	Unknown	Unknown	Canada
SAMN06232438	Unknown	PRJNA361367	PD	Unknown	Unknown	Unknown	Canada
SAMN06232441	Unknown	PRJNA361367	PD	2	28	2	Canada
SAMN06232442	Unknown	PRJNA361367	PD	2	28	2	Canada
SAMN06232443	Unknown	PRJNA361367	PD	Unknown	28	2	Canada
SAMN06232444	Unknown	PRJNA361367	PD	Unknown	28	2	Canada
SAMN06232445	Unknown	PRJNA361367	PD	Unknown	28	2	Canada
SAMN06232446	Unknown	PRJNA361367	PD	Unknown	28	2	Canada
SAMN08238443	Unknown	PRJNA427455	H	2	7	1	China
SAMN08238502	Unknown	PRJNA427457	H	2	7	1	China
SAMN08238506	Unknown	PRJNA427459	H	2	7	1	China
SAMN08374181	Unknown	PRJNA430485	PD	9	243	48	China
SAMN08374182	Unknown	PRJNA430485	PD	2	1	1	China
SAMN09460428	Unknown	PRJNA476843	PD	4	94	0	China
SAMN09460429	Unknown	PRJNA476845	PH	4	1006	188	China
SAMN13404064	Unknown	PRJNA592036	PH	2	1	1	China

Legend

H=Human PD=Pig Diseased PH=Pig Healthy

References

1 Okura M, Nozawa T, Watanabe T, et al. A Locus Encoding Variable Defense Systems against Invading DNA Identified in Streptococcus suis. Genome Biol Evol
2 Willemse N, van der Ark KCH, Stockhofe-Zurwieden N, et al. Clonal expansion of a virulent Streptococcus suis serotype 9 lineage distinguishable from carriage
3 Jolley KA, Maiden MCJ. BIGSdb: Scalable analysis of bacterial genome variation at the population level. BMC Bioinformatics 2010; 11: 595.
4 Weinert LA, Chaudhuri RR, Wang J, et al. Genomic signatures of human and animal disease in the zoonotic pathogen Streptococcus suis. Nat Commun 2015; 6:
5 Hill DMC, Lucidarme J, Gray SJ, et al. Genomic epidemiology of age-associated meningococcal lineages in national surveillance: an observational cohort study.
6 Holden MTG, Hauser H, Sanders M, et al. Rapid Evolution of Virulence and Drug Resistance in the Emerging Zoonotic Pathogen Streptococcus suis. PLoS One
7 Berthelot-Hérault F, Gottschalk M, Morvan H, Kobisch M. Dilemma of virulence of Streptococcus suis: Canadian isolate 89-1591 characterized as a virulent
8 Chen C, Tang J, Dong W, et al. A Glimpse of Streptococcal Toxic Shock Syndrome from Comparative Genomics of S. suis 2 Chinese Isolates. PLoS One 2007;
9 Chen C, Zhang W, Zheng H, et al. Minimum core genome sequence typing of bacterial pathogens: a unified approach for clinical and public health microbiology.
10 Hu P, Yang M, Zhang A, et al. Comparative Genomics Study of Multi-Drug-Resistance Mechanisms in the Antibiotic-Resistant Streptococcus suis R61 Strain.
11 Wang K, Yao H, Lu C, Chen J. Complete Genome Sequence of Streptococcus suis Serotype 16 Strain TL13. Genome Announc 2013; 1: e00394-13.
12 Wang K, Chen J, Yao H, Lu C. Whole-Genome Sequence of Streptococcus suis Serotype 3 Strain YB51. Genome Announc 2013; 1: e00884-13.
13 Hu P, Yang M, Zhang A, et al. Complete Genome Sequence of <span class="named-content genus-species" id="named-content-1"
14 Hu P, Yang M, Zhang A, et al. Complete Genome Sequence of <span class="named-content genus-species" id="named-content-1"
15 Wu Z, Wang W, Tang M, et al. Comparative genomic analysis shows that Streptococcus suis meningitis isolate SC070731 contains a unique 105K genomic
16 Wu Z, Li M, Wang C, et al. Probing genomic diversity and evolution of Streptococcus suis serotype 2 by NimbleGen tiling arrays. BMC Genomics 2011; 12:
17 Ye C, Zheng H, Zhang J, et al. Clinical, Experimental, and Genomic Differences between Intermediately Pathogenic, Highly Pathogenic, and Epidemic
18 Wang K, Chen J, Yao H, Lu C. Whole-Genome Sequence of Streptococcus suis Serotype 4 Reference Strain 6407. Genome Announc 2014; 2: e00770-14.
19 Hau SJ, Bayles DO, Alt DP, Brockmeier SL, Frana TS, Nicholson TL. Draft Genome Sequences of Nine Streptococcus suis Strains Isolated in the United States.
20 Athey TBT, Auger J-P, Teatero S, et al. Complex Population Structure and Virulence Differences among Serotype 2 Streptococcus suis Strains Belonging to
21 LeBel G, Vaillancourt K, Frenette M, Gottschalk M, Grenier D. Suicin 90-1330 from a nonvirulent strain of Streptococcus suis: a nisin-related lantibiotic active
22 Athey TBT, Teatero S, Takamatsu D, et al. Population Structure and Antimicrobial Resistance Profiles of Streptococcus suis Serotype 2 Sequence Type 25
23 Athey TBT, Teatero S, Lacouture S, Takamatsu D, Gottschalk M, Fittipaldi N. Determining Streptococcus suis serotype from short-read whole-genome
24 Hu Y, Hu Q, Wei R, et al. The XRE Family Transcriptional Regulator SrtR in Streptococcus suis Is Involved in Oxidant Tolerance and Virulence. Front Cell
25 Zhang Y, Lu P, Pan Z, et al. SssP1, a Streptococcus suis Fimbria-Like Protein Transported by the SecY2/A2 System, Contributes to Bacterial Virulence. Appl
26 Zou G, Zhou J, Xiao R, et al. Effects of Environmental and Management-Associated Factors on Prevalence and Diversity of Streptococcus suis in Clinically
27 Zhu Y, Zhang Y, Ma J, et al. ICESsuHN105, a Novel Multiple Antibiotic Resistant ICE in Streptococcus suis Serotype 5 Strain HN105. Front Microbiol 2019;
28 O'Dea MA, Laird T, Abraham R, et al. Examination of Australian Streptococcus suis isolates from clinically affected pigs in a global context and the genomic
29 Niemann L, Eichhorn I, Müller P, et al. Draft Genome Sequences of Three Porcine Streptococcus suis Isolates Which Differ in Their Susceptibility to Penicillin.
30 Stevens MJA, Spoerry Serrano N, Cernela N, Schmitt S, Schrenzel J, Stephan R. Massive Diversity in Whole-Genome Sequences of Streptococcus suis Strains
31 Huang J, Liu X, Chen H, et al. Identification of six novel capsular polysaccharide loci (NCL) from Streptococcus suis multidrug resistant non-typeable strains
32 Rui L, Weiyi L, Yu M, et al. The serine/threonine protein kinase of Streptococcus suis serotype 2 affects the ability of the pathogen to penetrate the blood-brain
33 Tohya M, Watanabe T, Maruyama F, et al. Comparative Genome Analyses of Streptococcus suis Isolates from Endocarditis Demonstrate Persistence of Dual
34 Taniyama D, Sakurai M, Sakai T, Kikuchi T, Takahashi T. Human case of bacteremia due to Streptococcus suis serotype 5 in Japan: The first report and
35 Hayashi T, Tsukagoshi H, Sekizuka T, et al. Next-generation DNA sequencing analysis of two Streptococcus suis ST28 isolates associated with human infective
36 Willemse N, van der Ende A, Schultsz C. Reinfection with Streptococcus suis analysed by whole genome sequencing. Zoonoses Public Health 2019; 66: 179–83.
37 Willemse N, Howell KJ, Weinert LA, et al. An emerging zoonotic clone in the Netherlands provides clues to virulence and zoonotic potential of Streptococcus
38 Tall H, Njanpop-Lafourcade B-M, Mounkoro D, et al. Identification of Streptococcus suis Meningitis through Population-Based Surveillance, Togo,
39 Gaiser RA, Zomer AL, Wells JM, van Baarlen P. Draft Genome Sequence of <span class="named- content genus-species" id="named-conte
40 van der Putten BCL, Roodsant TJ, Haagmans MA, Schultsz C, van der Ark KCH. Five Complete Genome Sequences Spanning the Dutch <span class="
41 Du P, Zheng H, Zhou J, et al. Detection of Multiple Parallel Transmission Outbreak of Streptococcus suis Human Infection by Use of Genome Epidemiology,
42 Zhou Y, Dong X, Li Z, et al. Predominance of Streptococcus suis ST1 and ST7 in human cases in China, and detection of a novel sequence type, ST658.
43 Callejo R, Zheng H, Du P, et al. Streptococcus suis serotype 2 strains isolated in Argentina (South America) are different from those recovered in North America
44 Lai L, Dai J, Tang H, et al. Streptococcus suis serotype 9 strain GZ0565 contains a type VII secretion system putative substrate EsxA that contributes to bacterial
45 Zheng H, Du P, Qiu X, et al. Genomic comparisons of Streptococcus suis serotype 9 strains recovered from diseased pigs in Spain and Canada. Vet Res 2018; 49
46 Teng L, Dong X, Zhou Y, et al. Draft Genome Sequence of Hypervirulent and Vaccine Candidate <span class="named-content genus-species" id=
47 Zhu H, Ni Y, Zhou J, et al. Complete Genome Sequence of Streptococcus suis Serotype 2 Virulent Strain SS2-1. Genome Announc 2018; 6: e00067-18.
48 Li W, Liu L, Qiu D, Chen H, Zhou R. Identification of Streptococcus suis serotype 2 genes preferentially expressed in the natural host. Int J Med Microbiol 2010
49 Huang W, Wang M, Hao H, et al. Genomic epidemiological investigation of a Streptococcus suis outbreak in Guangxi, China, 2016. Infect Genet Evol 2019; 68:
50 Fillo S, Mancini F, Anselmo A, et al. Draft Genome Sequence of Streptococcus suis Strain SsRC-1, a Human Isolate from a Fatal Case of Toxic Shock
51 Estrada AA, Gottschalk M, Rossow S, Rendahl A, Gebhart C, Marthaler DG. Serotype and Genotype (Multilocus Sequence Type) of Streptococcus suis Isolates
